# The case for change: aviation worker wellbeing during the COVID 19 pandemic, and the need for an integrated health and safety culture

**DOI:** 10.1007/s10111-022-00711-5

**Published:** 2022-08-17

**Authors:** Joan Cahill, Paul Cullen, Keith Gaynor

**Affiliations:** 1 Centre for Innovative Human Systems, School of Psychology, Trinity College Dublin (TCD), Dublin, Ireland; 2 School of Psychology, University College Dublin (UCD), Dublin, Ireland

**Keywords:** Safety, Wellbeing, Mental health, Aviation workers, Airline safety management systems, Culture

## Abstract

The workplace is an important setting for health protection, health promotion and disease prevention. Currently, health and wellbeing approaches at an aviation organisational level are not addressing both human and safety needs. This issue has been intensified since the COVID 19 pandemic. This paper reports on the findings of a survey pertaining to aviation worker wellbeing and organisational approaches to managing wellbeing and mental health. The survey was administered at two different time periods during the COVID 19 pandemic (2020 and 2021). Collectively, feedback was obtained from over 3000 aviation workers. Survey feedback indicates that aviation workers are experiencing considerable challenges in relation to their health and wellbeing. These challenges are not being adequately addressed at an organisational level, which creates risk both from an individual and flight safety perspective. The descriptive findings of both surveys along with a regression analysis is used to make a principled case for augmenting the existing approach to managing aviation worker wellbeing (including mental health), at both an organisational and regulatory level. It is argued that aviation organisations, with the support of the regulator should implement a preventative, ethical and evidence-based strategy to managing wellbeing and mental health risk. Critically, aviation organisations need to advance and integrated health, wellbeing, and safety culture. This necessitates an alignment of human, business, and safety objectives, as articulated in concepts of corporate social responsibility (CSR) and responsible work. Critically, this approach depends on trust and the specification of appropriate protections, so that aviation workers feel safe to routinely report wellbeing levels and challenges, and their impact on operational safety.

## Introduction

Work is part of our wellbeing and a key driver of a person’s health. Stress arising from the job and/or the management of the home/work interface can compromise a person’s ability to perform their job (Allen et al. 2000). Increasingly, there is greater acceptance of work-related stress (WRS) as an organisational issue and not an individual fault. In addition, there has been a greater focus on ‘work culture’—both as a component of work stress, and a key to creating effective wellness interventions (Peterson and Wilson 2002).

Worker health, wellbeing and stress management is of critical importance in high reliability safety critical systems such as civil aviation. Evidence from the safety culture/climate literature identifies the importance of elements of wellbeing as a factor in employee safety performance (Kristensen et al. 2015; Kompier and Kristensen 2001). According to the European Aviation Safety Authority (EASA) accident statistics, approximately a quarter of commercial air transport large aeroplane accident and serious incident reports identify human factors (HF) or human performance (HP) issues (EASA 2020). The accident cause classification scheme includes ‘State of Wellbeing & Fitness for Duty’. Crucially, ‘state of wellbeing and fitness for duty’ is defined as a priority 1 core safety issue.

Currently, health and wellbeing approaches at an aviation organisational level are not addressing both human and safety needs. Prior to the COVID pandemic, there was ample evidence of work stress issues impacting on pilot wellbeing (Demerouti et al. 2018; Cahill et al. 2018; Cullen et al. 2021). This includes disengagement, burnout, and depression amongst pilots (Wu et al. 2016; Demerouti et al. 2018; Cahill et al. 2018, 2020a, b, 2021). Following the Germanwings tragedy in 2015, the European Union Aviation Safety Agency (EASA) introduced new rules in relation to the management of pilot mental fitness (EASA 2019). These rules pertain to three key areas—psychological testing of aircrew pre-employment in line flight, access to a psychological support/peer support resource, and substance abuse testing on a random basis. Overall, the focus has been on addressing those who are suffering. However, many argue that there has been an insufficient emphasis on prevention and the promotion of positive wellbeing. Further, there has been insufficient focus on the wellbeing of other aviation workers. As argued by the British Psychological Society, the EASA recommendations to support pilot mental health, apply to the wider team and network of aviation personnel that pilots interact with (BPS 2017).

This issue is now more urgent given the COVID 19 context. The COVID 19 pandemics has had a huge impact on aviation workers and the aviation industry (Flight Safety Foundation 2020). Many aviation workers have lost their jobs and/or experiencing reduced pay. As has been highlighted in recent COVID studies, financial insecurity/job loss is a risk factor for depression and anxiety (Hyland et al. 2021a, b). The Flight Safety Foundation (FSF) highlight three groups of aviation workers who require support—those in work, those off work and those returning to work (FSF 2020). EASA point to specific safety issues associated with the pandemic such as the risk of skills and knowledge degradation due to lack of recent practice, and risks associated with the wellbeing of aviation professionals (2021). These issues have also been highlighted by the wider aviation community. According to the International Civil Aviation Organisation (ICAO), “Much more than a disease caused by a virus, the COVID-19 pandemic is also proving to be a psychological contamination” (ICAO 2021). ICAO have cautioned “The crisis affecting the survival of the aviation industry is a major source of stress, anxiety and decrease in motivation and alertness of the personnel with the fear of the loss of their livelihood. The resulting impact may have a direct consequence on the aviation safety if appropriate steps are not taken in a structured and proactive manner” (ICAO 2021).

Full cost accounting and the ‘Triple Bottom Line’ approach highlights the need for businesses to evaluate performance from the perspective of three cost benefit areas—namely, the economic, ecological, and social pillars (Elkington 1994). As emphasized in the concepts of ‘corporate social responsibility’ and ‘decent work’, employer responsibility spans shareholders, workers, and society (International Labour Organisation 2021). This is also highlighted in ISO 26000, the voluntary guidance standard on social responsibility (International Standards Organisation 2010).

The ‘Ethics of Care’ approach challenges employers to address social justice concerns in the workplace. ‘Care’ is viewed as a foundational aspect of human experience—as a practice, which finds meaning and is motivated by the network of relationships that binds us together (Gilligan 1982). Accordingly, employers are challenged to place a strong emphasis on the importance of ‘inner judgement’ and the ‘personal approach’, instead of external obligations or duties (Tronto 2005).

This paper reports on the findings of a survey pertaining to aviation worker wellbeing and the existing approach to the health and wellbeing management and allied wellbeing culture, at an organisational level. The survey was administered at two different time periods—in 2020 and again in 2021. The findings of the two surveys (*n* = 3000 aviation workers) are analysed and compared. This includes (1) a comparison of survey findings for all worker profiles across the two surveys/timepoints, and (2) a comparison of feedback across different aviation worker profiles. In relation to (2), this spans pilots, cabin crew, air traffic control and maintenance/engineering staff. Further, the results of an additional analysis (regression analysis) undertaken in relation to the 2021 survey is presented. The collective analysis is then used to make an evidenced base case for changing the existing regulatory and aviation organisational approach to managing aviation worker wellbeing.

## Wellbeing: general population, pilots and aviation workers

### Wellbeing, mental health and positive wellbeing

Physical, psychological factors and social factors (including family relationship, social support, working conditions and working environment) are some of the determinants affecting a person’s health and wellbeing (Engel 1977). ‘Psychological wellbeing’ or ‘mental health’ (MH) is a key dimension of wellbeing. Wellbeing can be defined both positively and negatively. The Word Health Organisation (WHO) emphasizes the importance of fostering and maintaining positive wellbeing and reaching one’s potential, as opposed to simply preventing and managing illness (1998). This links to the positive psychology approach which endeavours to increase human flourishing (Seligman 2011).

### Life satisfaction and happiness and prevalence of MH issues

Life satisfaction measures how people evaluate their life as a whole—rather than just their current feelings. According to research undertaken by the Organisation for Economic Co-operation and Development (OECD), when asked to rate their general satisfaction with life on a scale from 0 to 10, people on average across the OECD gave it a score of 6.5 (OECD 2020, 2021).

Common mental disorders refer to two main diagnostic categories: depressive disorders and anxiety disorders. In 2015, the proportion of the global population with depressive disorders was estimated to be 4.4%, while the proportion with anxiety disorders was estimated at 3.6% (WHO 2017). A recent GBD study in 2019 indicates that the point prevalence for depression is approximately 3.6% (Rehm and Shield 2019). The global lifetime prevalence of suicidal ideation in the general population ranges from 3.1 to 5.6% (Nock et al. 2008). In the OECD countries, mental disorders are the second largest cause for work disability (OECD 2010) and their proportion is still increasing (Viola and Moncrieff 2016).

### Aviation worker MH

Most of the studies addressing wellbeing for aviation workers focus on pilots only. A 2016 study of pilot mental health indicated that 12.6% of respondents screened positive for depression on the PHQ 9 (Wu et al. 2016). Further, a systematic review of 20 studies examining depression in airline pilots found that the prevalence of major depressive disorder experienced by commercial airline pilots ranged from 1.9 to 12.6% (Pasha and Stokes 2018). More recently, in an anonymous online survey of commercial pilots conducted during 2018 and early 2019, 17.6% of respondents screened positive for depression on the PHQ 9 (Cahill et al. 2021).

In a 2016 health promotion survey undertaken by ICOA, MH was reported as the most common cause for in flight incapacitation (21%) for professional pilots (Jordan, 2018). However, for ATC, MH ranked sixth at 6%. For pilots, the top reasons/causes for loss of licence included both mental health and cardiovascular issues (22%). For ATC, the top two included cardiovascular (22%) and MH (18%) (Jordan, 2018). In a 1994 study of interactions between workload and psychophysiological stress for ATCs, 10–15% of the ATCs showed elevated values in psychological stress symptoms indicating serious stress problems at work and/or in their private life (Zeier 1994). A 2014 study of the health status of cabin crews and found significantly more sleep problems, depression, anxiety, and fatigue than in the average population (McNeelyet al. 2014). Further, a global study of 116 countries (including Africa, Asia/Pacific, Middle East, North America, Latin/South America, and Europe) undertaken by the International Transport Workers’ Federation (ITWF) highlights the changes in Civil Aviation workers’ conditions between 2000 and 2007. The study emphasizes the global increase in in stress and fatigue for cabin crew, ground staff workers and air traffic service workers (International Transport Workers’ Federation 2009).

### Impact of COVID-19 pandemic on MH

A recent study of the global prevalence and burden of depressive and anxiety disorders in 204 countries and territories in 2020 due to the COVID-19 pandemic has identified an increase of 27.6% in major depressive disorder, and an increase in 25.6% in anxiety disorders (Santamauro et al. 2021). However, a study of the general adult population of the Republic of Ireland that was gathered prior to the outbreak of the COVID-19 pandemic (i.e., February 2019), and again at two intervals during the early phase of the COVID-19 pandemic (i.e., March–April 2020 and April–May 2020), shows a different trend. The numbers of adults screening positive on the PHQ9 for major depression fell from 29.8% in 2019 to 22.8% in 2021. Equally, the numbers of adults screening positive on the GAD 7 for anxiety fell from 22.8% in 2019 to 20% in 2021.

A 2020 study of cabin crew mental health highlights the negative impact of the COVID-19 pandemic on the mental health of cabin crews (Görlich and Stadelmann 2020). According to the study, job insecurity and fear of the future, as well as contact restrictions in general and not being allowed to work, have cumulated in a sharp increase in symptoms of depression, anxiety, and stress. German flight attendants were surveyed online in May 2019 and April 2020. The incidence of clinically relevant symptoms among the respondents increased from 8 to 23% (depression), from 6 to 14% (anxiety), and from 8 to 24% (stress).

## Work-related stress (WRS), risk assessment for WRS and wellness programmes

### Work-related stress (WRS)

As highlighted in the United Nations ‘Sustainable Development Agenda’, the relationship between work and health (including mental health) is mediated by work-related stress (WRS) (2015). WRS is the negative response people have to excessive pressures, or other types of demands placed on them at work. To understand WRS we must consider both (1) context to work factors and (2) content of work factors (Cox and Griffiths, 2005). Context to work refers to potentially hazardous conditions (i.e., organisational culture, role in organisation, career development, decision latitude and control, interpersonal relations at work and the home/work interface). Content of work concerns potentially hazardous demands (i.e., work environment and equipment, task design, workload/pace of work and work schedule) (Cox and Griffiths 2005).

Others argue that the interaction between job demand and job resources is important for the development of both job strain and motivation (Bakker and Demerouti 2007). In a 2010 meta-analysis involving 179 independent studies, Nahrgang, Morgeson, and Hofmann explored the relationship among job demands, job resources, engagement, burnout, and safety outcomes. Stressors in the work environment (including psychosocial stressors) were found to negatively impact on worker engagement, procedural compliance, and safety behaviour (Nahrgang et al. 2011). An analysis of job resources revealed that knowledge, social support, leadership, and safety climate were all significantly related to engagement, compliance, and satisfaction (Nahrgang et al. 2011). In a recent study of burnout in pilots (Demerouti et al. 2018), the authors demonstrate that both work resources (i.e., supportive work environment and development opportunities) and personal resources (i.e., personality traits and coping ability) may buffer the effect of job demands (i.e., home/work conflict and future uncertainty) on job strain, including burnout.

### Risk assessment for WRS and psychological health and safety

The 1989 Council Directive (89/391) introduced by the European Commission makes employers responsible for making sure employees are not harmed by work, including through the effects of WRS (European Agency for Safety and Health in Work 2019). Historically, workplace health and safety initiatives placed more emphasis on physical health and safety issues than on mental health/psychological wellbeing. The Safety, Health and Welfare at Work Act (2005) requires employers to put in place systems of work which protect employees from hazards which could lead to mental or physical ill-health. As stated by the European Agency for Safety and Health in Work, (2019), risk assessment for stress (i.e., psychosocial risk) involves the same basic principles and processes as for other workplace hazards, including the application of the principles of prevention. The hazards must be identified, the risks assessed, and control measures identified, implemented, and evaluated. Further, as defined in the European Framework for Psychosocial Risk Management (PRIMA-EF), (EU 2008), such risk management should address both positive and negative aspects of the work environment. Critically, positive aspects of the work environment (including work supports and resources) should be promoted and supported. Also, as outlined in directive 89/391, workers need to be involved in the identification of hazards/risk. This has led to the promotion of ‘Psychological Health and Safety’, which involves applying risk management principles to the identification and management of psychosocial hazards. The CSA Standard Z1003-13 (R2018) is a voluntary standard that defines a psychologically healthy and safe workplace as a “workplace that promotes workers' psychological well-being and actively works to prevent harm to worker psychological health including in negligent, reckless, or intentional ways” (2018). The management of psychosocial risk is also emphasized in the new international standards on psychological health in work (ISO/FDIS 45003) and safe work during the COVID 19 Pandemic ISO/PAS 45005 (2020).

### Healthy workplace, workplace wellness and wellness programs

The World Health Organisation (WHO) define a healthy workplace as one in which workers and managers collaborate to use a continual improvement process to protect and promote the health, safety and wellbeing of all workers and sustainable operations (2021). This requires interventions across different ‘avenues of influence’ including the physical work environment, the psychosocial work environment, personal health resources, and enterprise involvement in the community (2021).

Employers across different industries are becoming more aware of the financial benefits in relation to addressing work-related stress (including psychosocial stress), addressing wellbeing culture, and ensuring a healthy work environment (Berry et al. 2010). This is evidenced in the growth of workplace wellness programs addressing health promotion and disease prevention (Berry et al. 2010).

Workplace wellness is defined as any workplace health promotion activity or organizational policy designed to support healthy behavior in the workplace and to improve health outcomes (Goetzel and Ozminkowski 2008; Mujtaba and Cavico 2013). While not all workplace wellness programs live up to their potential, the benefits of such programs are well documented. According to the Health Fitness Revolution (2019), benefits include fun, productivity, happier staff, builds community, lower healthcare costs, improved physical fitness, weight loss, less stress, and healthier habits. In most cases, programs focus on health education (for example, training in stress management and managing mental wellbeing), health screening and promoting healthy behavior (for example, providing staff with access to healthy food or wellness activities such as yoga). Such programs are often not integrated with an organisation’s work management policies, procedures, and culture and do not address psychosocial risks in the workplace. Employees may have access to gyms and health food. However, sources of WRS and key psychosocial risks such as working long hours, workload, low decision latitude and poor communication between staff and managers are not addressed. As argued by the CDC (2021), building a workplace health program should involve a coordinated, systematic, and comprehensive approach.

The importance of implementing a preventative approach has been highlighted by Goetzel and Ozminkowski (2008). Goetzel and Ozminkowski (2008) define three levels of workplace wellness programmes—primary, secondary, and tertiary interventions. Primary level interventions, focus on taking action to modify or eliminate sources of psychosocial risks inherent in the workplace and work environment—thereby reducing the incidence of work-related stress (Cooper and Cartwright 1997). Secondary level interventions focus on the detection and management of experienced stress, and the enhancement of workers’ ability to effectively manage stressful situations and/or conditions. Tertiary level initiatives seek to minimise the effects that result from exposure to psychosocial hazards, through the management and treatment of symptoms of occupational disease or illness.

Hymel et al. (2011) observe that health protection and health promotion functions within an organization often act in silos. Hymel et al. (2011) propose a new concept, “Workplace Health Protection and Promotion”. This concept seeks to integrate these two previously separate functions (Hymel et al. 2011). So defined, “health promotion interventions contribute dynamically to improved personal safety in addition to enhancing personal health, while occupational safety interventions contribute dynamically to improved personal health in addition to enhancing personal safety”. Similarly, Tamers et al. (2019) propose the ‘Total Worker Health’ (TWH) framework, which is defined in relation to policies, programs, and practices that integrate protection from work-related safety and health hazards, with promotion of injury and illness prevention efforts to advance worker well-being. The National Institute for Occupational Safety and Health (2008) identifies twenty components of a comprehensive work-based health protection and health promotion program. The twenty components are divided into four areas: organizational culture and leadership; program design; program implementation and resources; and program evaluation.

## Human factors and culture 

### Performance shaping factors

The aviation human factors literature approaches the concept of wellbeing from a human performance and error management perspective. ‘Performance shaping factors’ include personal factors (i.e., training, fitness for work and stress), environmental factors and task-oriented factors that influence the probability of human error. Threat and error management (TEM) is an overarching safety concept regarding aviation operations and human performance (Skybrary 2021).

### Threat and error management

The threat and error management (TEM) framework highlights the interaction between people and the operational context (i.e., environmental, organisational, and regulatory factors) within which operators perform the tasks. In relation to flight operations, it defines three risk categories (i.e., crew, aircraft, and environment) which need to be considered as part of addressing safe performance and risk (Merritt and james Klinect 2006). As such, risk involves the assessment and management of the collective state of the crew, the aircraft, and the environment. Wellbeing is a key dimension of the crew state. Accordingly, the crew state can be conceptualized as a protective factor for safe performance (Cahill 2010, 2011).

### Culture

Organisational culture is often described as "the way we do things around here" (Guldenmund 2010). Ravasi and Schultz (2006) define organizational culture as a set of shared assumptions that guide the behaviors of an organisation’s members (for example, company employees). Organisational culture sets the boundaries for accepted operational performance in the workplace by establishing ‘norms and limits’ (ICAO doc 9859). Further, it influences the way in which staff share information and knowledge. This includes staff assumptions and beliefs about what information is safe to share. In this respect, Westrum (2004) details a taxonomy of organisational types and associated information cultures. This includes pathological organisations which hide information, bureaucratic organisations which restrain information and generative organisations which value information (Westrum 2004).

Safety culture is treated as part of organisational culture—reflecting the beliefs and values that employees share about how risks are managed within the organisation. Reason (1998) argues that this is associated with five elements. This includes (1) an informed culture, (2) a reporting culture, (3) a learning culture, (4) a just culture and (5) a flexible culture. As highlighted by the International Civil Aviation Organisation (ICAO), organisational culture shapes safety reporting procedures and practices by operational personnel (ICAO doc 9859).

In aviation, reporting practices are supported by a ‘just culture’, which enables the reporting of problems, without the fear of punitive consequences (Dekker 2018). The concept of ‘psychological safety’ (Kahn 1990; Edmondson 1999; Clark 2020), and ‘psychological safety climate’ (Dollard and Bakker 2010) is associated with ‘just culture’, and the management of psychosocial risk in the workplace. Kahn (1990) defines ‘psychological safety’ as ‘being able to show and employ oneself, without fear of negative consequences of self-image, status or career’. Psychological Safety Climate refers to ‘the shared perceptions regarding policies, practices and procedures for the protection of worker psychological health and safety” (Dollard and Bakker 2010). EU Regulations on data protection and embedded just culture clauses, have helped make front line staff more comfortable with occurrence reporting. Further, they have helped organisations and regulators become more aware of the need to ensure the investigation of occurrence reports is focused on improving systemic safety rather than taking punitive measures against reporters. Nonetheless, just culture provisions do not prevent organisations and regulators from taking actions against individuals in the case of wilful misconduct or reckless behaviour.

## Aviation context—managing wellbeing and mental health (MH)

### Short-term/operational aspects

Presently, the primary focus of airline wellbeing interventions is on management of crew fatigue and alertness. Fatigue risk is managed as part of the airline’s safety management systems (SMS’s). Typically, this involves a science based and operationally oriented fatigue management process in line with the ‘Fatigue Management Guide for Airline Operators’ specified by the International Air Transport Association (IATA), the International Civil Aviation Organisation (ICAO) and the International Federation of Airline Pilots Associations (IFALPA) (2015). Airline FRMS provide feedback to flight operations (including flight planning and crew rostering), to ensure that risks pertaining to fatigue are managed from an operational perspective. Fatigue is only one dimension of the biological pillar of wellbeing. As such, these systems and the wider SMS overlooks risks associated with the three pillars of wellbeing (Cahill et al. 2019).

Wellbeing has been embedded in many airlines training programs (i.e., health promotion, stress management training), in line with modifications to the existing crew resource management syllabus (EASA 2019). However, this training is not operationalized. Existing airline briefing processes (linking to CRM/TEM constructs) do not address WRS/wellbeing issues. Moreover, specific pre-flight checklists (i.e., standard operating procedures—SOP) do not include human factors checks in relation to crew wellbeing and the joint crew state (Cahill 2010, 2011).

Operational reporting is a key component of any SMS (Cahill 2010). Existing occurrence reporting systems do not explicitly focus on collecting information about staff wellbeing and mental health. However, nothing prevents staff from reporting wellbeing/WRS issues using these systems. Issues can also be reported to line managers. Anecdotally it is known that this is rarely done, given worker concerns about stigma (perception of colleagues) and the potential impact on employment (i.e., loss of income, job progression and job security). In the absence of formal wellbeing reporting policies and processes which provide protection to staff, reporting depends on trust. This trust cannot be assumed. Evidently, there is a potential for conflict in terms of protecting safety and protecting one’s own interest (i.e., stigma, financial implications of being off work, and career implications/loss of licence).

### Organisational approaches

Pilot health and fitness (including mental health) is assessed annually in accordance with mandatory rules regarding aero-medical assessment (Bor and Hubbard 2016). There are very clear guidelines concerning the impact of a psychiatric disorder on pilots and by implication flight safety (Dickens 2016). Accordingly, conditions that mean mandatory exclusion from flying and those that allow a pilot to fly under controlled conditions are distinguished (Dickens 2016).

In line with regulation, organisations also have a role in terms of providing wellbeing supports. This pertains to peer support programs for pilots. Feedback from recent industry events pertaining to MH (for example, annual meetings on MH in aviation at RaeS conferences during period 2019–2021) indicates that PSS are being implemented. However, information gathered via peer support channels is protected. Typically, high level and deidentified trend information is shared with the organisation, enabling the organisation to assess contributory factors to wellbeing problems. For example, some airlines such as British Airways collect anonymous data from peer support referrals to target organisational interventions (Phoenix 2021). This data provides an indication of the types of challenges that need to be addressed for employees. However, this does not mean the organisation obtaining a complete picture as regards the wellbeing of all employees and/or those who are suffering (i.e., peer support not used by all staff and/or all staff with wellbeing problems). In addition, many pilot unions have Pilot Advisory Groups (PAG). These provide a confidential service that pilots can use to talk about problems and obtain support.

In addition, aviation organisations provide employee assistance programs (EAP). There is considerable variability across aviation organisations in terms of the services provided and contractual arrangements (i.e., in house versus outsourced services).

## Methods

### Data collection

An anonymous online survey using the Qualtrics Survey Platform, was administered at two different time periods to investigate the impact of the COVID 19 pandemic on (1) job and employment, (2) wellbeing and morale, (3) performance and safety behaviour, and (4) safety oversight. The survey also investigated reporting culture, coping strategies, fitness to work assessment, and the supports provided by aviation companies to workers during the pandemic. Survey 1 was administered over 3 weeks during July and August 2020. Survey 2 was administered over 2 months (from October 2021 to early December 2021).

The survey incorporated several standardised instruments to measure levels of common mental health issues which have been widely validated and have good psychometric properties. These are Patient Health Questionnaire-9 (PHQ-9) (Kroenke et al. 2001) and the GAD 7 (Spitzer et al. 2006). The survey design drew upon prior research undertaken by the authors pertaining to a biopsychosocial model of wellbeing, the factors that can positively and negatively influence a pilot’s physical, mental, and social health, and the ensuing impact on pilot performance and flight safety (Cahill et al. 2018; Cullen et al. 2021). Participants were recruited using social media platforms such as LinkedIn and Twitter. Appendix 1 provides further information about survey topics and measures.

Key industry stakeholders were involved in validating the survey design. This includes representatives from the Flight Safety Foundation, The Royal Aeronautical Society and from industry working groups. This research was conducted in accordance with the Declaration of Helsinki, and the survey protocol was approved by the Ethics Committee of the School of Psychology, Trinity College Dublin (TCD) Ireland. The data protection impact assessment was approved by the Data Protection Officer at TCD.

### Data analysis, both surveys

Survey results were downloaded from the Qualtrics Platform in excel format. In relation to all survey questions, respondent feedback was recorded in relation to (1) all roles, and (2) the four most prevalent aviation roles, based on the profiles of survey respondents. These were pilots, cabin crew, maintenance/engineering and air traffic control (ATC). Appendix 2 provides an overview of respondent profile information for both surveys.

The PHQ 9 was scored in R (a programming language/tool for statistical computing), using validated R code (Scripts and Statistics 2021). The GAD 7 was scored in excel. Appendix 3 provides further information on the cut-offs and scoring methods for the both the PHQ 9 and GAD 7. All descriptive statistics were obtained using R. This includes measures of central tendency for all wellbeing scores (i.e., life satisfaction and happiness, depression, anxiety, and suicidal ideation) for all workers and for the four highest groups in relation to aviation roles.

Respondents were invited to provide example of company supports, where relevant. The text information was analysed using excel. First, the text feedback was cleaned. Specific examples were coded into three high level categories (primary, secondary, and tertiary), and subcategories. Counts were undertaken for each subcategory. Appendix 10 provides a % breakdown of these counts.

### Additional data analysis (regression analysis), 2021 survey

Additional analysis was undertaken in relation to the 2021 survey findings. Thirty-six simple linear regression models were advanced in R to evaluate whether a statistically significant relationship exists between different independent/predictor variables (*X*) and dependent/outcome variables (*Y*). Each model included one predictor variable and one outcome variable.

Overall, twelve categorical predictor variable/*X* were selected. These were

1. Spoken MH and scores.
2. Airline provided supports and scores.
3. Company Cares about wellbeing and scores.
4. Would look for help if had MH issue and MH Scores.
5. Willingly disclose to employer.
6. Using coping strategies and scores.
7. Use of company supports and scores.
8. Use of outside supports and scores.
9. Changes in morale and impact on safety practices.
10. Competence and ability to do the job safely.
11. Compliances with safety procedures and practices.
12. Motivation towards the job.

Dummy variables (1, 0) were implemented for the single predictor variable used in each model. Appendix 5 provides an overview of the logic for the dummy variable, for each model. For each predictor variable, three different health outcomes (*Y*) were examined. The health outcomes corresponded to the calculation of the total numeric scores for the three wellbeing measures evaluated in the survey (i.e., depression, life satisfaction and happiness, and anxiety). Predictors of suicidal ideation were not evaluated. Only those observations which included responses for both *X* and *Y* were included in the analysis. Table 1 provides an example of the model structure, for one example predictor/*X* variable (i.e., talked to somebody), and one example outcome variable (i.e., depression).

**Table 1:** Model structure

Y(Depression) = β0 + β1 (talked to somebody) + e	

Appendix 11 provides a summary of the high-level test results for each model. Appendix 12 provides an overview of the specific binary regression model findings, including *R*^2^, regression coefficients, *p* values and confidence intervals.

## Results

### Introduction

The research findings are structured into two parts. The first part provides a summary of survey findings in terms of nine core themes and subthemes (see Table 2). The second part addresses the results of the preliminary regression analysis.

**Table 2:** Themes and survey

#	High-level theme	#	Sub-theme
1	Health and wellbeing	1.1	Self-reported physical health
		1.2	Self-reported mental health
		1.3	Impact of COVID on mental health
		1.4	Levels of wellbeing (happiness and life satisfaction)
		1.5	Levels of wellbeing (depression)
		1.6	Levels of wellbeing (suicidal ideation)
		1.7	Levels of wellbeing (anxiety)
		1.8	COVID 19 Pandemic and impact on family
		1.9	Job loss, changes in job and financial wellbeing
2	Talking about MH and seeking help		
3	Coping methods/self-care and seeking help		
4	COVID and impact on performance and safety		
5	COVID and impact on engagement and motivation		
6	Wellbeing, organisational priorities and wellbeing culture		
7	Wellbeing supports provided by company during pandemic and use		
8	COVID 19 pandemic and return to work		
9	Safety oversight		

### Theme 1: health and wellbeing

#### Physical health

**Fig. 1:**
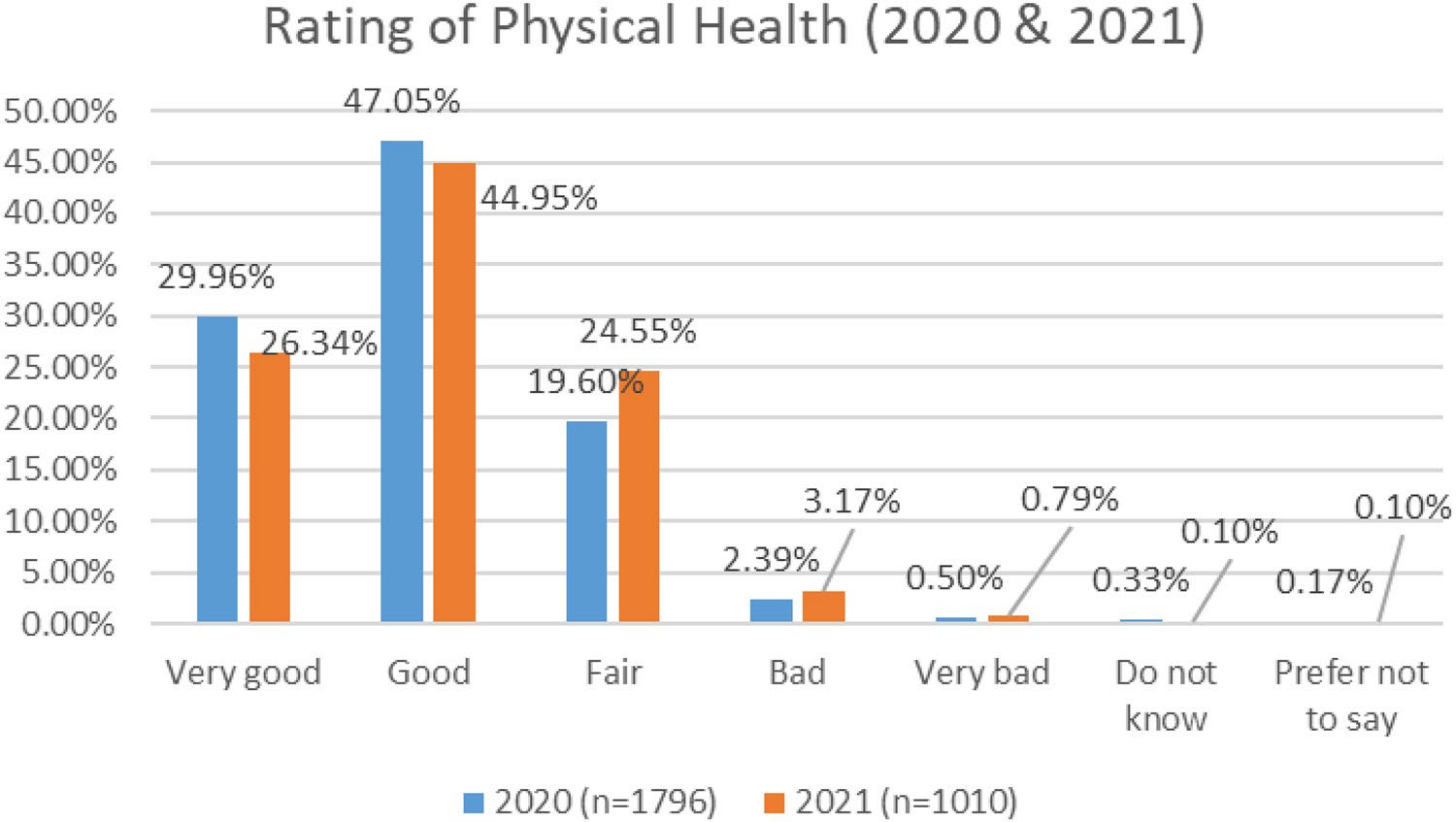
Comparison of physical health, 2020 and 2021 surveys

As indicated in Fig. 1, in the 2020 COVID survey, 77% of respondents rated their physical health as good or very good, while in the COVID 2021 survey, only 71% rated their physical health as good or very good.

**Fig. 2:**
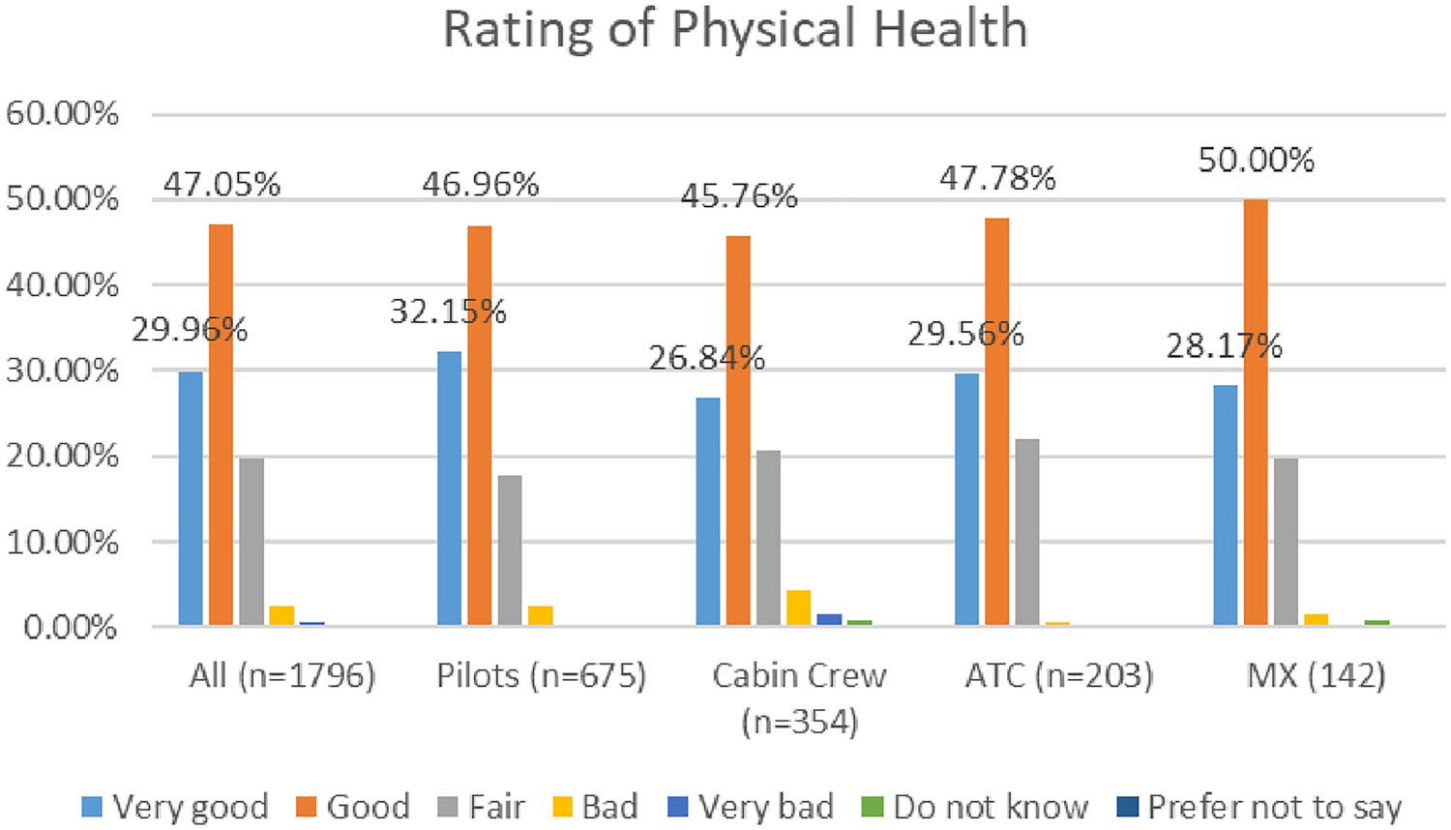
Physical health, roles breakdown, 2020

**Fig. 3:**
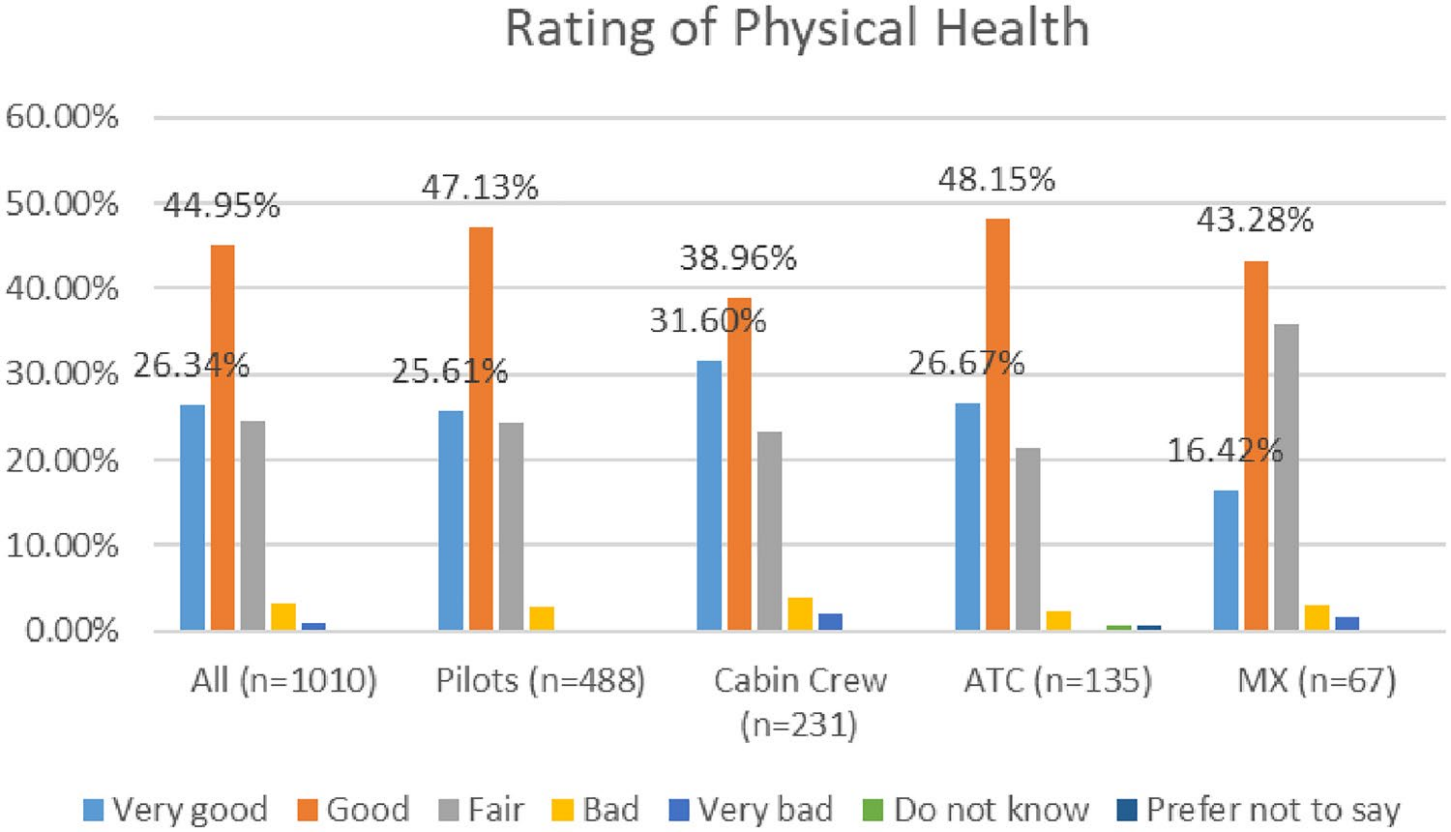
Physical health, roles breakdown, 2021

In the 2020 survey, cabin crew reported lower levels of positive physical health compared to other workers (73%), while in the 2021 survey, maintenance/engineering personnel reported the lowest levels of positive physical health (60%). See Figs. 2 and 3.

#### Mental health

**Fig. 4:**
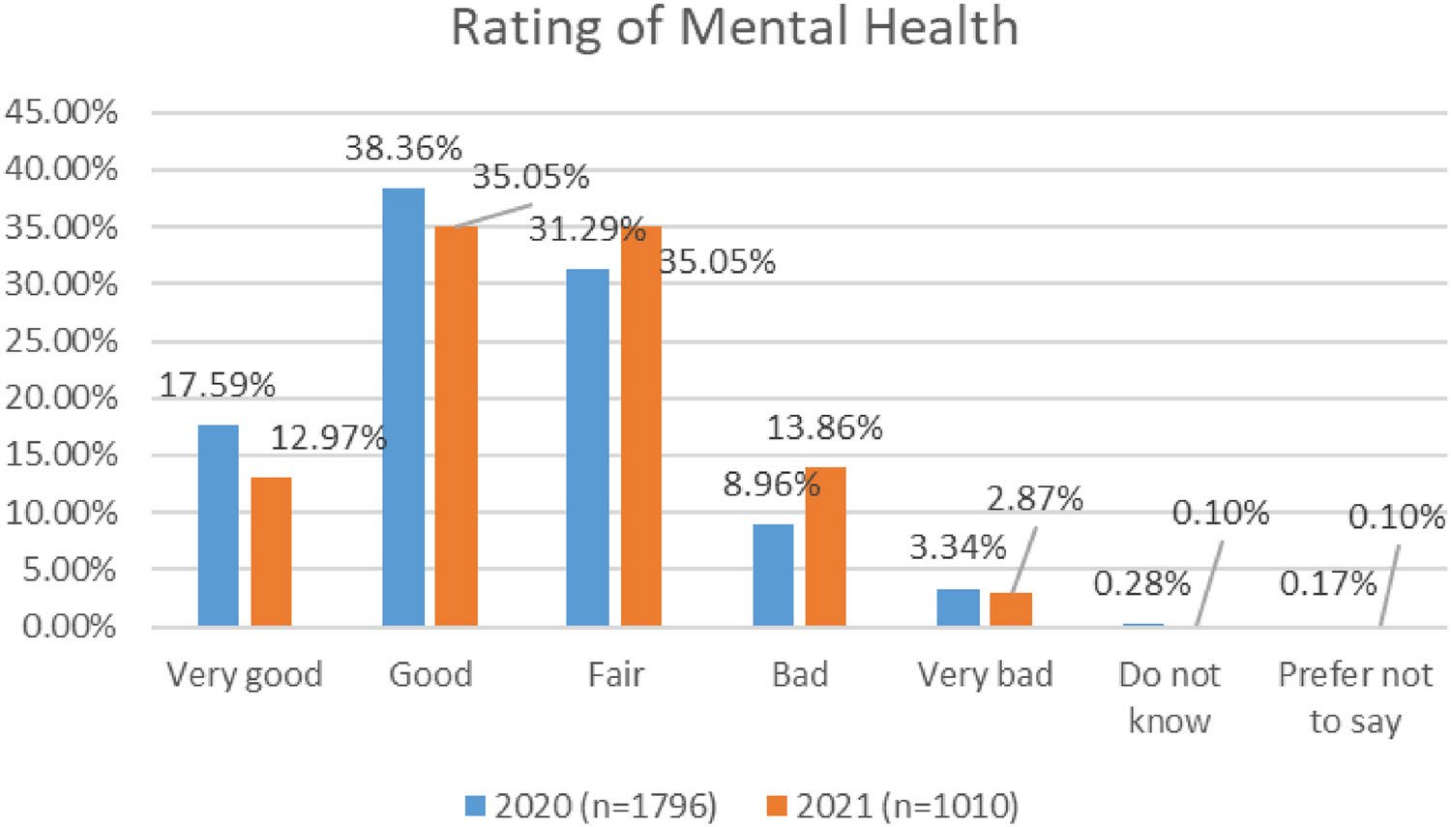
Comparison of mental health, 2020 and 2021 surveys

As depicted in Fig. 4, higher numbers of respondents (56%) reported their mental health as ‘good’ or ‘very good’ in the COVID 2020 study, as compared with the COVID 2021 study (48%).

**Fig. 5:**
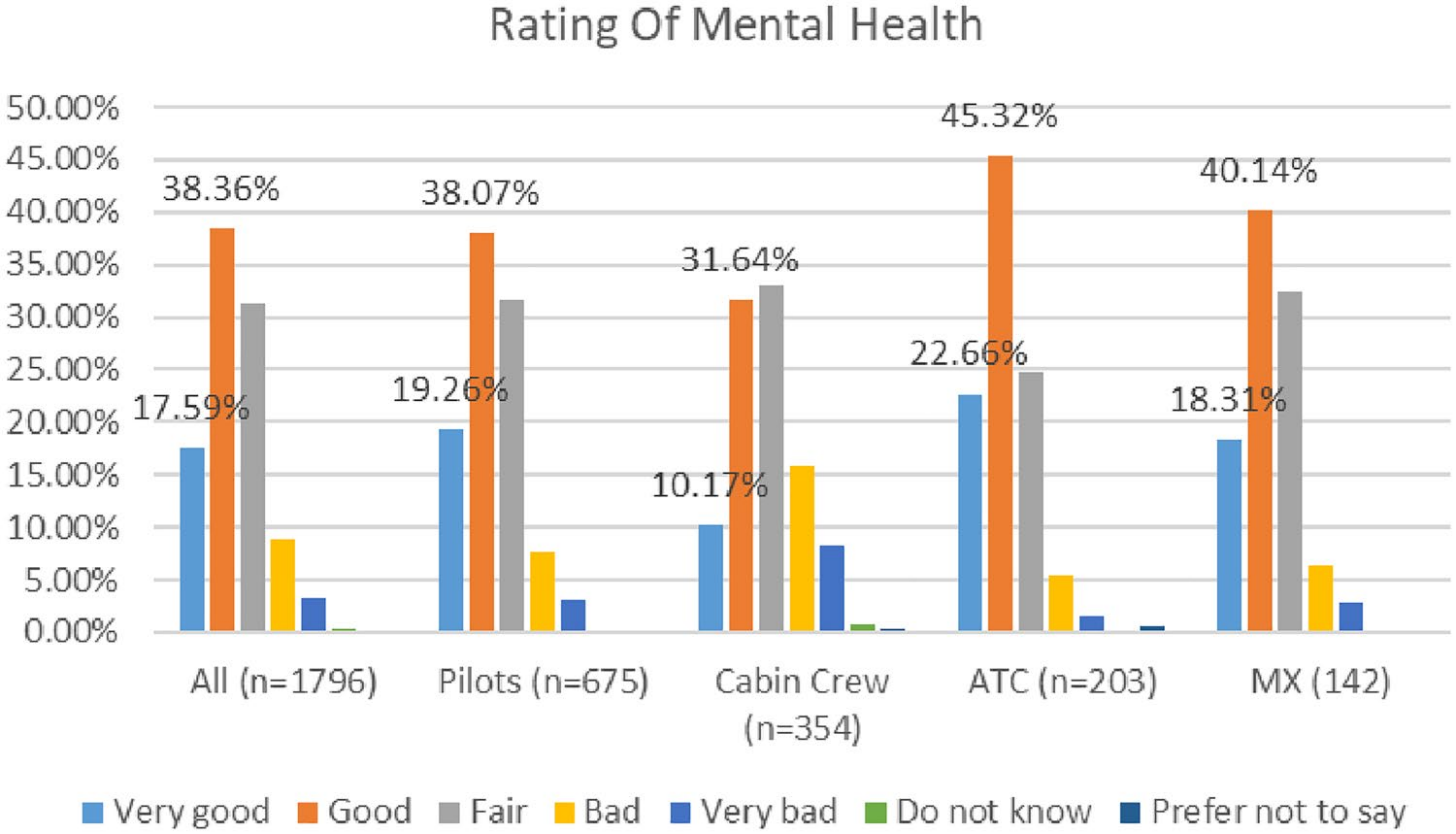
Mental health, roles breakdown, 2020

**Fig. 6:**
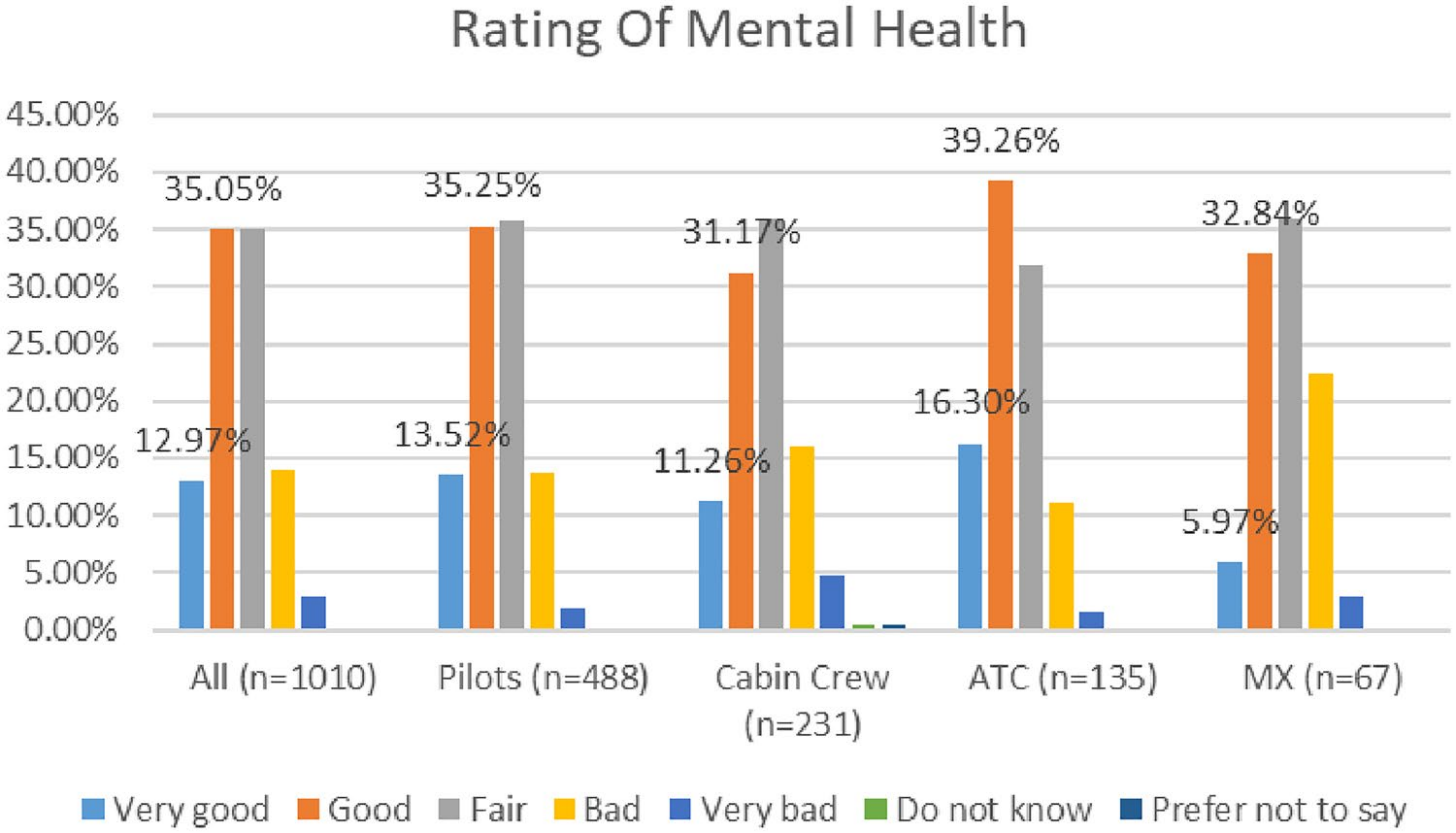
Mental health, roles breakdown, 2021

In the 2020 survey cabin crew reported the lowest levels of positive mental health (42%), while in the 2021 survey, maintenance/engineering reported the lowest levels of positive mental health (39%). See Figs. 5 and 6.

#### Negative impact of COVID on health and wellbeing

**Fig. 7:**
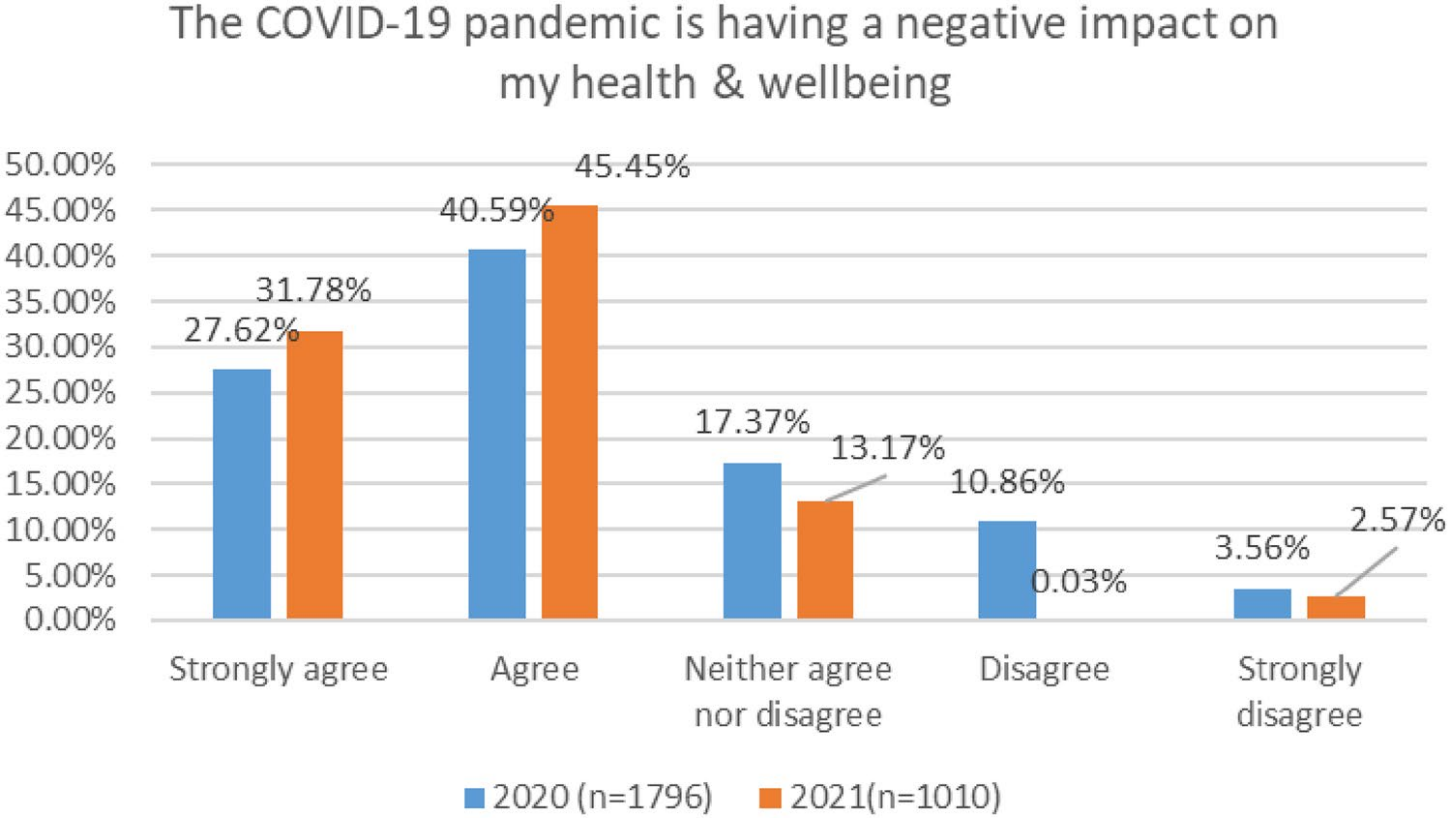
Comparison of impact of COVID pandemic on MH, 2020 and 2021 Surveys

As shown in Fig. 7, in 2020, 68% of respondents agreed or strongly agreed that the COVID 19 Pandemic is having a negative impact on their health and wellbeing, with the figure rising to 77% in 2021.

**Fig. 8:**
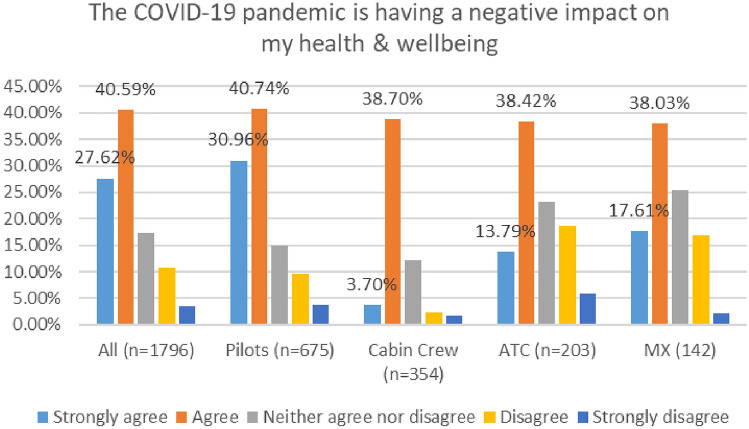
Impact of COVID on MH, roles breakdown, 2020

**Fig. 9:**
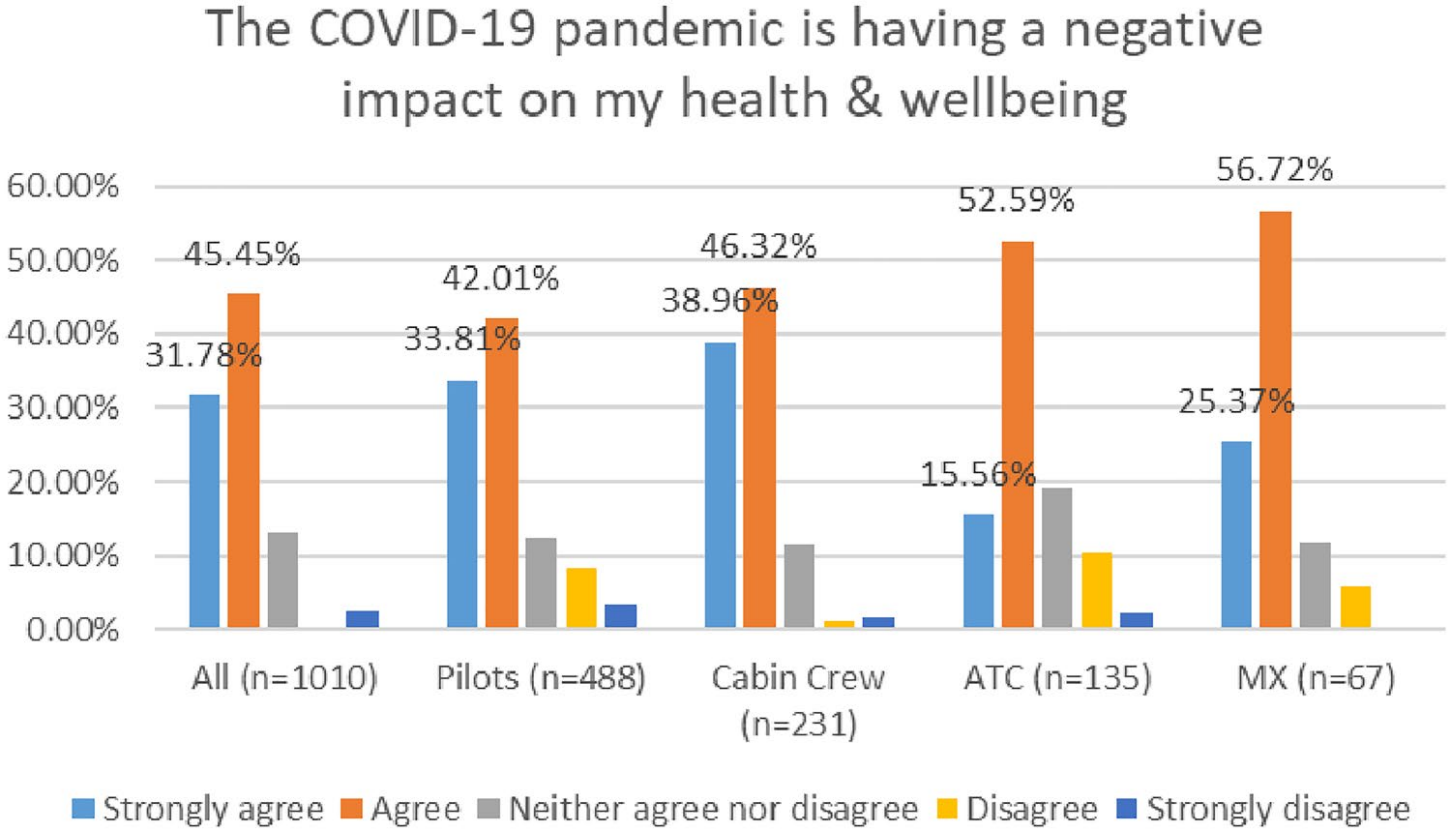
Impact of COVID on MH, roles breakdown, 2021

In the 2020 survey, pilots reported the highest level of agreement with this statement (i.e., negative impact of COVID 19 pandemic on my health and wellbeing)—72%. However, in the 2021 survey, maintenance/engineering reflected the highest levels of agreement—82% (Figs. 8, 9).

**Fig. 10:**
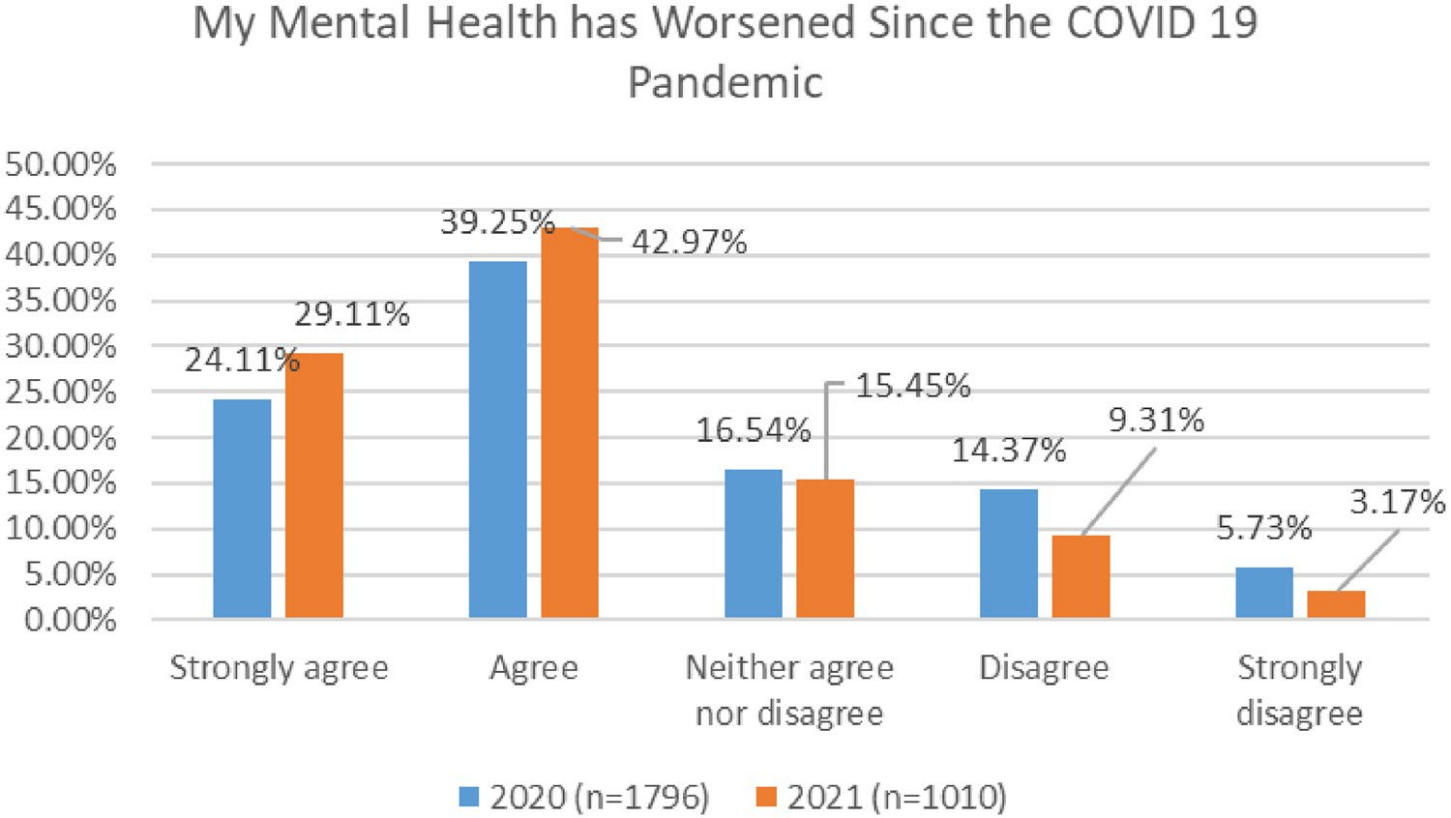
MH worsening since COVID—comparison of 2020 and 2021 surveys

Further, as indicated in Fig. 10, most respondents agreed that their mental health has worsened since the COVID 19 pandemic, with 63% agreeing or strongly agreeing in 2020 and 72% in 2021.

**Fig. 11:**
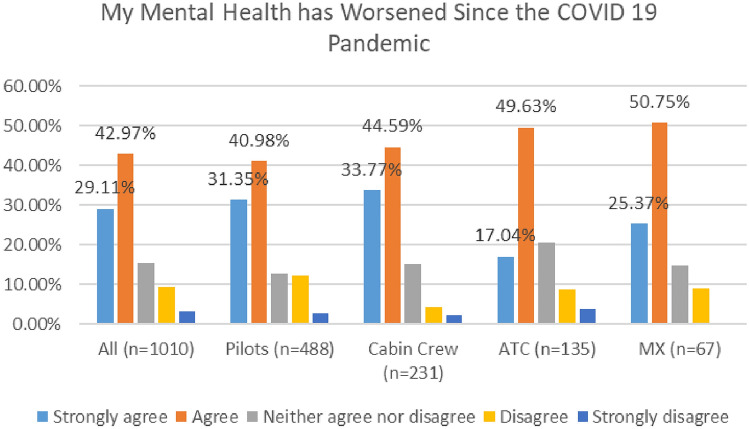
MH worsening since COVID roles breakdown, 2020

**Fig. 12:**
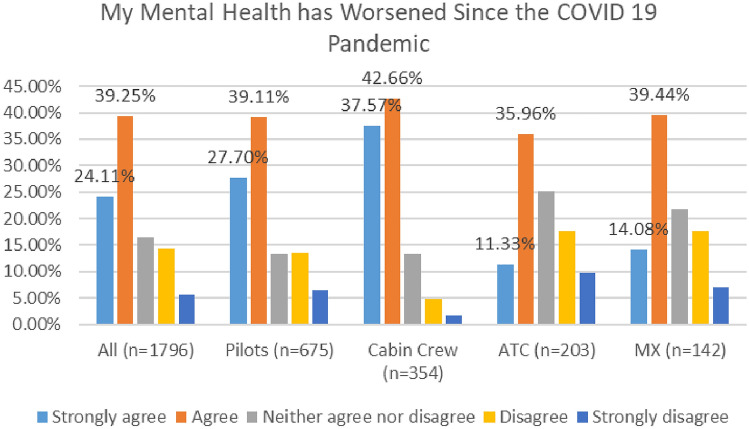
MH worsening since COVID roles breakdown, 2021

In both surveys, cabin crew reported the highest level of agreement with this statement, with 78% agreeing or strongly agreeing in 2020, and rising to 80% in 2021 (Figs. 11, 12).

#### Levels of wellbeing (happiness and life satisfaction)

**Fig. 13:**
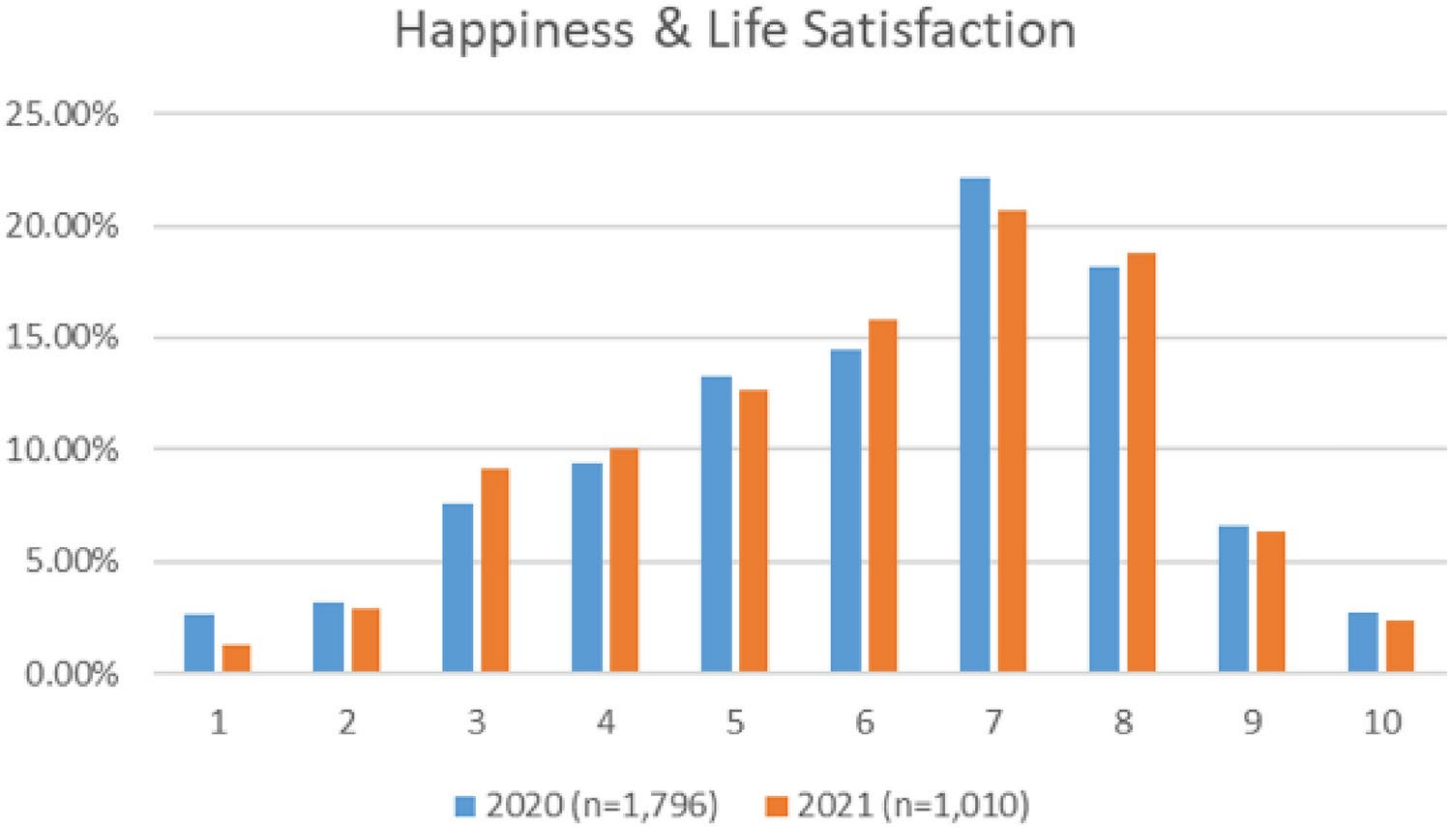
Comparison of happiness and life satisfaction, 2020 and 2021 surveys

As depictured in Fig. 13, the mean scores for happiness and life satisfaction were similar across both the 2020 and 2021 survey (6.086 in 2020 and 6.089 in 2021), although there were some differences between the distribution of scores across both surveys. The median score was the same in both surveys (both 6). For specific breakdowns, please see Appendix 6.

The mean and median happiness and life satisfaction scores for cabin crew was lower than pilots, ATC, and maintenance/engineering personnel, in both surveys. For specific breakdowns, please see Appendix 4.

#### Depression

**Fig. 14:**
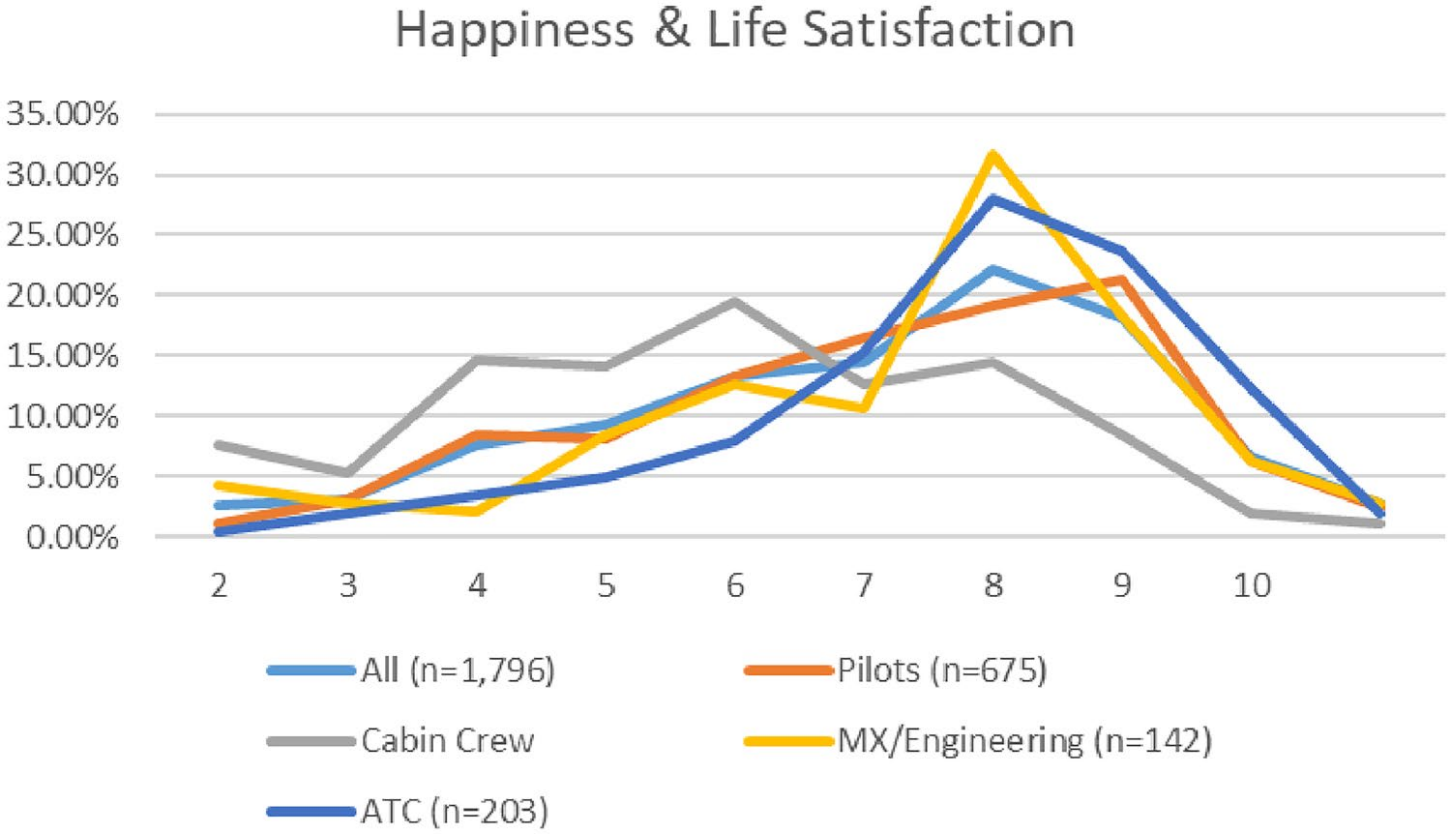
Happiness and life satisfaction, roles breakdown, 2020

**Fig. 15:**
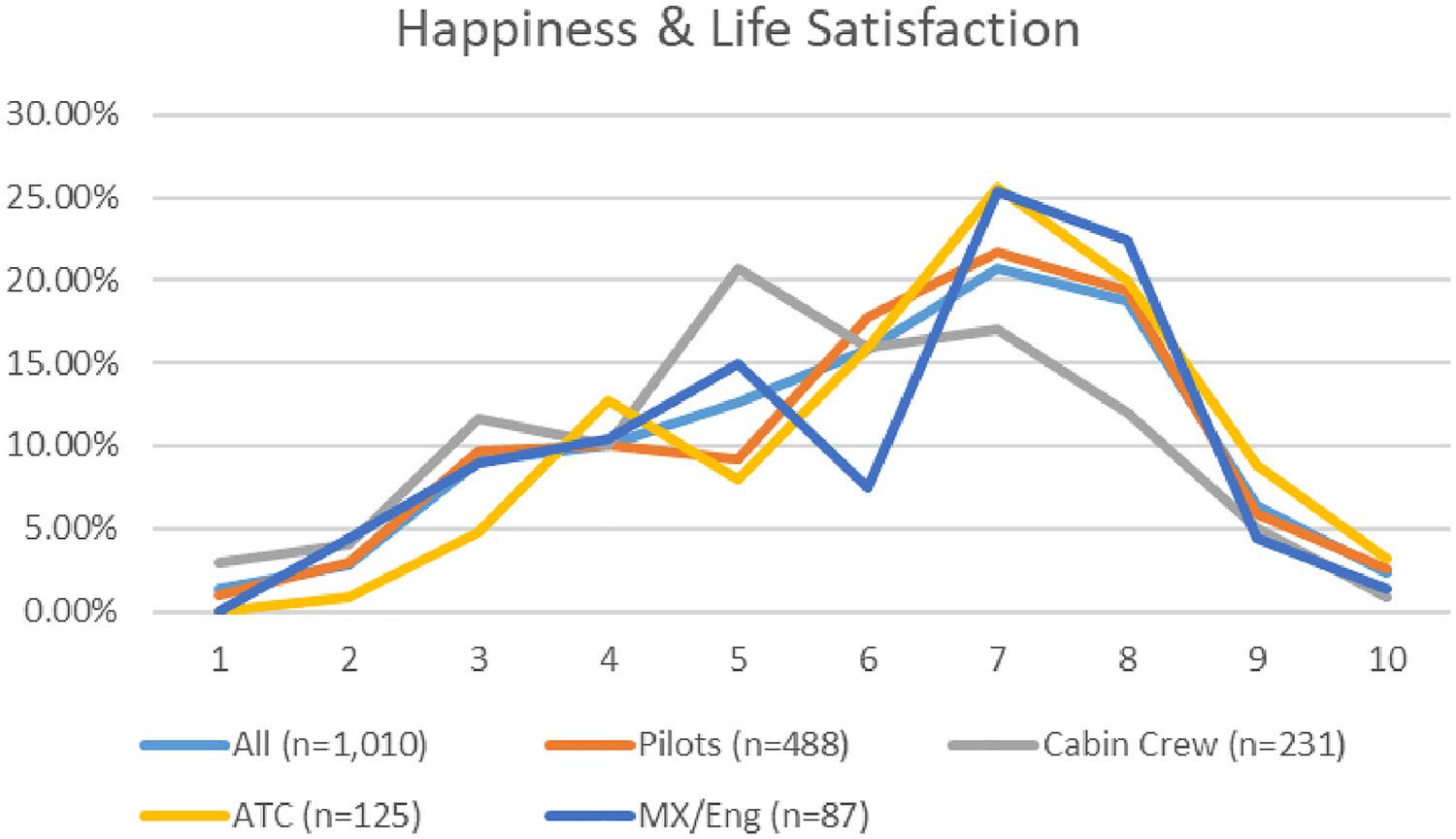
Happiness and life satisfaction, roles breakdown, 2021

**Fig. 16:**
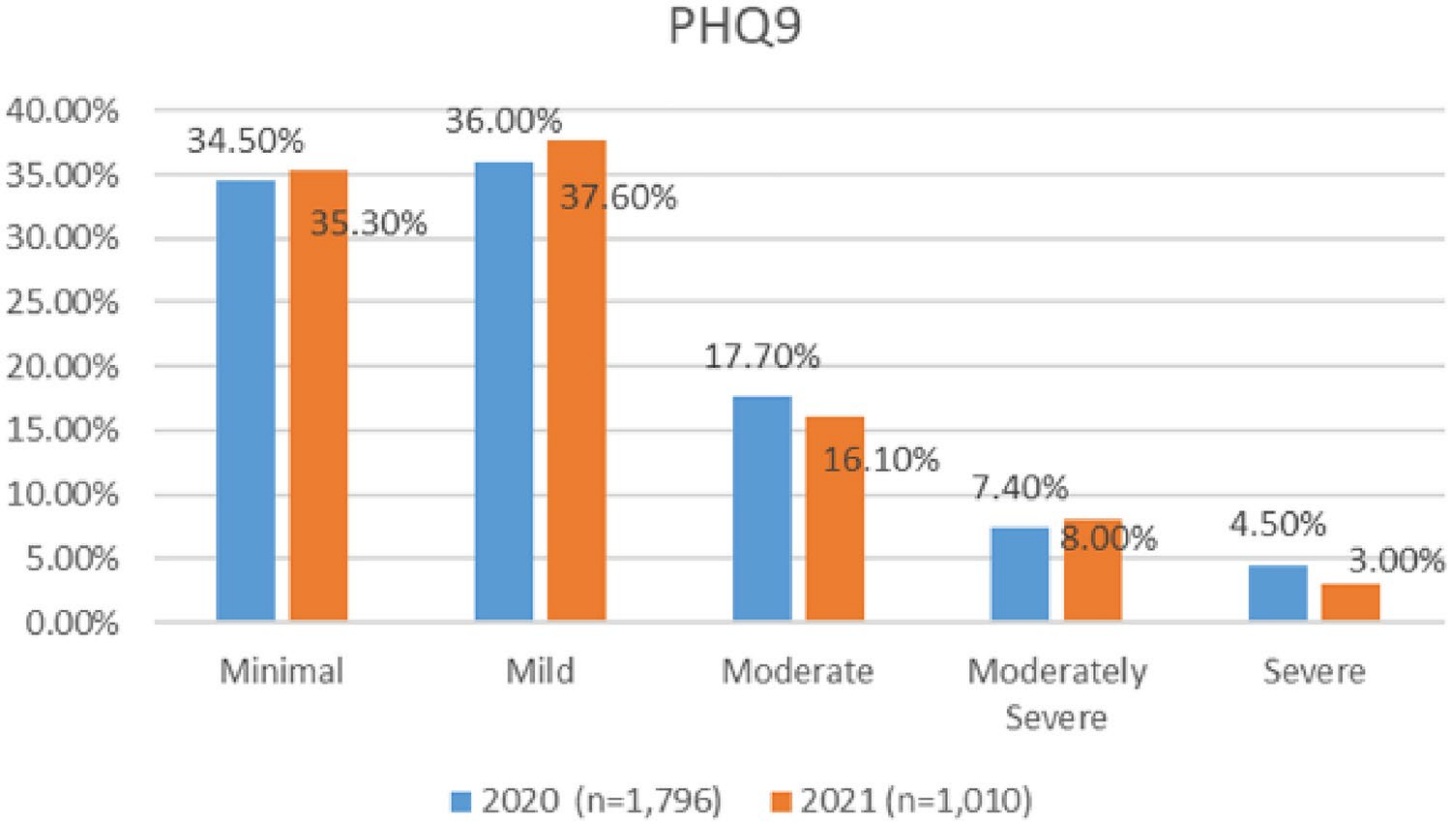
Comparison of depression severity scores, 2020 and 2021 Surveys

A comparison of the depression severity levels reported in the two surveys (see Fig. 14), indicates an overall improvement in depression prevalence. The numbers of aviation workers screening on or above the threshold for moderate depression on the PHQ9 (i.e., combining moderate, moderately severe, and severe depression), dropped from 29.6% in 2021, to 27.1% in 2020 (Fig. 15). Lower numbers of aviation workers met the threshold for moderate depression (dropping from 17.7% in 2020 to 16.1% in 2021). However, there was an increase in numbers meeting the threshold for minimal and mild depression. In relation to numbers screening positive on the PHQ9 for minimal depression, there was a small increase (from 34.5 to 35.3%). In relation to mild depression, numbers increased from 36 to 37.6% (Fig. 16). Further, the numbers meeting the threshold for moderately severe depression increased during the COVID timeframe (7.4% in 2020 to 8% in 2021). However, the numbers meeting the threshold for severe depression decreased (4.5% in 2020 to 3% in 2021).

**Fig. 17:**
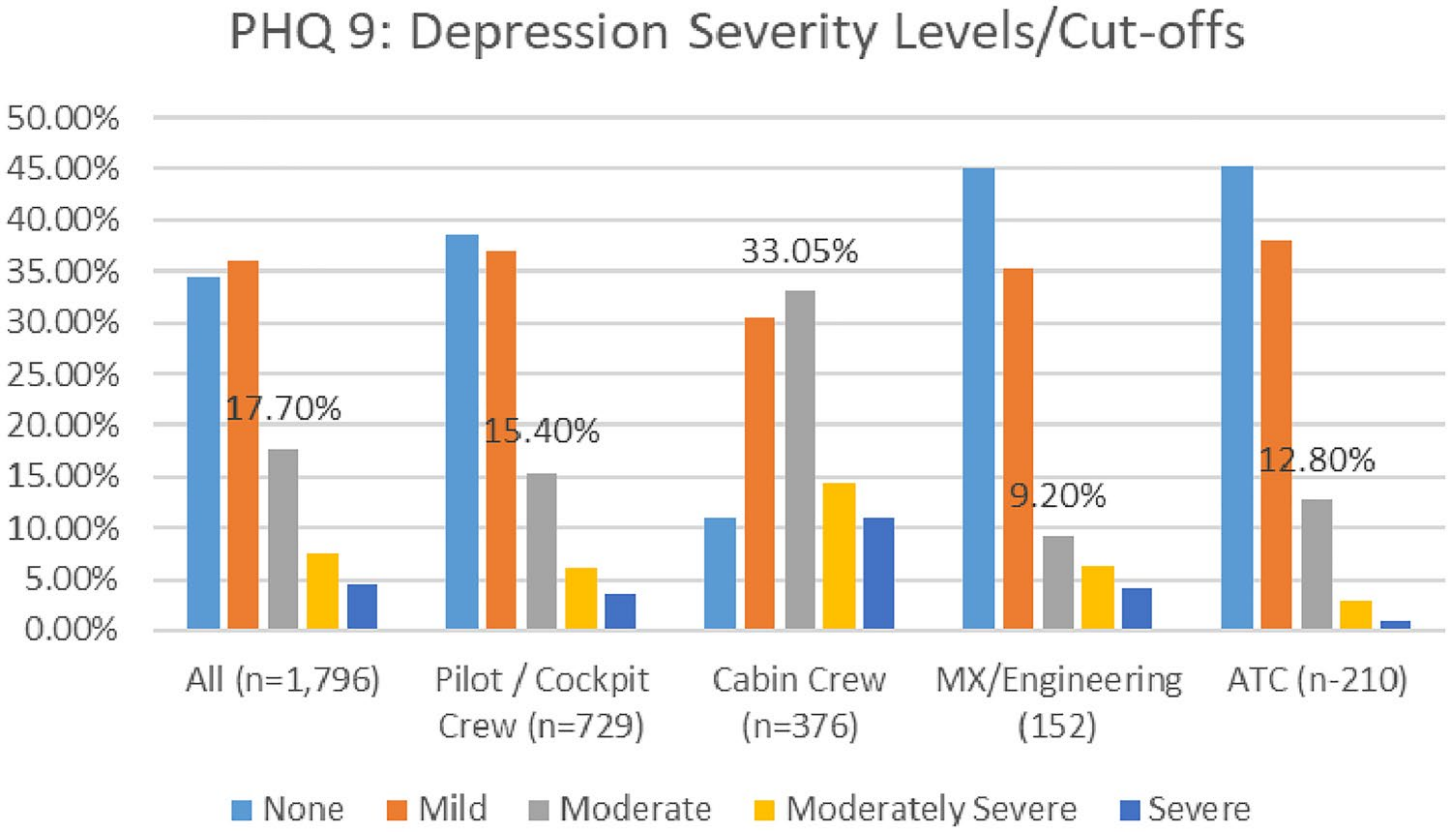
Depression severity, roles breakdown, 2020

**Fig. 18:**
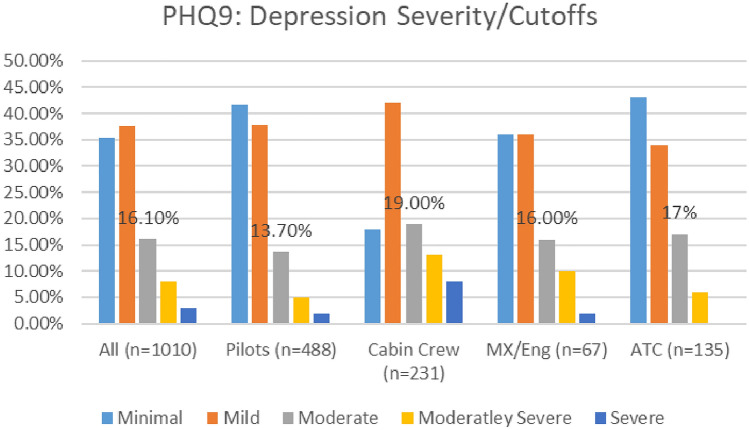
Depression severity, roles breakdown, 2021

In relation to roles, in both surveys, higher numbers of cabin crew met the threshold for moderate depression using the PHQ9, as compared with other worker profiles—33% in 2020 and 19% in 2021 (Figs. 17, 18).

#### Suicidal ideation

**Fig. 19:**
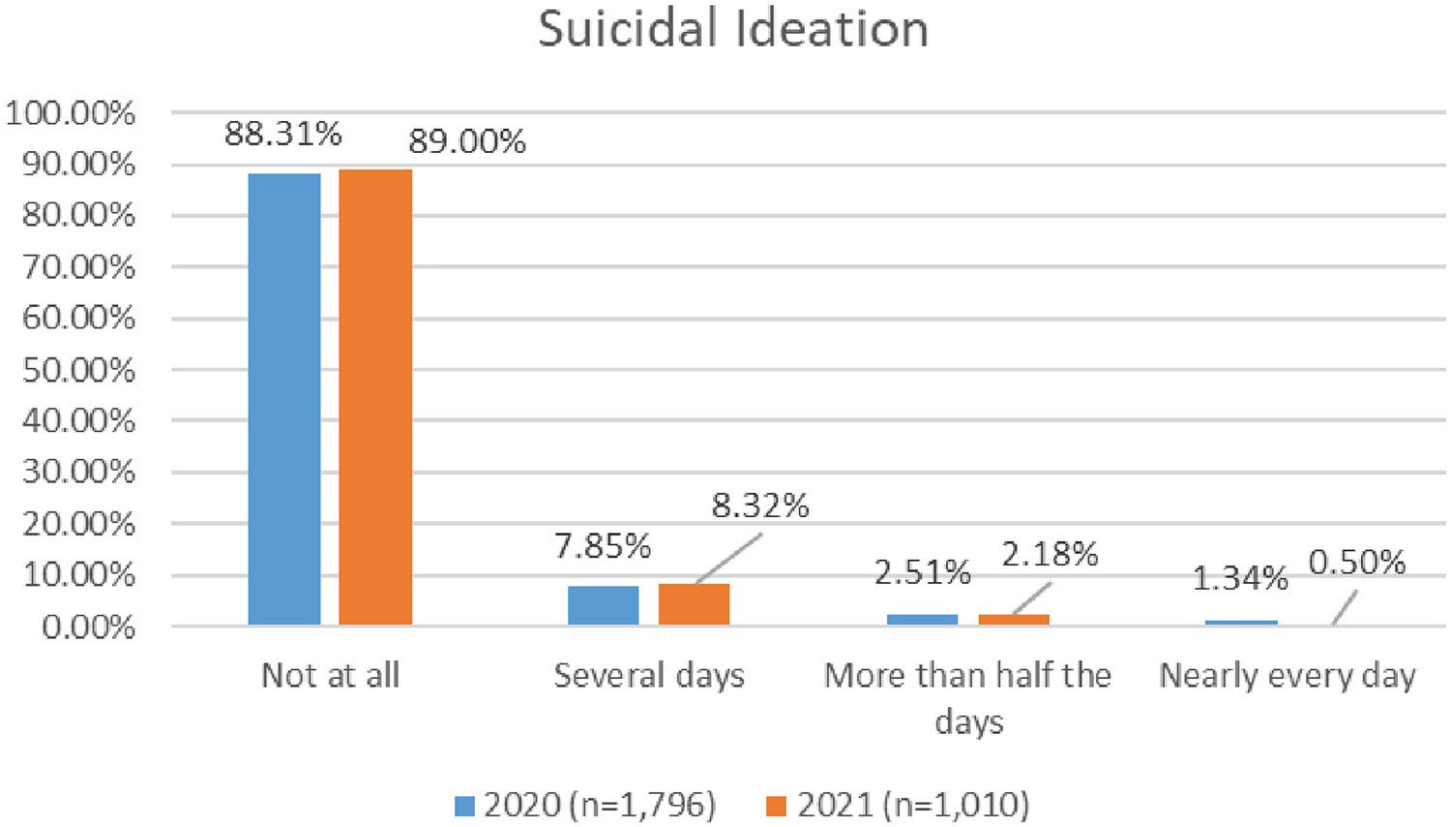
Comparison of suicidal ideation, 2020 and 2021 surveys

As shown in Fig. 19, there appears to be a marginally downward trend in the numbers of aviation workers screening positive for suicidal ideation using the PHQ 9 (moving from 11.69% in 2020 to 11% in 2021).

**Fig. 20:**
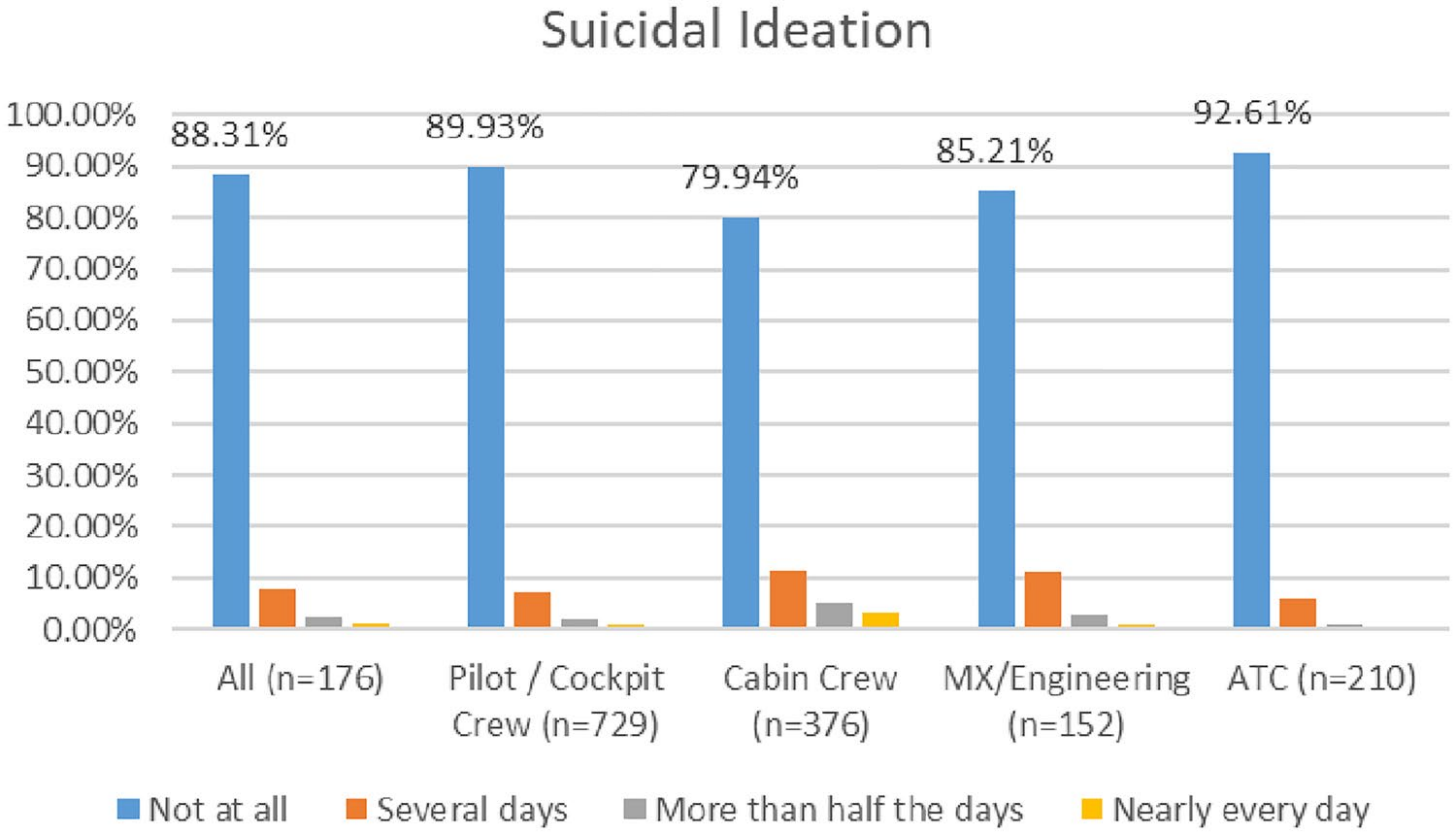
Suicidal ideation, roles breakdown, 2020

**Fig. 21:**
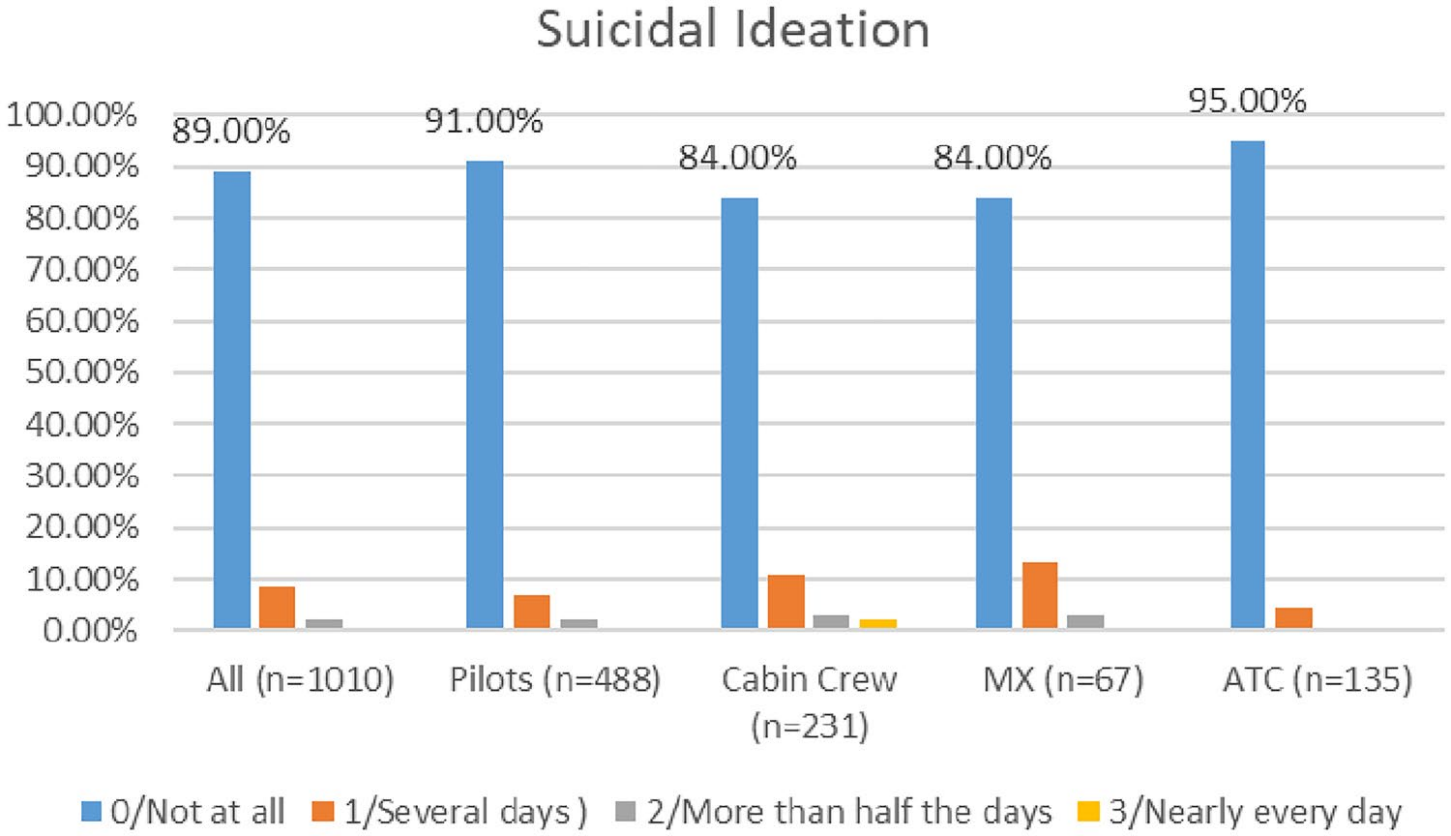
Suicidal ideation, roles breakdown, 2021

In the 2020 survey, cabin crew reported higher levels of suicidal ideation as compared with other groups—with 80% indicating ‘not at all’. In the 2021 survey, cabin and maintenance engineering reported higher levels of suicidal ideation—with 84% of respondents from both groups reporting ‘not at all’ (Figs. 20, 21).

#### Anxiety

**Fig. 22:**
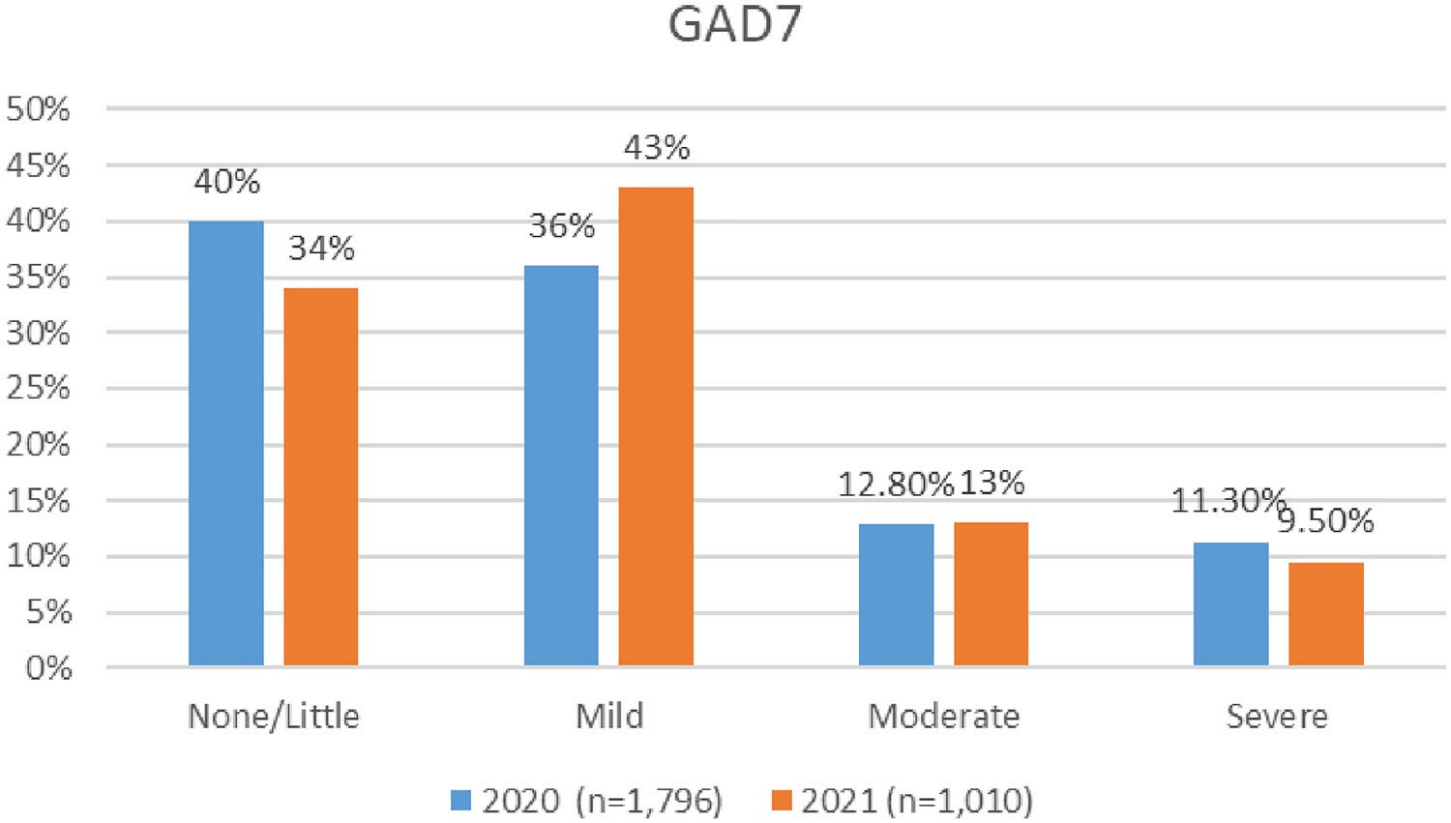
Comparison of anxiety scores, 2020 and 2021 surveys

Overall, levels of anxiety improved during the COVID timeframe. As indicated in Fig. 22, the numbers of aviation workers screening either on or above the threshold for moderate anxiety using the GAD 7 (i.e., combined figures for moderate and severe anxiety) dropped from 24.1% in 2020 to 22.5% in 2021. In the 2021 survey, there were marginally higher numbers meeting the threshold for moderate anxiety—13%, as compared to 12.80% in 2020. However, in 2021 survey, there was a reduction in the numbers meeting the threshold for severe anxiety (9.5% in 2021, as compared with 11.3% in 2020).

**Fig. 23:**
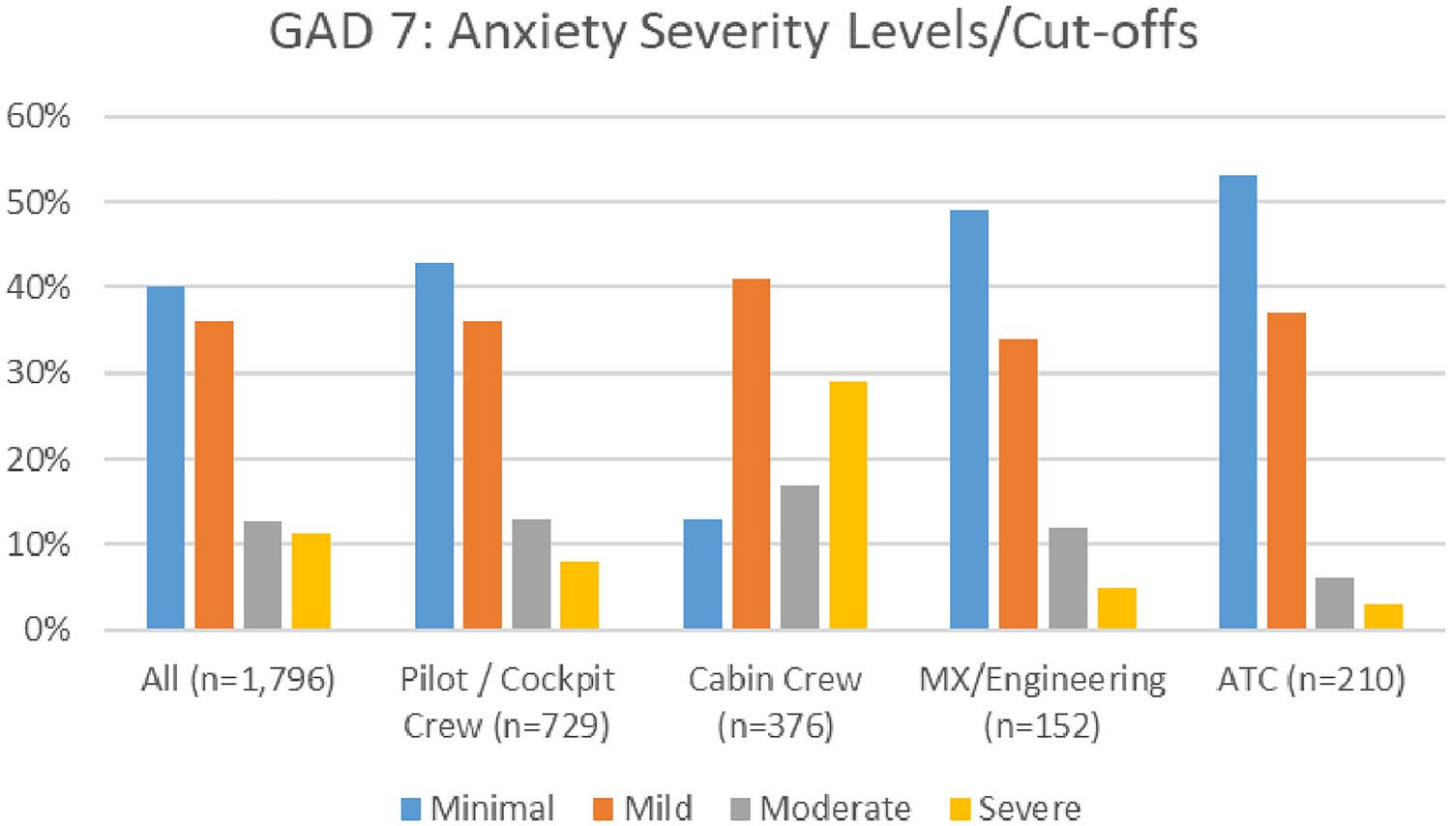
Anxiety scores and roles breakdown, 2020

**Fig. 24:**
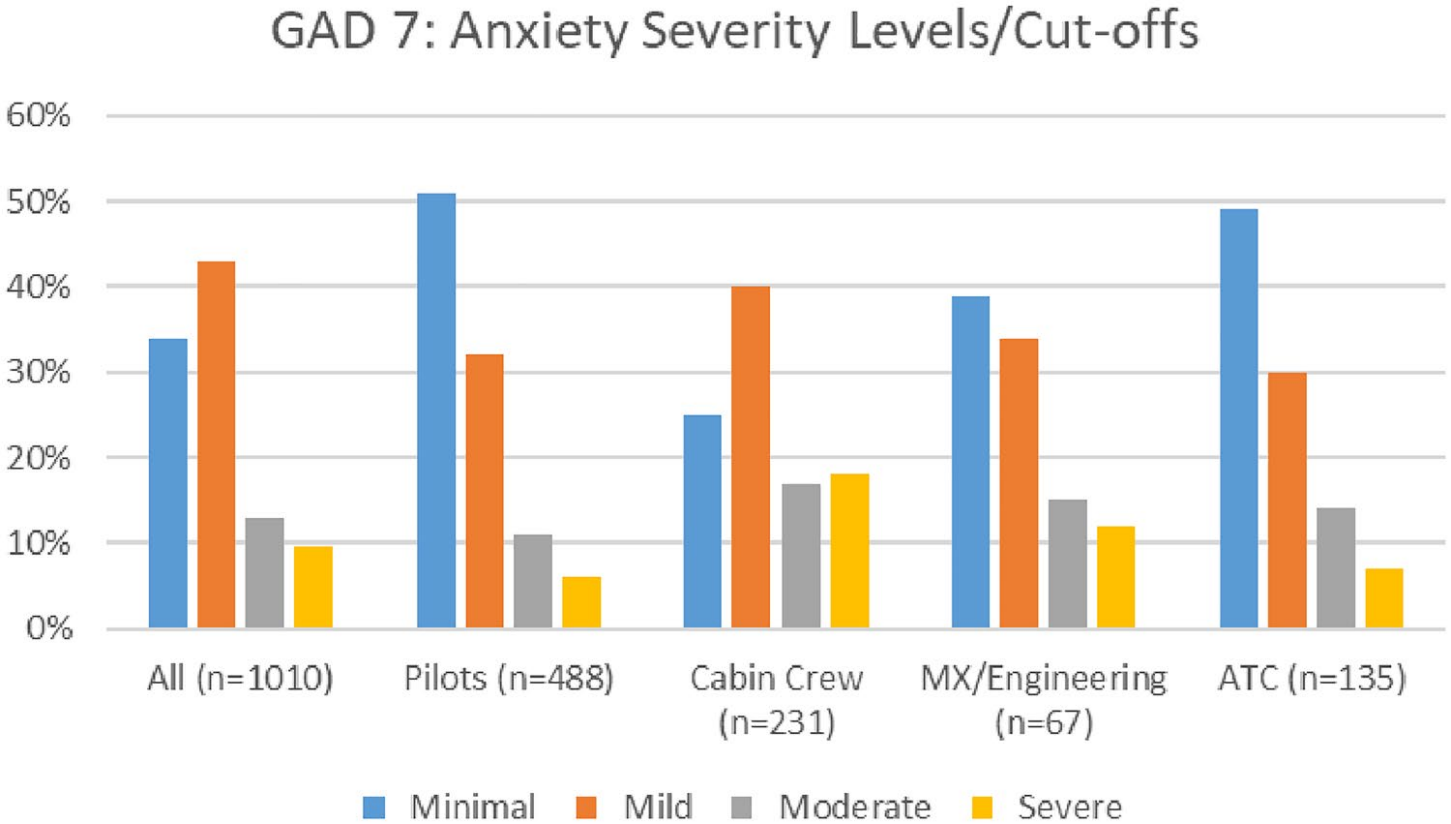
Anxiety scores and roles breakdown, 2021

Both surveys indicate that the prevalence of moderate and severe anxiety is highest in cabin crew, as compared with other roles (Figs. 23, 24).

#### COVID 19 pandemic and impact on family

In 2021, higher numbers of respondents reported that the wellbeing of their family has been negatively impacted by the challenges in their work situation (71% agreeing or strongly agreeing in 2021 and 57% in 2020).

#### Changes in job, job loss and financial wellbeing

In both surveys, approximately half of respondents indicated that their employment status has changed since the onset of the COVID-19 pandemic (50% in 2020 and 51% in 2021), with a large number having lost their job (51% in 2020 and 40% in 2020). Of those who reported losing their job, this job loss is permanent (41% in 2020 and 45% in 2021). Across both surveys, nearly two thirds of respondents reported worrying about meeting financial obligations (68% in 2020 and 64% in 2021). In both surveys, respondent feedback indicates strong levels of uncertainty about the future. In both numbers, small numbers reported feeling confident about their future employment in aviation (20% in 2020 and 24% in 2021).

### Theme 2: talking about MH and seeking help

Across both survey, aviation workers reported low levels of speaking out about mental health issues. In the 2020 survey, 67% of respondents agreed or strongly agreed that there are low levels of speaking out and/or reporting about mental health amongst their colleagues, while in the 2021 COVID study, 73% agreed or strongly agreed. In both surveys, similar numbers of respondents (approx. 34%) indicated that talking about MH happens less than once a month. A very low number of pilots and aviation workers reported that they would willingly disclose a mental health issue to their employer (22% in the 2020 survey, and 20% in the 2021 survey). Close to half of the participants in both surveys indicated that they had talked to somebody about a MH health issue they were experiencing/had experienced (54% in 2020, and 47% in 2021). In both surveys, respondents indicated that they would be more likely to approach a family or friend (23% approx. in both studies) and a medical professional (approx. 22% in both studies) about a mental health issue. Only a very small number of respondents reported talking to Peer Support about mental health problems (2 55% in the 2020 COVID survey and 3.58% in the 2021 survey).

### Theme 3: coping methods/self-care and seeking help

**Fig. 25:**
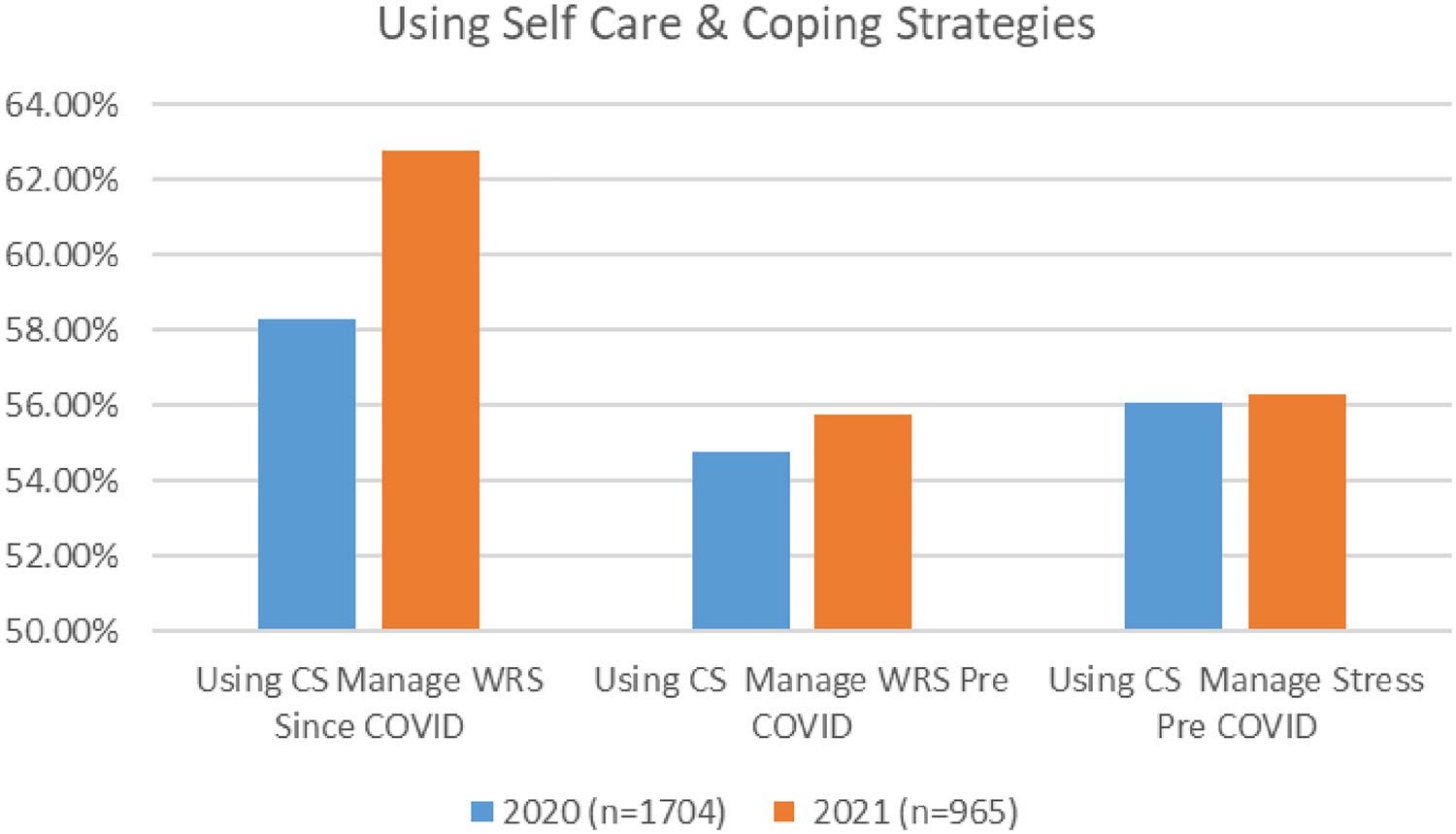
Comparison of use of coping strategies, 2020 and 2021 surveys

Survey results indicate that aviation workers are continuing to use coping strategies. As indicated in Fig. 25, both surveys demonstrate similar patterns in terms of the numbers of aviation workers using coping strategies to deal with both stress and work-related stress pre COVID, and since COVID (i.e., similar numbers), with marginally higher numbers using coping strategies in 2021. Over half of respondents in both surveys reported using self-care routines pre COVID to deal with stress (56.27% in 2020 and 56.10% in 2021) and WRS (54.75% in 2020 and 55.75% in 2021). Further, in both surveys, higher numbers of respondents reported using coping strategies to help them cope with stress and any changes to your wellbeing and mental health since the onset of the COVID-19 pandemic (58% in 2020 and 63% in 2021).

**Fig. 26:**
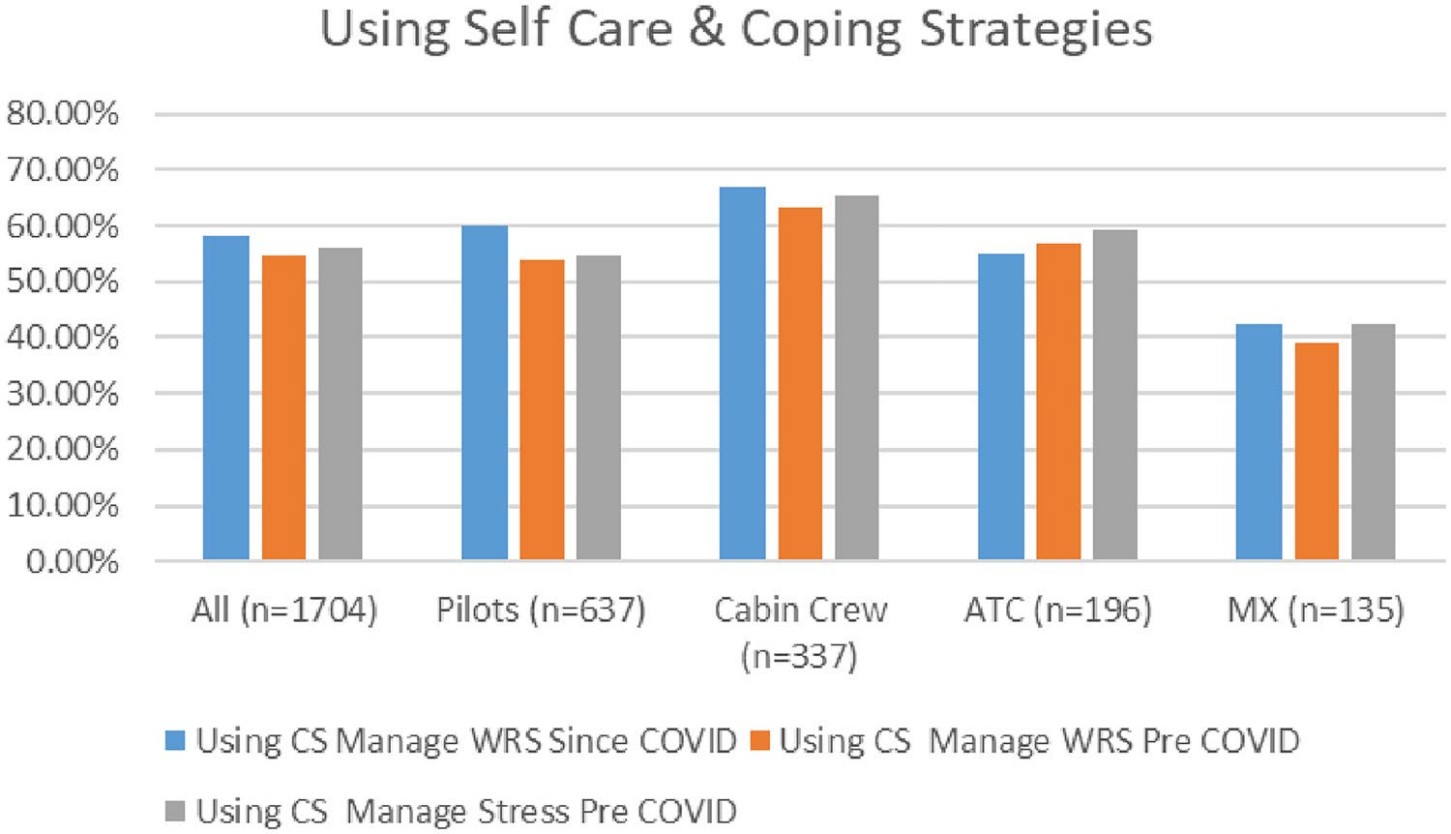
Use of coping strategies and roles breakdown, 2020

**Fig. 27:**
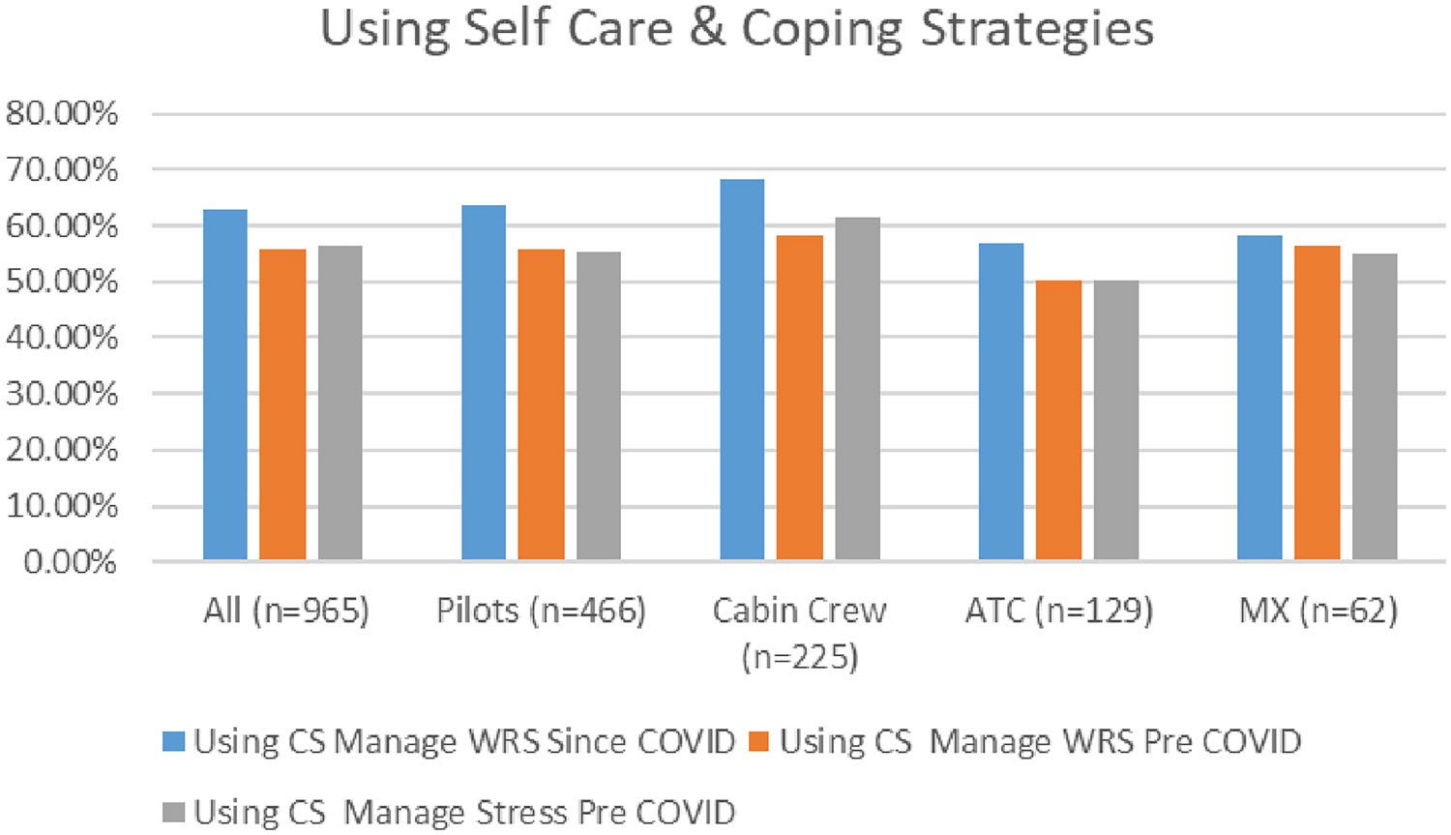
Use of coping strategies and roles breakdown, 2021

In both surveys, cabin crew reported the highest use of coping strategies—both pre and since the pandemic, with the use of coping strategies similarly distributed across all worker profiles (Figs. 26, 27).

In both surveys, a similarly high number of respondents reported that they would seek help if needed (68% in 2020 and 67% in 2021).

### Theme 4: impact on performance and safety (individual level)

In both surveys, half of the participants either agree or strongly agree that changes in morale arising from the COVID-19 pandemic has negatively impacted on safety practices (46% in 2020 and 58% in 2021). A significant proportion of respondents in both surveys rate their competence and ability to do the job safety and to the required standard as deteriorated or greatly deteriorated, as compared with before the COVID 19 pandemic (25% in 2020 and 33% in 2021). However, a large number indicate no change in compliance with safety policies and procedures now, as compared to before the COVID-19 pandemic (63% in 2020 and 62% in 2021).

### Theme 5: impact on engagement and motivation (individual level)

In both surveys, most respondents either agreed or strongly agreed that changes in morale are negatively impacting on aviation worker engagement in work (69% in 2020 and 82% in 2021). Further, large numbers rated their motivation towards their job now, as compared to before the COVID-19 pandemic as having deteriorated or greatly deteriorated (47% in 2020 and 63% in 2021). Further, a large proportion of respondents in both surveys rated their level of engagement with their employer now, as compared to before the COVID-19 pandemic as deteriorated or greatly deteriorated (53% in 2020 and 70% in 2021).

### Theme 6: wellbeing, organisational priorities and wellbeing culture

**Fig. 28:**
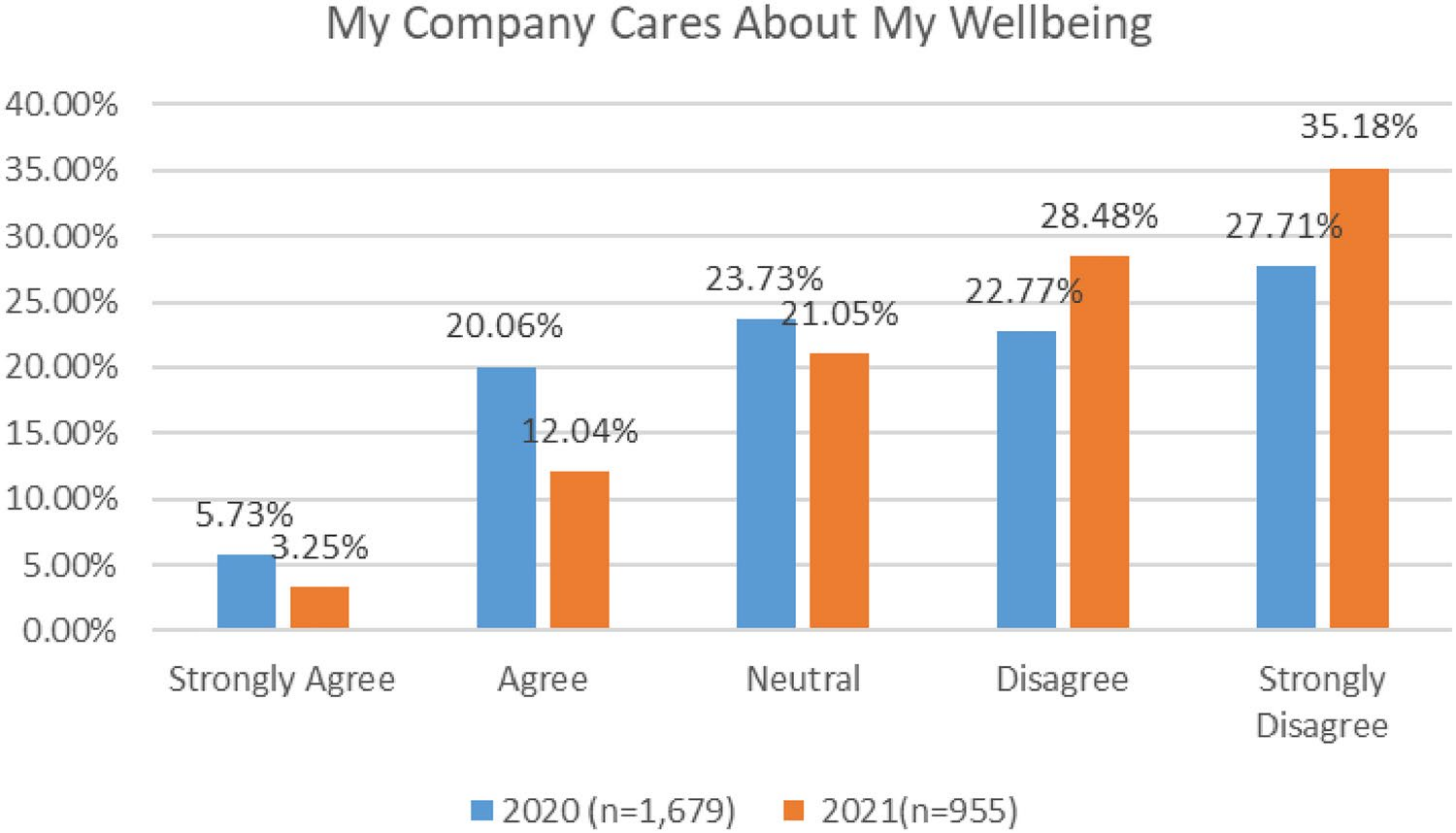
My company cares about my wellbeing—comparison 2020 and 2021 surveys

Both surveys elicited feedback about organisational priorities and wellbeing culture. As indicated in Fig. 28, a very low number of respondents (26%) in the 2020 survey either agreed or strongly agreed that their employer cares for their wellbeing. This number was particularly low (15%) in the 2021 survey.

**Fig. 29:**
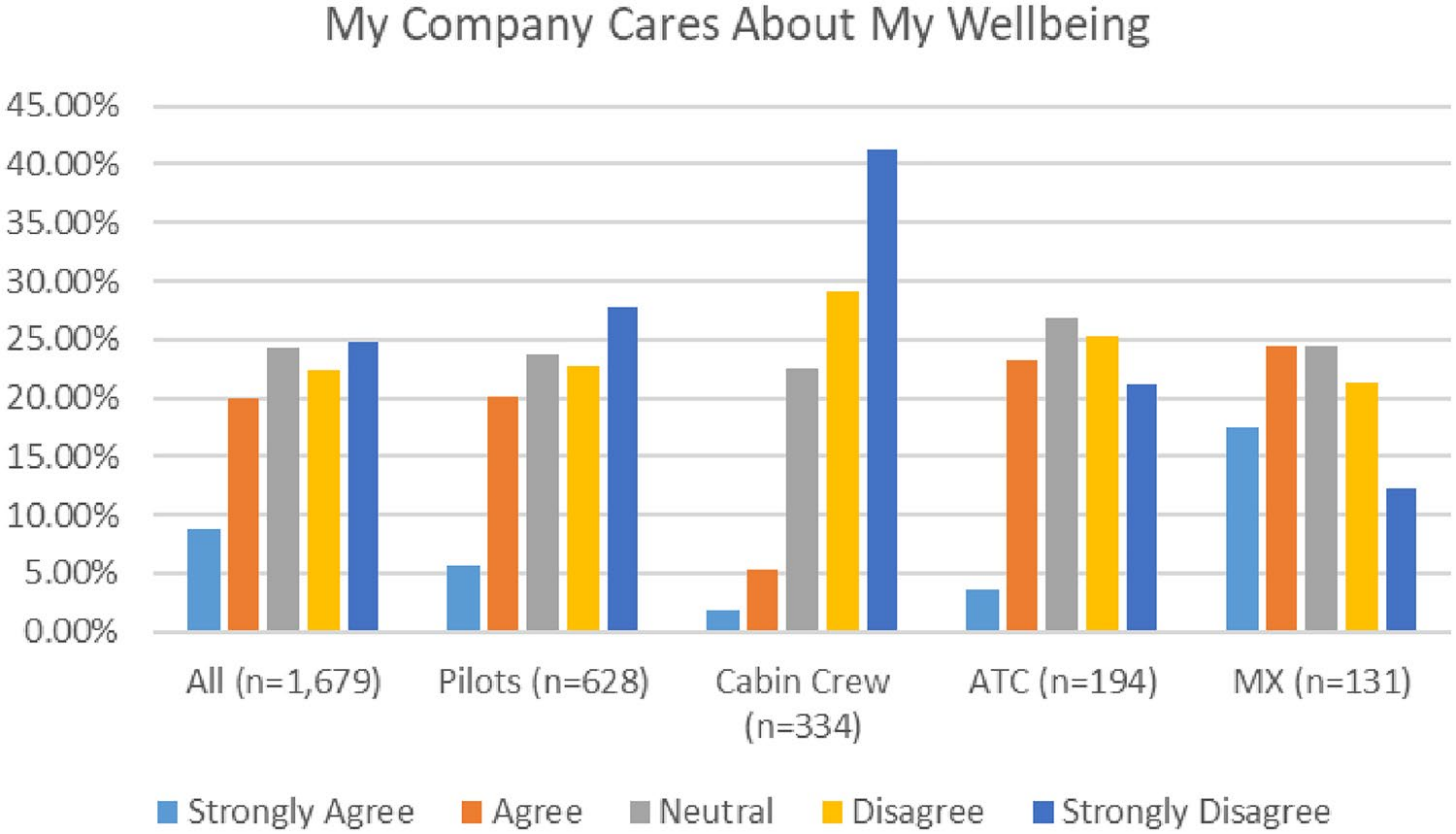
My company cares about my wellbeing, 2020

**Fig. 30:**
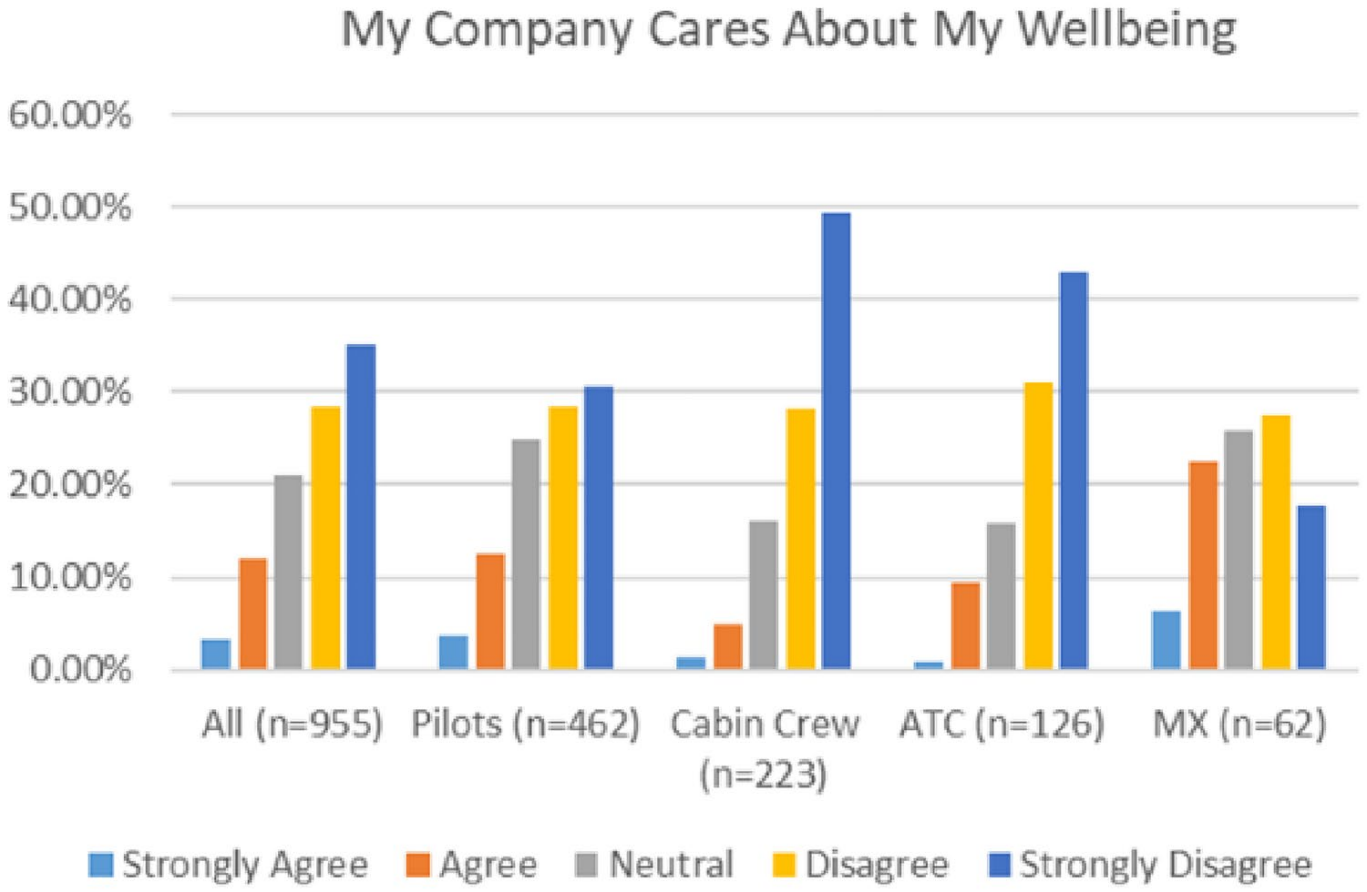
My company cares about my wellbeing, 2021

In both surveys, cabin crew and ATC reported the lowest level of agreement with the statement that “My Company Cares for My Wellbeing” (Figs. 29, 30).

**Fig. 31:**
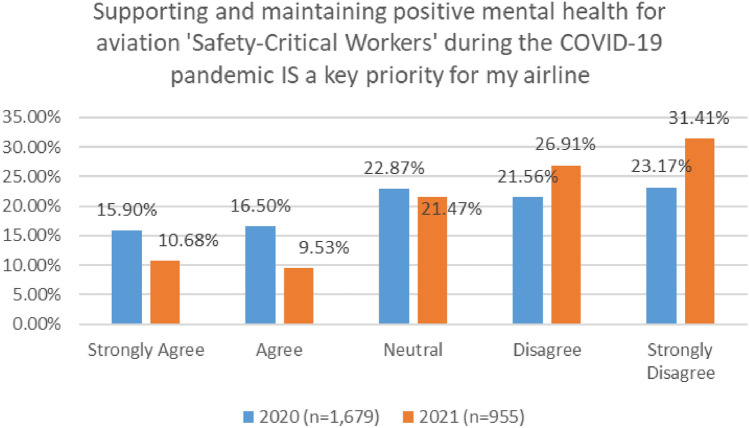
Comparison of supporting MH and company priorities, 2020 and 2021 surveys

There were significant differences between the feedback provided by participants in the 2020 and 2021 survey, in relation to aviation company priorities and supporting worker wellbeing during the pandemic (see Fig. 31). In 2020, a very small number of participants either agreed or strongly agree that supporting and maintaining positive mental health for aviation 'Safety–Critical Workers' during the COVID-19 pandemic is a key priority for their company/airline (32%). However, this number doubled to 64% in 2021.

**Fig. 32:**
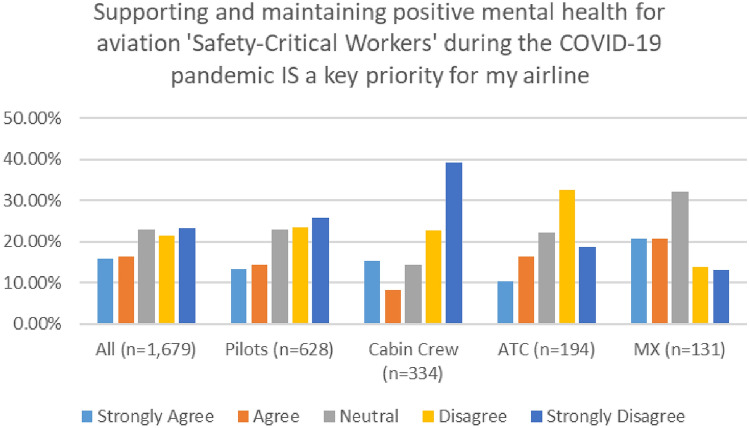
Supporting MH and company priorities—roles breakdown, 2020

**Fig. 33:**
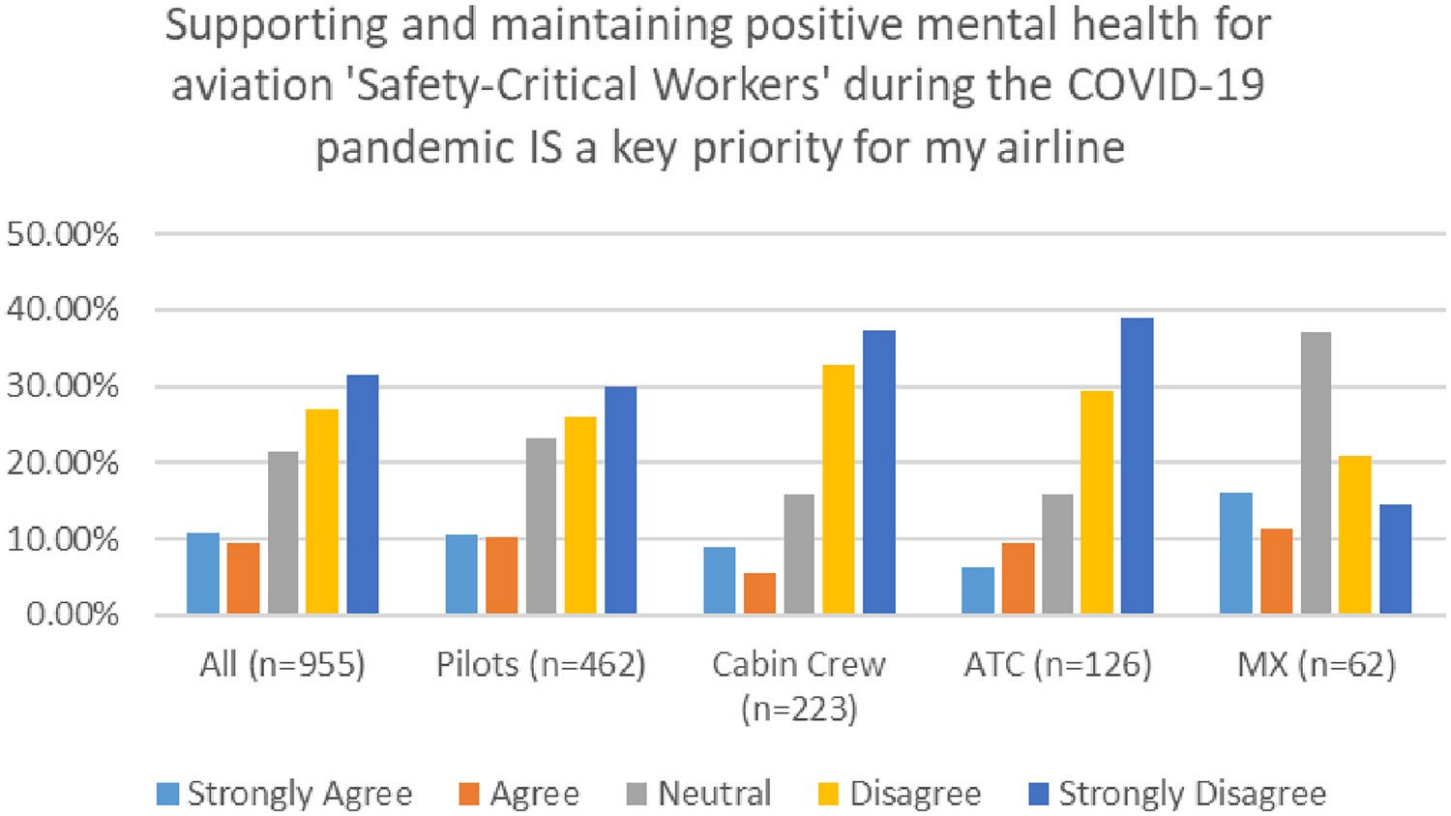
Supporting MH and company priorities—roles breakdown, 2021

A closer look at the findings of both surveys—indicates mostly similar trends across different workers groups, with cabin crew and ATC reporting the lowest level of agreement with this statement (Figs. 32, 33).

### Theme 6: wellbeing, organisational response to COVID and supports provided

**Fig. 34:**
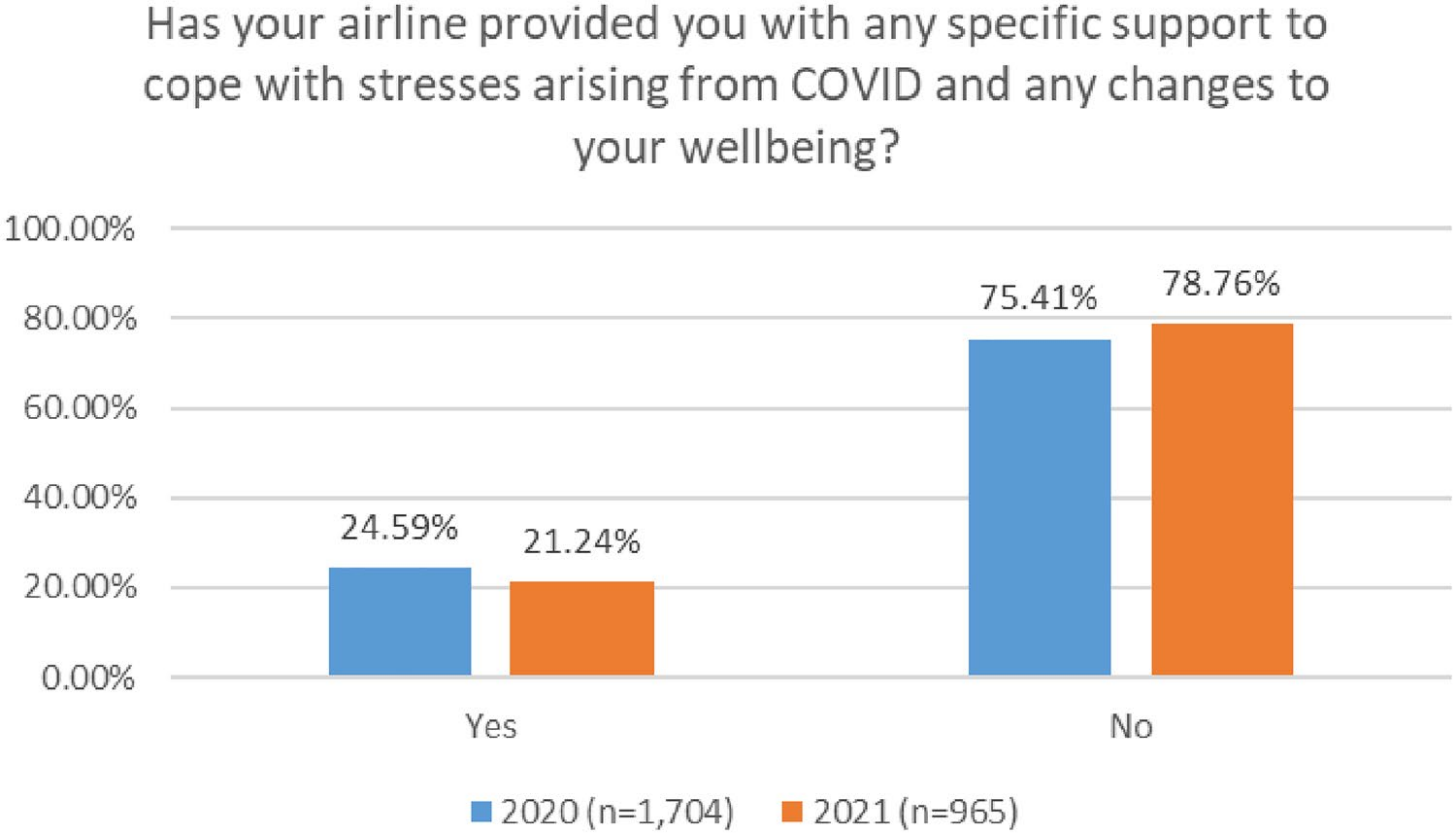
Company supports—comparison 2020 and 2021 surveys

As depicted in Fig. 34, a very low number of respondents received support from their company. In 2020, 25% of respondents reported that their company provided supports. This number dropped to 21% in the 2021 survey.

**Fig. 35:**
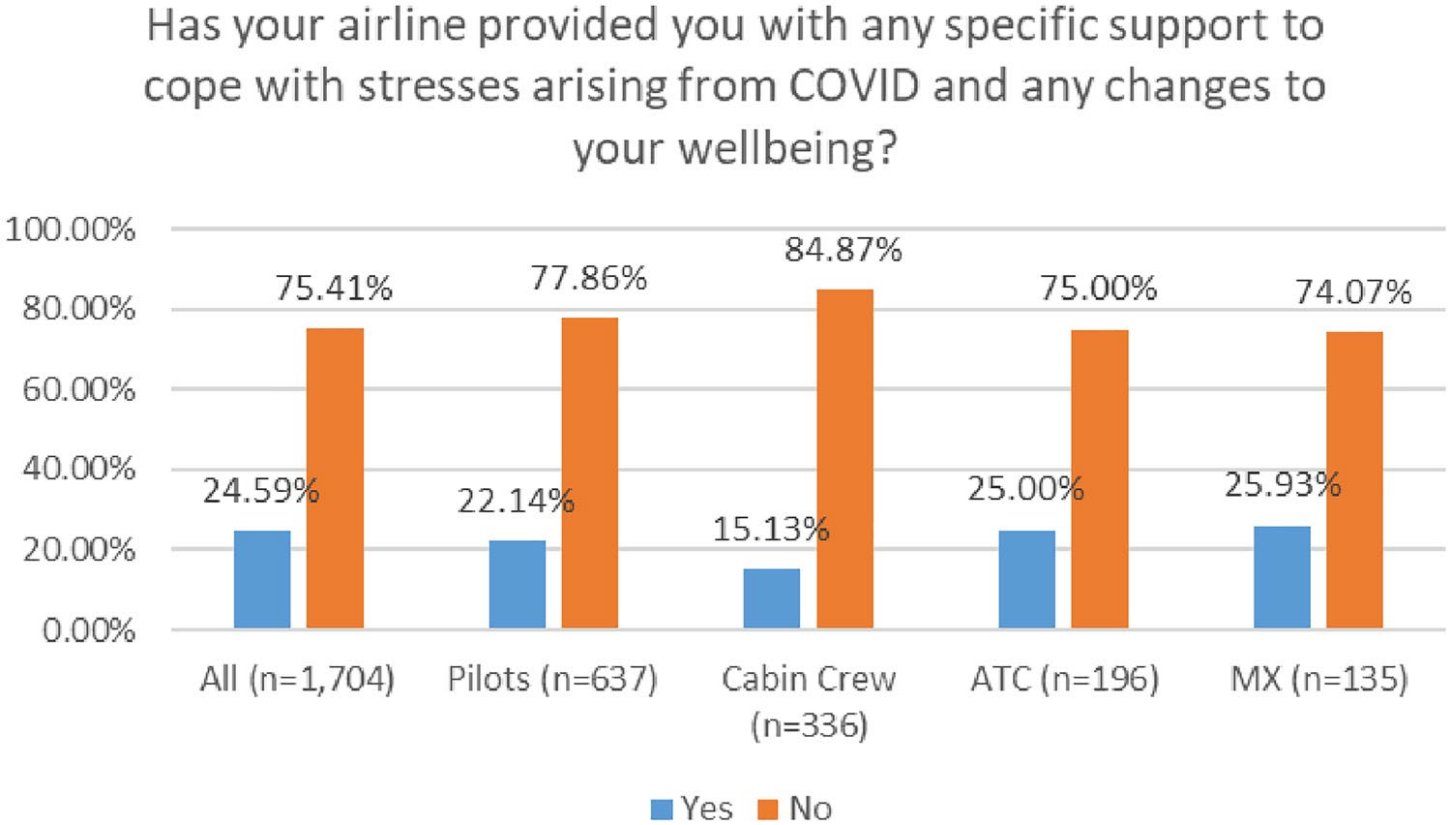
Company supports—roles breakdown, 2020

**Fig. 36:**
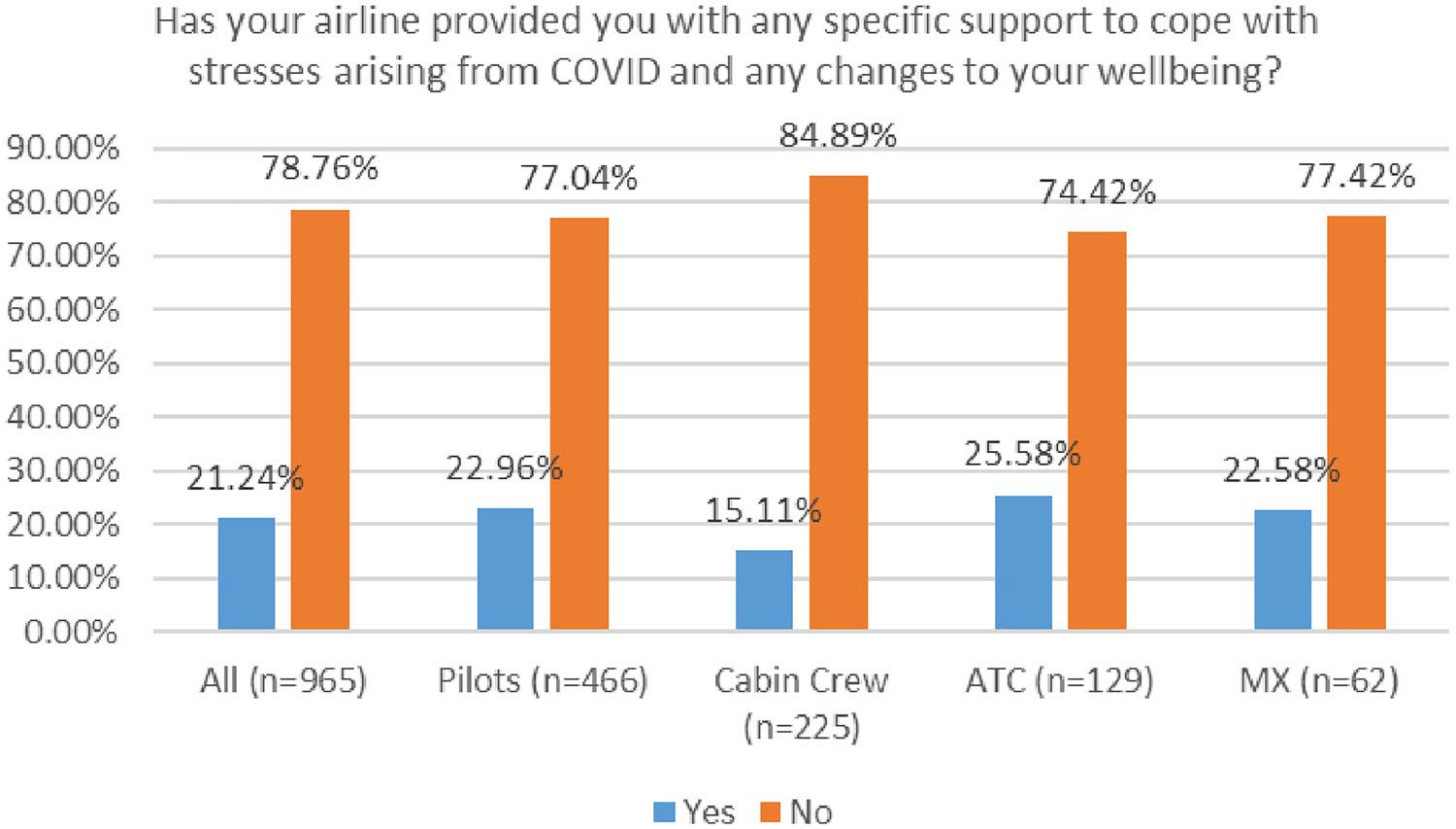
Company supports—roles breakdown, 2021

In both surveys, ATC personnel reported the highest level of employer support, with cabin crew reporting the lowest level of employer support (Figs. 35, 36).

In both surveys, respondents indicated that they would use company supports if provided (60% in 2020 and 57% in 2021). Of those who have been provided with supports, the numbers using these supports is very low (24% using company supports in 2020, and 25% in 2021). In both surveys, high numbers reported that they would approach peer support services, if needed (69% in 2020 and 67% in 2021). More respondents completing the 2021 survey indicated that they had used outside supports to help them cope with stress arising from the COVID 10 pandemic, and changes to their wellbeing (26.62% in 2021 versus 20% in 2020). However, in both surveys, the numbers are low. This raises questions in relation to the level of support those aviation workers who are experiencing suffering are receiving—outside the support provided by family and colleagues.

Respondents were invited to provide examples of the kinds of supports they received from their organisation. Of the 419 participants in the 2020 survey, who indicated that they were receiving supports, 80% (*n* = 335) provided examples of such supports. Of the 205 participants in the 2021 survey, who indicated that they were receiving supports, 57% (*n* = 116) provided examples of such supports.

An analysis of the free-text feedback across both surveys indicates that supports can be grouped into three overall types. These are

1. The provision of in-house company services/support.
2. The provision of access to outside services, paid for by the company.
3. Signposting to services outside the company (support groups, charities, Apps to use)

**Fig. 37:**
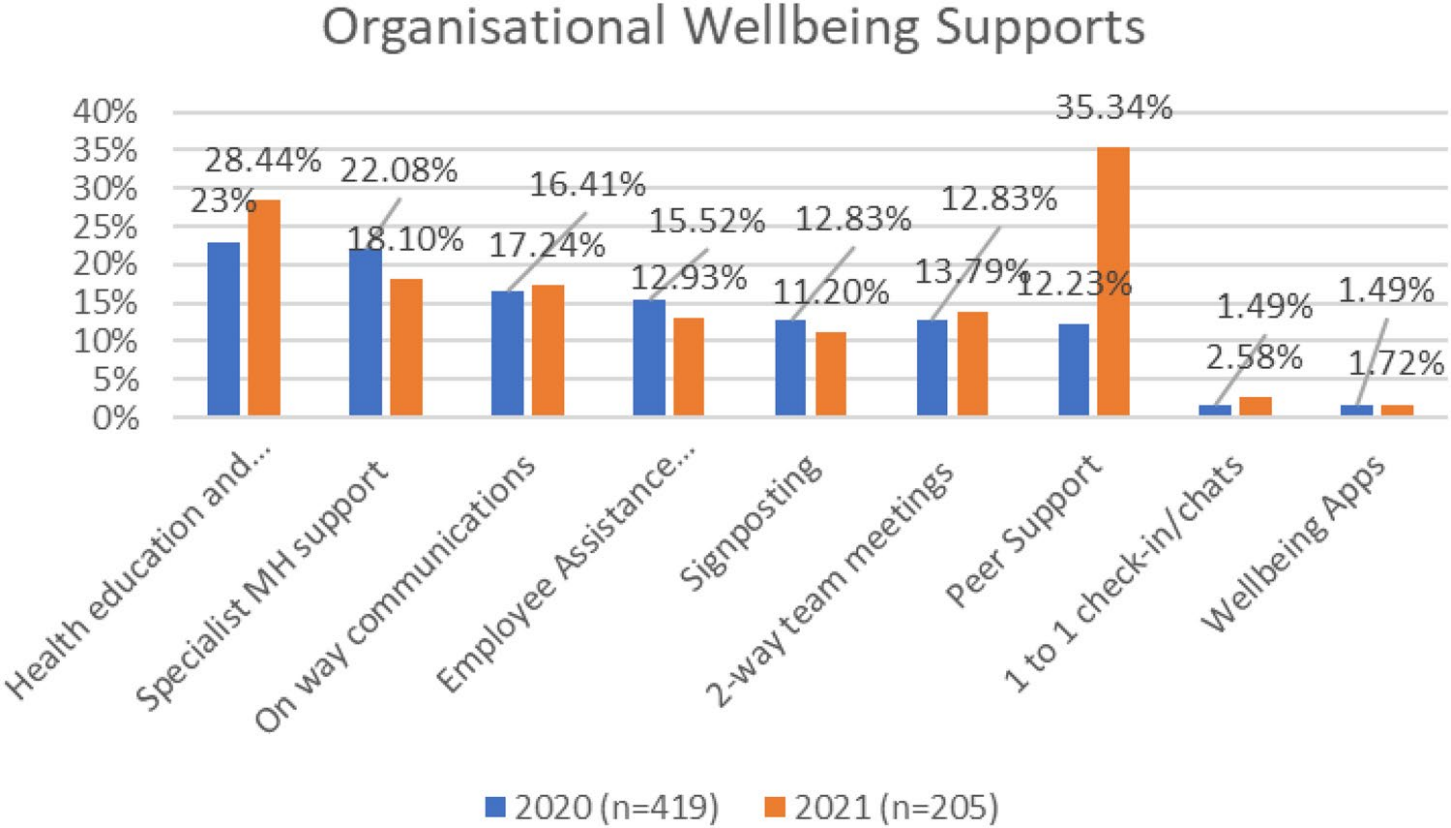
Comparison of organisational wellbeing supports, 2020 and 2021 surveys

As indicated in Fig. 37, both education and training and specialist mental health support appeared in the top three most frequently cited organisational supports in both surveys.

In 2020, the three most frequently cited supports were health education and training (23%), specialist mental health support (22%) and one-way emails/communications promoting mental health and wellbeing (16%). In the 2021 survey, peer support was the most frequently cited support provided by organisations (35%), followed by health education and training (28%) and specialist MH support (18%). Peer support was ranked seventh most frequently cited support (12%) in the 2020 survey.

Overall, organisations are implementing a multi-strategy approach—however, the focus appears to be on secondary and tertiary interventions, and not primary interventions (i.e., preventative). As indicated in Tables 3 and 4, the most frequently reported supports can be classified as secondary level interventions. Very few examples of primary interventions were provided. Respondent feedback indicates that the frequency of 1 to 1 chats/communications between different organisational functions/representatives and staff was very low (i.e., lack of personal connection/communication). Appendix 8 provides the full classification scheme and frequencies/counts for the different kinds of supports reported in both surveys.

**Table 3:** 2020 survey: example of organisational supports during COVID

Frequency ranking	2020	Type	Category
1	23% (*n* = 77)	Health education and training	Secondary
2	22.08% (*n* = 74)	Specialist MH support	Tertiary
3	16.41% (*n* = 55)	One-way emails/communications promoting wellbeing	Secondary
4	15.52% (*n* = 52)	Employee assistance program (internal/external)	Tertiary
5	12.83% (*n* = 43)	Signposting (internal/external)	Secondary
6	12.83% (*n* = 43)	Two-way team meetings to communicate updates and provide feedback/support	Tertiary
7	12.23% (*n* = 41)	Peer support	Tertiary
8	1.49% (*n* = 5)	1 to 1 check-in/chats	Secondary
9	1.49% (*n* = 5)	Wellbeing apps	Primary
10	1.19% (*n* = 4)	GP/physical health support	Tertiary

**Table 4:** 2021 survey: example of organisational supports during COVID

Frequency ranking	2021	Type	Category
1	35.34% (*n* = 41)	Peer support	Tertiary
2	28.44 (*n* = 33)	Health education and training	Secondary
3	18.10% (*n* = 21)	Specialist MH support	Tertiary
4	17.24% (*n *= 20)	One-way emails/communications promoting wellbeing	Secondary
5	13.79% (*n* = 16)	Two-way team meetings to communicate updates and provide feedback/support	Secondary
6	12.93% (*n* = 15)	Employee assistance program (internal/external)	Tertiary
7	11.2% (*n* = 13)	Signposting (internal/external)	Secondary
8	2.58% (*n* = 3)	1 to 1 check-in/chats	Secondary
9	1.72 (*n* = 2)	Wellbeing apps	Primary
10	1.72 (*n* = 2)	Exercise class	Primary

### Theme 7: requirements for wellbeing supports

The survey asked respondents about the requirements for supports to maintain their wellbeing. In both surveys, a very high number either agreed or strongly agreed that aviation workers who are currently working need support to maintain wellbeing during the COVID pandemic (94% in 2020 and 93% in 2021). Further, in both surveys, a very high number agreed or strongly agreed that supports are also required for those who are not working (92% in 2020 and 94% in 2021).

### Theme 8: COVID-19 pandemic and return to work

The survey also asked about the impact of COVID on fitness for work. In both surveys, a very high number agreed or strongly agreed that they will be fit to return to work (86% in 2020 and 81% in 2021 agreeing or strongly agreeing that they will be fit to return to work).

The survey also asked about fitness for work assessment. Survey feedback indicates a strong need for fitness for work assessment for aviation workers in safety critical roles (64% in 2020 and 61% in 2021 agreeing or strongly agreeing). Respondents indicated that this is also required for those who are not working (61% in 2020 and 67% in 2021 agreeing or strongly agreeing).

### Theme 9: safety oversight

Have there been changes from a safety oversight perspective? In both surveys, most respondents indicated that there has been no change to company safety practices since COVID 19 (53% in 2020 and 43% in 2021). Equally in both surveys, just over half of respondents reported no change to company safety oversight, since COVID 19 pandemic (57% in 2020 and 53% in 2021). However, a significant number of respondents rated the level of safety oversight from within their company now, as compared to before the COVID-19 pandemic as deteriorated or greatly deteriorated (28% in 2020 and 38% in 2021). Lastly, most respondents indicated no change to safety oversight from their national regulator (59% in 2020 and 60% in 2021).

### Regression analysis/linear probability models

As indicated in Appendix 11, the null hypothesis of no relationship was rejected for twenty-five of the thirty-six linear probability models. In most cases, the p values were very low (very significant). Appendix 12 provides a breakdown of r squared, coefficients, p values for coefficients and confidence intervals for the different models.

There was a relationship between those who have spoken to somebody about their MH and/or would speak to somebody and aviation worker wellbeing levels. That is, those who have spoken to or would speak to somebody about their wellbeing had higher depression scores (model 1), higher anxiety scores (model 3) and lower happiness and life satisfaction scores (model 2).

There was a relationship between those who are obtaining company supports and aviation worker wellbeing levels (see models 4, 5 and 6). That is, those who are obtaining company supports had lower depression scores (model 4), lower anxiety scores (model 6) and higher happiness and life satisfaction scores (model 5).

There was a relationship between those who strongly agreed that their airline cares about their wellbeing and aviation worker levels of wellbeing. That is, those who strongly agree that company cares about their wellbeing had lower depression scores (model 7), lower anxiety scores (model 9) and higher happiness and life satisfaction scores (model 8).

There was a relationship between those who ‘strongly agreed’ that they would look for help for MH issue and depression scores (model 10). That is, those who strongly agreed to look for help had lower depression scores. However, the relationship between ‘strongly agreed’ to look for help for MH issues and anxiety scores was not found to be statistically significant (model 12) and there is some uncertainty in the analysis for happiness scores—given the confidence intervals (models 11).

There was a relationship between those who ‘strongly agreed’ to be willing to disclose MH issues to their employer and aviation worker levels of wellbeing. That is, those who ‘strongly agreed’ to be willing to disclose MH issues to their employer had lower depression scores (model 13), lower anxiety scores (model 15) and higher life satisfaction/happiness scores (model 14).

There was no evidence of a relationship between coping strategies and depression scores (model 16), anxiety scores (model 18) and life satisfaction scores (model 17).

There was no evidence of a relationship between those using company supports and depression severity levels (model 19), anxiety severity (model 21) and happiness levels (model 20).

There was a relationship between those using outside supports and aviation worker wellbeing levels. Those using outside supports had higher depression levels (model 22), higher anxiety levels (model 24) and lower life satisfaction and happiness levels (model 23).

There was a relationship between those strongly agreeing that changes in morale had impacted on safety practices and aviation worker wellbeing levels. Those strongly agreeing had higher depression levels (model 25), higher anxiety levels (model 27) and lower life satisfaction and happiness levels (model 26).

There was no evidence of a relationship between those reporting that their competence and ability to do the job safety had deteriorated since COVID and depression severity levels (model 28) and happiness levels (model 30). However, a statistically significant relationship was identified between those who reported that their competence and ability to do the job safety had deteriorated since COVID and anxiety levels (model 29—those reporting deteriorated had higher anxiety levels).

There was no evidence of a relationship between those who rated compliance with safety policies and procedures now, as compared to before the COVID-19 pandemic as deteriorated, with depression severity levels (model 31) and anxiety severity levels (model 33). However, a statistically significant relationship was reported in relation to those who reported that compliance with safety policies and procedures now, as compared to before the COVID-19 pandemic as deteriorated, and happiness levels (model 32—those reported deteriorated had lower happiness and life satisfaction scores).

Lastly, there was a relationship between those rating that their job motivation had deteriorated since before the pandemic and aviation worker wellbeing levels. Those who rated that their job motivation as having deteriorated since COVID had higher depression levels (model 34), higher anxiety levels (model 36) and lower life satisfaction and happiness levels (model 35).

## Discussion

### Overall narrative: culture of supporting and reporting

Arguably, it makes sense to look at the narrative emerging from the overall findings. This includes patterns arising from the descriptive statistics for both surveys (i.e., 2020 and 2021), along with the findings of the regression analysis for the 2021 survey. Taken collectively, the research evidence paints at consistent story around the poor culture of reporting (i.e., MH disclosure) and the poor culture of supporting aviation workers. There appears to be significant barriers to reporting MH challenges, with a weak disclosure culture. As indicated in both surveys, (1) MH discussion amongst aviation workers is low (stigma), (2) aviation workers seem more likely to disclose MH issues to those they have close personal bonds with (i.e., spouse or family), and (3) many staff are not reporting MH issues to their employers. Critically, in both surveys, a very low number of aviation workers reported that they would willingly disclose a mental health issue to their employer. Trust is necessary for reporting, but it cannot be assumed. Arguably, there is a gap between the organizational vision of both supporting and enabling a ‘just culture’ and ‘psychological safety’ for workers, and the ‘lived experience’ of job. Further, both surveys indicate that aviation workers do not perceive wellbeing as a priority for their employers. In addition, both surveys indicate that the response of aviation organisations to supporting worker wellbeing during the pandemic has been weak. Evidently, workers need to be motivated to report wellbeing issues. This depends on employers taking wellbeing issues seriously, on the provision of safeguards to protect reporting, and on employers acting on challenges, when identified. Accordingly, there is a relationship between organisations providing wellbeing supports and protections, the perception by staff that wellbeing is a priority, and staff willingness to report.

In particular, the regression analysis in relation to the 2021 survey results paints a compelling narrative. The analysis indicates a statistically significant relationship between respondents reporting lower levels of wellbeing and deteriorating job motivation. Equally it indicates that respondents who strongly agreed that changes in morale have negatively impacted on safety practices, have lower levels of wellbeing. A statistically significant relationship was identified between those who feel their airline cares about their wellbeing and positive wellbeing (i.e., those who strongly agree have lower depression and anxiety scores and higher happiness and life satisfaction scores). It also shows a significant relationship between willingness to disclose MH issues to their employer and positive levels of wellbeing (i.e., strongly agree to be willing to disclose MH issues to their employer and low depression and anxiety scores and high life satisfaction/happiness scores). There was evidence of a relationship between aviation workers with significant level of suffering and use of external supports. However, there was no evidence of a statistically significant relationship between those have poorer levels of wellbeing and use of company supports. Collectively, this raises questions around (1) MH stigma, (2) lack of disclosure within the company (fear, stigma), (3) the reliability of company wellbeing reporting, and (4) the efficacy and suitability of company supports to support employee wellbeing and mental health and (5) the risk associated with the level of support that aviation workers with low levels of wellbeing are obtaining.

### Aviation worker wellbeing

Overall, this research highlights the need to address levels of wellbeing for all aviation workers including pilots. Wellbeing levels for aviation workers appear to have worsened since the onset of the COVID 19 pandemic. Specifically, the numbers of pilot’s screen positive on the PHQ9 for depression has risen from 17.04% (pre-COVID) to 24.2% (2020 Survey). However, the findings of the 2021 survey indicate a small improvement in wellbeing levels for pilots as compared with 2020. Here, the numbers of pilots screening at or above the threshold for moderate depression on the PHQ9 has reduced from 24.2 to 22.5%. It should be noted this same pattern is reflected for all aviation workers, although the numbers of ‘all aviation workers’ screening at or above the threshold for depression on the PHQ9 is higher overall (dropping from 29.6 to 21.7%). Critically, wellbeing trends for pilots and all aviation workers are different to what has been reported in studies of the general population both pre and since COVID and during COVID. As noted previously, the Shelvin study (2021) points to an overall improvement trend in relation to wellbeing levels in the general population, both pre and since COVID and during the COVID timeframe. This improvement trend is not found in our study.

Research undertaken with pilots prior to pandemic indicated that pilots have higher levels of suicidal ideation than the general population (Wu et al. 2016; Cahill et al. 2021). The two COVID surveys suggest that all aviation workers have higher levels of suicidal ideation that the general population, and that this has improved slightly since the start of the pandemic (numbers screening positive for suicidal ideation dropping from 11.6% in 2020 to 11% in 2020). Further, in relation to both the 2020 and 2021 surveys, levels of suicidal ideation appear lower for pilots, as compared with all aviation workers.

### Organisational wellbeing supports

Survey feedback highlights the need for aviation organisations to address wellbeing, and in some cases, to adjust leadership and organisational priorities. Overall, there has been a weak response from organisations in terms of helping employees cope with the stress arising from the COVID 19 pandemic, and changes to their wellbeing. Of those who have been provided with supports, the numbers using these supports is very low. This does not tally with the large numbers who appear to be seeking supports. Potentially, this is an indicator that the supports provided are not meeting the need. Further, in both surveys, a small number of respondents indicated that they had used outside supports to help them cope with stress arising from the COVID 10 pandemic, and changes to their wellbeing (26.62% in 2021 versus 20% in 2020). This raises questions in relation to the level of support those aviation workers who are experiencing suffering are receiving—outside the support provided by family and colleagues.

As evidenced in this research, organisations are implementing a multi-strategy approach. However, the focus appears to be on secondary and tertiary interventions. Currently, there is insufficient emphasis on primary interventions (i.e., preventative). Aviation organisations need to rethink their objectives and approach in terms of the provision of primary interventions. Secondary and tertiary interventions are not enough. Anticipating risks is a crucial first step in relation to building a preventative OSH culture. Proactive and predictive risk assessment techniques should be used. Critically, wellbeing and psychosocial risk need to be managed in aviation organisations SMS.

Suffering is not equal across different aviation worker profiles. As reported earlier, survey findings indicate that both cabin crew and maintenance/engineering personnel are doing less well than Pilots and ATC and that this trend has continued during the COVID timeframe. Supports need to be considered for all aviation workers, and not just pilots. Critically, the implementation of company supports needs to address issues around MH stigma and the need for privacy.

Aviation organisations need to provide appropriate wellbeing supports for those currently in work and off work. Those people who have lost their jobs and/or are experiencing MH difficulties require immediate support. Fitness to work assessment is required for those currently working and those returning to work.

The findings of the preliminary regression analysis support the case for addressing the provision of wellbeing supports for aviation workers, along with addressing the wellbeing culture within aviation organisations. For example, the analysis indicates a statistical relationship between those obtaining company support and aviation worker wellbeing levels (i.e., those obtaining support have lower depression and anxiety scores and higher happiness and life satisfaction scores). Obviously, other factors might influence these scores. Further, this analysis does not address the nature of the support provided (i.e., primary, secondary, or tertiary) and/or the specific type (i.e., if tertiary—peer support or access to psychologist). Nonetheless, it can be treated as emerging findings that requires further exploration.

### Wellbeing, performance and safety

Both surveys provide insights to the relationship between wellbeing, performance, and safety, and underscore the sense in which wellbeing can be considered a ‘protective factor’ for safe performance. Also, both surveys demonstrate a reduced focus on, or prioritisation of safety, human and organisational factors—specifically, supports for wellbeing and fitness for work. As reported by survey respondents, changes in morale have had an impact on performance/professionalism and safety. Yet, there is an insufficient focus on wellbeing, and in particular—promoting discussion of wellbeing in the context of performance and safety. Arguably, existing safety concepts and approaches will need to be extended to reflect the importance of wellbeing as a factor in safe performance. Aviation organisations need to implement an integrated health, wellbeing, and safety culture.

Aviation organisations require education as to what a good health and wellbeing culture looks like and how to link this to safety culture. A good wellbeing culture includes a social environment where staff feel comfortable talking about wellbeing challenges and reporting sick, where supports are provided to staff, and where staff wellbeing/MH levels are measured. Staff need to be encouraged to put their hands up if they are experiencing difficulties. Critically, staff will not do this if they believe the outcome will be punitive (i.e., loss of license, impact on career progression). Stigmatisation and lack of reporting is a huge barrier to fostering a wellbeing culture, along with implementing an ‘integrated health and safety culture’.

### Coping and resilience

As indicated in this survey, many respondents are using coping strategies. In principle, the use of coping strategies has a positive impact on health, with benefits for performance and safety. Although there was no evidence of a significant relationship between coping strategies and depression scores (model 16), anxiety scores (model 18) and life satisfaction scores (model 17), a lack of significance does not mean that the coping strategies are not beneficial. Coping strategies are practiced by those of varying levels of wellbeing—to enhance positive welling, to mitigate against stress and potential ill health and to deal with ill health (including mental health). Self-care approaches need to be promoted both from a wellbeing and a safety promotion perspective. Staff need to be trained in wellbeing MH awareness, stress management/coping behaviour, wellbeing/MH risk identification, and in how to support others who are suffering. As such, the provision of wellbeing training will bolster wellbeing culture and impact on performance and operational safety levels.

### Addressing positive wellbeing

The aviation industry manages ‘worker wellbeing’ from the perspective of addressing fitness for work issues, and the management of operational safety/risk. The focus is on detecting illness and/or the presence of factors that might negatively impact on safe performance (for example, fatigue or intoxicants). There is little focus on promoting positive wellbeing and preventing illness. As indicated in survey results, there were sparse examples of interventions focussed on maintaining positive wellbeing and/or promoting positive wellbeing. Recent research identifies the need for aviation organisations to develop a human factors and systems-based workplace health and safety strategy, promoting positive wellbeing (i.e., flourishing), while also addressing illness prevention and the management/mitigation of suffering (Cahill et al. 2018; 2019). As indicated in this research, existing health and safety approaches adopted by aviation organisations fall short of this.

### Gathering wellbeing information, trust and just culture

Arguably, aviation organisations will need to think differently about obtaining reporting information. As indicated in this research, trust is not a given. Across both surveys, only one in four aviation workers would willingly disclose a MH issue to their employer. This would require careful attention to issues of data protection. From an implementation perspective, trust is obtained via staff protections and safeguards. In this way, de-identifying information (i.e., protecting employees/staff) is a route to both obtaining wellbeing data (i.e., evidence), to assessing wellbeing risk, and to building a culture of wellbeing. So how might deidentified data be obtained? This can involve informal reporting, making use of de-identified information from different stakeholders (for example, LOSA observers, airline psychologists and peer assistance networks). Further, emerging technologies might be used to collect de-identified data from staff. This might span information about staff wellbeing both while on and off duty (Cahill et al. 2020a, b).

### Moving beyond current regulatory approach/compliance

Given the existing low reporting levels, the fact that very few organisations are providing supports, and that existing supports such as peer support, are not being used, the regulator needs to address the suitability of the existing approach to managing wellbeing and mental health. Addressing wellbeing for aviation workers involves mapping the problem from a human factors/systems perspective, and in particular addressing dependencies between reporting culture, organisational culture, job protection, licence protection and aeromedical assessment.

Overall, a shift in focus from the employee to the employer is required. There needs to be a greater emphasis on the role of the employer—linking to the concept of responsible work. Evidently, aviation workers have responsibilities too. They must manage their own health and wellbeing.

Critically, this research supports an argument that the regulator needs to move beyond the three strategies of (1) drugs/alcohol testing, (2) mental health assessment and (3) peer support for pilots. In terms of addressing issues around the detection of wellbeing/MH problems, promoting positive wellbeing, and supporting those who are suffering, these strategies fall short of what is required. Further, this research supports a case that the regulator needs to provide a roadmap and recommended approach in relation to supports provided for other aviation workers (i.e., beyond pilots).

Worker’s health (including MH) can fluctuate. Aviation organisations need to acknowledge that it is not enough to recruit the right person for the job (i.e., using recruitment/psychometric tests based on idea of right person and select in and select out criteria). Operational and organisational processes need to foster and protect wellbeing. All stakeholders (i.e., regulator, aviation organisations and employees) must address positive wellbeing while also managing suffering and fitness for work issues. Further, promoting positive wellbeing for all aviation workers should not be overlooked.

In addition, there is a need to extend the existing strategy for wellbeing management, to ensure an integrated approach to health/wellbeing and safety issues, and to manage wellbeing as a risk within an organisation’s SMS. Wellbeing risk needs to be managed in airline safety management systems (SMS), as part of a preventative approach (i.e., primary interventions). This involves augmenting existing data collection approaches, to consider wellbeing risk, and specifically, the gathering of data pertaining to the relationship between wellbeing, performance, and safety. Reporting and disclosure methodologies and processes must address issues around trust and privacy—in particular, the concerns that safety critical staff have about maintaining their licences and ensuring continuity in livelihood. Ideally, an acceptable means of compliance (AMC) for the management of wellbeing at an organisational level is required. Such an AMC should address needs and barriers, in relation to reporting and supporting processes.

### Change, ethics of care, leadership and responsible work

Changing aviation wellbeing culture requires social change at an organisational level (Schein 1992). It will be difficult to motivate aviation organizations to change, in the absence of regulation compelling them to do so. Regulation provides a key motivation for aviation organisations to change their processes and/or adjust values. Ideally, as part of a future roadmap, the regulator might extend the existing approach, in terms of defining compliance requirements to ensure that work practices are designed to promote wellbeing and avoid WRS. This will take time, and yet the industry requires action now. Given the COVID situation, the attention of aviation organizations is most likely to be elsewhere (i.e., maintaining the business). Nonetheless, aviation organizations still need to focus on their people, and to ensure that the workforce is healthy. Aviation organisations need to be encouraged to move beyond regulation and to consider their ‘duty of care’. A strong starting point would be the adoption of an ‘ethics of care’ (Gilligan 1982; Tronto 2005) approach. This might be incorporated in aviation organisation strategic vision documents and reinforced by management ethos, decisions, and behaviours. Articulating this approach is particularly important in relation to the management and use of employee wellbeing data in safety management systems. Processes for safeguarding/protecting employee wellbeing data, and the use of wellbeing information in safety/risk management processes need to be defined. Some airlines are embracing change and demonstrating leadership by locating wellness/wellbeing within the sphere of existing safety management systems. Although, this research is at an early stage, it is promising.

## Research limitations and areas for further research

Both surveys involved convenience sampling. There may have been bias as regards the participation of aviation workers (i.e., those interested in wellbeing, those suffering, those who have issues with their organisation’s management of wellbeing). The survey method does not allow digging deep into many of the issues or mapping the complexity across issues. This requires further ethnographic research—in particular, detailed interviews and workshops with different stakeholders. The COVID surveys ran during two time periods. The COVID situation is dynamic. The situation of workers may have changed (disimproved or not). Further, some aviation organisations may have changed their approach to supporting wellness.

The preliminary regression analysis (simple regression models) supports the case for addressing the wellbeing culture within aviation organisations. Importantly, the individual models address twelve single independent/predictor variables and three outcome variables. As expected, the R squared for each of the models is low. More complex models (multiple regression models) are required to explain all factors that influence the dependent variables (i.e., depression severity, anxiety severity and happiness/life satisfaction level). However, the models and associated *p* values provide evidence of statistical relationships between predictor variables and outcomes. Nonetheless, these emerging finding requires further analysis using more multiple regression models (combination of predictor variables). Further, deeper analysis is required to examine relationships across health outcomes. A future analysis might explore models suitable to the discrete nature of the outcome/*Y* variable (i.e., happiness, depression, and anxiety scores. This would involve implementing ordered and multinomial logistic models.

It is necessary to further investigate what an integrated approach to health, wellbeing and safety will look like, and how it might be implemented in terms of specific organisational processes and procedures. It would be useful to obtain the perspective of safety managers, occupational health and safety personnel and human resources in relation to supporting wellbeing for aviation workers. This research might focus on capturing information on the existing approach (work as done versus work as imagined), and a future approach. Further, will be important to examine organisational health and wellbeing culture, and associated indicators in more detail. Stakeholders will have differing perspectives. These perspectives influence how they think about supporting wellbeing and implementing solutions that deliver value for organisations and their staff, along with linking into the regulatory picture (which itself is evolving).

Further research is required to advance how reporting about wellbeing might be integrated with safety reporting processes and assessed in aviation safety management systems. This requires advancing a real evidence picture and promoting reporting around wellbeing challenges and the relationship between wellbeing, performance, and safety, at different levels. Crucially, information about wellbeing risk needs to be made use of in the operation (i.e., flight planning, crew rostering), and in wider organisational activities (i.e., training, providing supports to those who are suffering). Currently, this concept is in its infancy.

An integrated approach to health, wellness and safety necessitates making use of data about wellbeing and its relationship to safety in an SMS. With increasing use of operational intelligence and reporting systems and machine learning/AI—there is an opportunity to use technology to further enhance safety approach. Research will need to investigate the use of emerging technologies to support collecting data from operational staff, to support this.

## Conclusions

Feedback from both surveys indicates that aviation workers are experiencing considerable challenges in relation to their wellbeing. This includes challenges arising from sources of WRS and their impact on wellbeing, and the impacts of COVID 19. These challenges are not being adequately addressed at an organisational level, which creates risk at an individual level (impact on individual wellbeing) and a flight safety level (impact of wellbeing problems on performance and flight safety). Preliminary statistical analysis supports the case for airlines to address their wellbeing culture the disclosure/reporting culture and the provision of wellbeing supports.

Currently, wellness approaches at an aviation organisational level are not addressing both human and operational safety needs. They are defined negatively (in relation to illness as opposed to positive wellbeing). Further, workers bear the responsibility for fitness for work, and maintaining wellbeing. Aviation organisations have a role in relation to supporting worker wellbeing and managing sources of WRS and psychosocial hazards/risk. Aviation organisations need to address issues pertaining to their wellbeing culture—promoting healthy behaviour, supporting disclosure around mental health issues/challenges, promoting awareness of MH.

Both organisations and workers need to manage specific sources of stress (including work-related stress) and anxiety, and the specific impact of COVID 19 on aviation workers. Aviation workers across different roles are practising self-care—this should be encouraged at all levels—linking to promoting a wellbeing culture and safe behaviour. Aviation organisations need to enable workers to practice healthy behaviours—which are enabled by providing better support to workers in terms of the management of the home/work interface.

This research highlights the need for a system level/human factors response to the management of wellbeing and mental health for aviation workers. The roles and responsibilities of different stakeholders (i.e., workers, organisations, and the regulator) in relation to managing wellbeing require rethinking and clarification. Most importantly, wellbeing needs to be treated as a responsibility that is shared between aviation workers and their employers. This will require regulatory change, cultural change (i.e., the culture of reporting and supporting), and process change (i.e., implementing new processes and methodologies to embed wellbeing in the design of work, along with implementing new processes and methodologies to support wellbeing management for employees).

Addressing wellbeing for aviation workers involves mapping the problem from a human factors/systems perspective. All stakeholders need to identify a path to integrating different wellbeing functions within aviation organisations (i.e., peer support, EAP, safety/risk management, health promotion)—linking to aeromedical assessment and regulation. This might be ‘guided’ by the regulator. This research has identified three core stakeholders who have leverage over managing wellbeing—the (1) regulator, (2) aviation organisations and (3) workers.

Organisations need to advance an ethical and evidence-based strategy to managing wellbeing. An improved wellness management approach requirements focusing on positive wellbeing, in addition to preventing suffering and managing/mitigating suffering. Overall, the objective is to promote the protective factors, reduce the risk factors, and prevent mental ill health. Primary interventions need to address both prevention and promoting positive wellbeing. Secondary and tertiary interventions should address suffering at different levels, involving the use of expert clinical supports, where required. In line with Safety II approaches, this involves collecting data about the relationship between wellbeing, performance, and safety at different levels. This depends on trust and the specification of appropriate protections so that they can safely report wellbeing issues. Critically, the ethical underpinnings of such interventions require careful consideration. This includes a consideration of ethical issues pertaining to concepts of responsibility, information culture, processes for safeguarding/protecting employee wellbeing data and the use of wellbeing information in risk management processes (i.e., acquiring information about hazards/risk, acting on information/making informed decisions and allied communicating processes). Adopting an ethical approach to occupational health and safety requires real leadership.

In addressing wellbeing, aviation organisations and aviation workers may need to move beyond a compliance-based approach. Further, the regulator might provide oversight/support to aviation organisations in relation to going beyond compliance requirements.

Aviation organisations need to consider their accountability across the triple bottom line (i.e., economic, society and ecological). Promoting a wellbeing culture will require leadership support and an alignment of health and business objectives—linking to concepts of corporate social responsibility and responsible work. The management of wellbeing risk within an SMS will go some way towards addressing this.

## Appendices

### Appendix 1: survey topics and measures

**Table:**

#	Topic	Research question	Measures
1	COVID 19 pandemic and impact on wellbeing	What is the impact on aviation worker wellbeing and mental health?	Rating of wellbeing—physical Rating of wellbeing—psychological and emotional Rating of impact of COVID on mental health Impact on mental health—depression (PHQ 9) Impact on mental health—anxiety (GAD 7)
2	COVID 19 pandemic and impact on job and employment	What is the impact in terms of job/employment?	Financial wellbeing Job loss Employment terms (reduced salary/hours) Perception of job security Confidence in future for company
3	COVID 19 pandemic and impact on performance and safety (individual level) and safety oversight (organisational level)	What is the impact on (1) performance and (2) flight safety?	Perception of fitness to do the job Impact on engagement Impact on safety behaviours/procedures Rating of change in relation to company safety practices since COVID Rating of change in relation to regulator safety oversight since COVID
4	COVID 19 pandemic and self-care/seeking help	Are aviation workers coping? Demonstrating healthy behaviours? Seeking help, if needed?	Use of coping strategies (self-management)? Willingness to seek support/help if needed? Willingness to use organisational supports if provided? Existing use of organisational supports—if provided? Use of supports/services outside organisation
5	Attitudes to MH and disclosure	Attitudes to MH and talking about MH? Willingness to disclose MH issues to others and employer	Talking about MH Attitudes to disclosing MH issues Willingness to disclose to others Willingness to disclose to employer
6	COVID 19 pandemic, requirements for support and organisational wellbeing culture	Is wellbeing a priority for organisations? Are existing organisational supports fit for purpose? Willingness to disclose MH issues to employer	Perception of whether wellbeing is a priority for worker’s organisation Provision of peer support Are organisations providing wellbeing supports—since COVID? Are existing supports being used by aviation workers? Willingness to disclose MH issue to employer
7	COVID 19 pandemic, remote work, impact, and change	What has the experience of remote work been like for aviation workers? How has it impacted their work and home/work interface?	Perception of impact on productivity Perception of impact on workload Perception of impact on work efficiency Perception of impact on teamwork Perception of impact on home/work interface
8	COVID 19 pandemic and need for supports	Do aviation workers in safety critical roles need support in relation to maintaining their wellbeing during pandemic (1) currently working, (2) currently not working	Perception of need for supports for workers currently in work Perception of need for supports for workers currently off work
9	COVID 19 pandemic, return to work and requirements for aeromedical assessment	Do aviation workers need require some form of fitness to work assessment on returning to work	Requirements for all Requirements for safety critical workers

### Appendix 2: respondent profiles

#### COVID survey 1: 2020

The survey was completed by 2,050 aviation workers. 2,050 respondents participated in the survey, with 1,523 completing it fully (74% rate). 1796 respondents completed the PHQ-9 (87.9%), while 1796 also completed the GAD 7 (87.9%). The respondent breakdown was as follows: 38% pilots (729), 19% cabin crew (376), 11% air traffic control (210), 8% maintenance/engineering (152), with the remaining 29% spanning other aviation workers. Overall, the respondents can be described as male (70%—1361) and working full time (86%—1643). The respondents can be split into the following age brackets; < 25 (5%—94), 25–35 (28%- 552), 36–45 (30%—584), 46–55 (23%- 458) and 56–65 (12%—242). Respondents had worked in aviation-related roles for the following lengths of time; < 2 years (3%—67), 2–5 years (15%—297), 6–10 years (18.5%—361), 11–15 years (14%—268), 16–20 years (12%—227), 21–25 years (12%- 244), 26–30 years (8%- 152) and > 30 years (17%—339).

#### COVID survey 2: 2021

1172 respondents participated in the survey. 1010 respondents completed the PHQ-9, and 1010 respondents completed the GAD 7. The respondent breakdown was as follows: in relation to roles, the breakdown was as follows: 48.33% pilots (*n* = 535), 22.22% cabin crew (*n* = 246), 13.1% air traffic control (*n* = 145), 6.96% maintenance/engineering (*n* = 77), with the remaining 9.39% spanning other aviation workers. Respondents can be characterized as mostly male (69.21%, *n* = 771), aged between 46 and 55 years (31.78%, *n* = 354), working in aviation for approx. 21–25 years (21.27%, *n* = 237), working for commercial airlines (73.07%, *n* = 814), specifically full-service carriers (72.06%, *n* = 575), working as pilots (48.33%, *n* = 535) and in permanent positions (80.27%, *n* = 883).

### Appendix 3: PHQ 9 cut-offs

The Patient Health Questionnaire-9 (PHQ-9) is the 9-item depression module from the full PHQ. It is a multi-purpose, self-reporting instrument for screening and assessing depression. Respondents self-rated the frequency of a variety of depressive symptoms within the past 2 weeks along a 4-point scale, as follows: 0 = “not at all,” 1 = “several days,” 2 = “more than half the days,” and 3 = “nearly every day.” Total scores ranged from 0 to 27. Major depression is diagnosed if 5 or more of the 9 depressive symptom criteria have been present at least “more than half the days” in the past 2 weeks, and 1 of the symptoms is depressed mood or anhedonia. Other depression is diagnosed if 2, 3, or 4 depressive symptoms have been present at least “more than half the days” in the past 2 weeks, and 1 of the symptoms is depressed mood or anhedonia. One of the 9 symptom criteria (“thoughts that you would be better off dead or of hurting yourself in some way”) counts if present at all, regardless of duration. Overall, a score of 10 is recommended as the cut-off score for diagnosing presence of depression.

**Table:**

Score	Depression severity
0–4	Minimal/none
5–9	Mild
10–14	Moderate
15–19	Moderately severe
20–27	Severe

Item 9, the final item of PHQ-9, is generally used in research to determine the existence of suicidal ideation—evaluating both the thought of passive death and the desire for self-harm in a single-response question. Question 9 of the PHQ9 asks about thoughts of being better off dead or of hurting oneself in some way over the last two weeks. Response options include “not at all” (Q9 = 0), “several days” (Q9 = 1), “more than half the days” (Q9 = 2), or “nearly every day” (Q9 = 3).

### Appendix 4: GAD 7 cut-offs

This GAD 7 is a self-administered patient questionnaire used as a screening tool and severity measure for generalised anxiety disorder (GAD). The GAD-7 score is calculated by assigning scores of 0, 1, 2, and 3, to the response categories of “not at all,” “several days,” “more than half the days,” and “nearly every day,” respectively, and then adding together the scores for the seven questions. A score of 10 or greater on the GAD-7 indicates GAD.

**Table:**

Score	Depression severity
0–4	Minimal
5–9	Mild
10–14	Moderate
15–21	Severe

### Appendix 5: summary of regression models

See Table 5.

**Table 5:** Regression models, variables and total number of observations

Model #	*X*/predictor variable	Dummy variable (1, 0), for *X*	*Y*/outcome variable	Number of observations
1	Spoken to somebody about a mental health issue experiencing/have experienced	1 = yes, 0 = no	PHQ 9	*n* = 995
2			Happiness and life satisfaction	
3			GAD 7	
4	Airline provided supports	1 = yes, 0 = no	PHQ 9	*n* = 965
5			Happiness and life satisfaction	
6			GAD 7	
7	Company cares about wellbeing	1 = strongly agree, 0 = all others (agree, neither, disagree, strongly disagree),	PHQ 9	*n* = 955
8			Happiness and life satisfaction	
9			GAD 7	
10	Would look for help if had MH issue	1 = strongly agree, 0 = all others (agree, neither, disagree, strongly disagree)	PHQ 9	*n* = 995
11			Happiness and life satisfaction	
12			GAD 7	
13	Willingly disclose MH issue to employer	1 = strongly agree, 0 = all others (agree, neither, disagree, strongly disagree)	PHQ 9	*n* = 522
14			Happiness and life satisfaction	
15			GAD 7	
16	Using coping strategies and scores	1 = yes, 0 = no	PHQ 9	*n* = 965
17			Happiness and life satisfaction	
18			GAD 7	
19	Use of company supports	1 = yes, 0 = no	PHQ 9	*n* = 202
20			Happiness and life satisfaction	
21			GAD 7	
22	Use of outside supports and scores	1 = yes, 0 = no	PHQ 9	*n* = 958
23			Happiness and life satisfaction	
24			GAD 7	
25	Changes in morale arising from the COVID-19 pandemic have negatively impacted on safety practices	1 = strongly agree, 0 = all others	PHQ 9	*n* = 917
26			Happiness and life satisfaction	
27			GAD 7	
28	Competence and ability to do your job safely and to the required standard now, as compared to before the COVID-19 pandemic	1 = deteriorated, 0 = all others	PHQ 9	917
29			Happiness and life satisfaction	
30			GAD 7	
31	Compliance with safety policies and procedures now, as compared to before the COVID-19 pandemic and scores	1 = deteriorated, 0 = all others	PHQ 9	917
32			Happiness and life satisfaction	
33			GAD 7	
34	Motivation towards their job now, as compared to before the COVID-19 pandemic and scores	1 = deteriorated, 0 = all others	PHQ 9	916
35			Happiness and life satisfaction	
36			GAD 7	

### Appendix 6: happiness and life satisfaction (measures)

**Table:** Comparison of Scores 2020 and 2021

	Min	1st Qu	Median/(middle)	Mean	3rd Qu	IQR	Max
Score 2020 (all)	1	5	6	6.086	8	3	10
Score 2021 (all)	1	5	6	6.089	8	3	10

**Table:** Breakdown of scores/different roles—2020

	Min	1st Qu (25% fall below)	Median/(middle)	Mean	3rd Qu (75% fall below)	Max
Score 2020	1	5	6	6.086	8	10
Pilots	1	5	6	6.176	8	10
Cabin crew	1	3.000	5.000	4.904	7	10
Maintenance/engineering	1	5	7	6.303	8	10
ATC	1	6	7	6.818	8	10

**Table:** Breakdown of scores/different roles—2021

	Min	1st Qu (25% fall below)	Median/(middle)	Mean	3rd Qu (75% fall below)	Max
Score 2021	1	5	6	6.089	8	10
Pilots	1	5	6	6.158	8	10
Cabin crew	1	4	5	5.494	7	10
Maintenance/engineering	2	5	7	6.09	8	10
ATC	2	6	7	6.644	8	10

### Appendix 8: PHQ 9—suicidal ideation

**Table:**

	Min	1st Qu	Median	Mean	3rd Qu	IQR	Max
Score 2021	0.0000	0.0000	0.0000	0.1416	0.0000	0	3.0000
Score 2020	0.0000	0.0000	0.0000	0.1687	0.0000	0	3.0000

### Appendix 10: breakdown of organisational supports (2020 and 2021 survey)

**Table:**

#	Category	Sub-category	Types	2020 (*n* = 335)	2021 (*n* = 116)
1	Primary	Workplace design/policy	COVID safety protocols	0.29% (*n* = 1)	0%
			Flexible work hours	0.59% (*n* = 2)	0%
			Better hotels/accommodation facilitates (sleep)	0.29% (*n* = 1)	0%
			Workload changes	0.29% (*n* = 1)	0%
			Flexible leave—visit family	0.29% (*n* = 1)	0%
			Roster flexibility—suit family life/preferences		
			Roster/bidding—flexibility		
			Work environment		
			Access to kitchen/cooking facilities in work		
		Wellbeing apps		1.49% (*n* = 5)	1.72, (*n* = 2)
		Wellbeing programs focussing on maintaining positive wellbeing and preventing illness	Wellbeing clinics	0.59% (*n* = 2)	0%
			Yoga class	0.29% (*n* = 1)	0%
			Company meetups/socials—runs, coffee mornings	0.59% (*n* = 2)	0.86% (*n* = 1)
			Exercise class	0.59% (*n* = 2)	1.72, (*n* = 2)
			Company clubs—chess, etc.	0.29% (*n* = 1)	0%
			Sponsored gym membership or access to gym	0%	0.86% (*n* = 1)
			Wellbeing challenges		
			Vaccination clinics/flu injections		
			Free healthy food		
		Gathering feedback (individual level)—making changes to better manage WRS/situation		0.29% (*n* = 1)	0%
		Gathering data (all staff) about issues/challenges to assess potential risk and identify risk mitigation strategies & measures			
		Health screening			
		Ergonomics assessments (musculoskeletal)			
		Health risk assessments			
2	Secondary	Health education and training	Training courses and workshops—managing stress, lifestyle and wellness coaching, awareness of MH. Mindfulness (online, webinars)	10.74 (*n* = 36)	14.65% (*n* = 17)
			Access to resources and information (links, booklets, videos, documents, guides)	12.23% (*n* = 41)	13.79% (*n* = 16)
		Emails/communications promoting wellbeing	Management communications, communications from occupational health and safety and HR	16.41% (*n* = 55)	17.24% (*n* = 20)
		2-way team meetings to communicate updates and provide feedback/support		12.83% (*n* = 43)	13.79% (*n* = 16)
		1 to 1 check-in/chats		1.49% (*n* = 5)	2.58% (*n* = 3)
		Signposting (internal and external)		12.83% (*n* = 43)	11.2% (*n* = 13)
3	Tertiary	Specialist MH support	1 to 1 psychological support/sessions with trained professionals—web or telephone/hotline or in person	22.08% (*n* = 74)	18.10% (*n* = 21)
		GP/physical health support		1.19% (*n* = 4)	0%
		Occupational health support		0.89%, (*n* = 3)	0.86% (*n* = 1)
		HR support		0.59% (*n* = 2)	0.86% (*n* = 1)
		EAP program (within/outside company)		15.52% (*n* = 52)	12.93%(*n* = 15)
		MH first aid		0.89%, (*n* = 3)	0%, (*n* = 0)
		Peer support		12.23% (*n* = 41)	35.34% (*n* = 41)
		Return to work programs		0.29% (*n* = 1)	0%
		HIMS programme (alcohol/substance abuse)		0.29% (*n* = 1)	0%
		CISM programme		0.89%, (*n* = 3)	0%
		Time off work/leave arrangements		0.59% (*n* = 2)	0%
		Access to support groups			
		Disease management programs			
		Health lifestyle/support programs	Smoking cessation programs		
			Weight loss programs		
			Healthy eating programs		

### Appendix 11: high-level results of statistical tests/regression analysis

**Table:**

Model #	Model	Test result	Significant relationship
1	Y (Depression) = β0 + β1 (talked to somebody) + e	Reject null hypothesis	Yes
2	Y (Life Satisfaction and happiness) = β0 + β1 (talked to somebody) + e	Reject null hypothesis	Yes
3	Y (Anxiety) = β0 + β1 (talked to somebody) + e	Reject null hypothesis	Yes
4	Y (Depression) = β0 + β1 (airline provided supports) + e	Reject null hypothesis	Yes
5	Y (Life Satisfaction and happiness) = β0 + β1(airline provided supports) + e	Reject null hypothesis	Yes
6	Y (Anxiety) = β0 + β1(airline provided supports) + e	Reject null hypothesis	Yes
7	Y (Depression) = β0 + β1 (Company Cares about wellbeing) + e	Reject null hypothesis	Yes
8	Y (Life Satisfaction and happiness) = β0 + β1(Company Cares about wellbeing) + e	Reject null hypothesis	Yes
9	Y (Anxiety) = β0 + β1(Company Cares about wellbeing) + e	Reject null hypothesis	Yes
10	Y (Depression) = β0 + β1 (Would look for help if had MH issue) + e	Reject null hypothesis	Yes
11	Y (Life Satisfaction and happiness) = β0 + β1(Would look for help if had MH issue) + e	Reject null hypothesis	Yes
12	Y (Anxiety) = β0 + β1(Would look for help if had MH issue) + e	Fail to reject the NULL hypothesis	No
13	Y (Depression) = β0 + β1 (Willingly disclose MH issue to employer) + e	Reject null hypothesis	Yes
14	Y (Life Satisfaction and happiness) = β0 + β1 (Willingly disclose MH issue to employer) + e	Reject null hypothesis	Yes
15	Y (Anxiety) = β0 + β1 (Willingly disclose MH issue to employer) + e	Reject null hypothesis	Yes
16	Y (Depression) = β0 + β1 (Using coping strategies and scores) + e	Fail to reject the NULL hypothesis	No
17	Y (Life Satisfaction and happiness) = β0 + β1(Using coping strategies and scores) + e	Fail to reject the NULL hypothesis	No
18	Y (Anxiety) = β0 + β1(Using coping strategies and scores) + e	Fail to reject the NULL hypothesis	No
19	Y (Depression) = β0 + β1 (Use of company supports) + e	Fail to reject the NULL hypothesis	No
20	Y (Life Satisfaction and happiness) = β0 + β1(Use of company supports) + e	Fail to reject the NULL hypothesis	No
21	Y (Anxiety) = β0 + β1(Use of company supports) + e	Fail to reject the NULL hypothesis	No
22	Y (Depression) = β0 + β1 (Use of supports outside company) + e	Reject null hypothesis	Yes
23	Y (Life Satisfaction and happiness) = β0 + β1(Use of supports outside company) + e	Reject null hypothesis	Yes
24	Y (Anxiety) = β0 + β1(Use of supports outside company) + e	Reject null hypothesis	Yes
25	Y (Depression) = β0 + β1 (Changes In Morale and Negative Impact on Safety Practices) + e	Reject null hypothesis	Yes
26	Y (Life Satisfaction and happiness) = β0 + β1(Changes In Morale and Negative Impact on Safety Practices) + e	Reject null hypothesis	Yes
27	Y (Anxiety) = β0 + β1(Changes In Morale and Negative Impact on Safety Practices) + e	Reject null hypothesis	Yes
28	Y (Depression) = β0 + β1 (Competence & Ability To Do Job Safely) + e	Fail to reject the NULL hypothesis	No
29	Y (Life Satisfaction and happiness) = β0 + β1(Competence & Ability To Do Job Safely) + e	Fail to reject the NULL hypothesis	No
30	Y (Anxiety) = β0 + β1 Competence & Ability To Do Job Safely) + e	Reject null hypothesis	Yes
31	Y (Depression) = β0 + β1 (Compliance with Safety Procedures) + e	Fail to reject the NULL hypothesis	No
32	Y (Life Satisfaction and happiness) = β0 + β1(Compliance with Safety Procedures) + e	Reject null hypothesis	Yes
33	Y (Anxiety) = β0 + β1(Compliance with Safety Procedures) + e	Fail to reject the NULL hypothesis	No
34	Y (Depression) = β0 + β1 (Motivation Towards the Job) + e	Reject null hypothesis	Yes
35	Y (Life Satisfaction and happiness) = β0 + β1(Motivation Towards the Job) + e	Reject null hypothesis	Yes
36	Y (Anxiety) = β0 + β1(Motivation Towards the Job) + e	Reject null hypothesis	Yes

### Appendix 12: detailed results of statistical tests/regression analysis: R squared, coefficient values and *p* values

**Table:**

Model #	*X*/predictor variable	*Y*/outcome variable	*R*^2^	Coef/β1	*p* value	Confidence interval for β1
1	Spoken to somebody about a mental health issue experiencing/have experienced	PHQ 9	0.02474	1.6419	6.17e−07	(0.9998942, 2.283927)
2		Happiness and life satisfaction	0.004772	− 0.27748	0.0293	(− 0.5270191, 0.02793517)
3		GAD 7	0.01981	1.4483	8.35e−06	(0.8138406,2.082717)
4	Airline provided supports	PHQ 9	0.008049	− 1.1420	0.00529	(− 1.943767 − 0.3403156)
5		Happiness and life satisfaction	0.009555	0.47750	0.00237	(0.1700606, 0.7849458)
6		GAD 7	0.004891	− 0.8731	0.0298	(− 1.660631 − 0.08558235)
7	Company cares about wellbeing	PHQ 9	0.00956	− 2.8776	0.00249	(− 4.739546, − 1.015656)
8		Happiness and life satisfaction	0.02584	1.8084	5.91e−07	(1.102604, 2.514209)
9		GAD 7	0.008743	− 2.6923	0.00383	(− 4.514647, − 0.8698663)
10	Would look for help if had MH issue	PHQ 9	0.00633	− 0.9816	0.0121	(− 1.747529,− 0.2157201)
11		Happiness and life satisfaction	0.003819	0.64724	1.59e−05	(− 1.507504, 0.004319308)
12		GAD 7	0.003819	− 0.7516	0.0513	(− 1.507504, 0.004319308)
13	Willingly disclose MH issue to employer	PHQ 9	0.04133	− 2.5101	2.83e−06	(0.9998942, 2.283927)
14		Happiness and life satisfaction	0.04091	0.98866	3.19e−06	(0.5762614, 1.401049)
15		GAD 7	0.03804	− 2.4151	7.16e−06	(− 3.461368, − 1.368884)
16	Using coping strategies and scores	PHQ 9	0.0009689	0.08977	0.796	(− 0.5914199, 0.7709523)
17		Happiness and life satisfaction	0.0004108	− 0.08379	0.796	(− 0.5914199, 0.7709523)
18		GAD 7	0.0007115	0.2818	0.408	(− 0.386037, 0.9496488)
19	Use of company supports	PHQ 9	0.0003025	0.1947	0.806	(− 1.754963, 1.365663)
20		Happiness and life satisfaction	9.687e−06	− 0.01286	0.965	(− 0.5887809, 0.563070)
21		GAD 7	1.903e−05	0.05051	0.951	(− 1.564085 1.66511)
22	Use of outside supports	PHQ 9	0.04251	2.4340	1.17e−10	(1.700869, 3.167154)
23		Happiness and life satisfaction	0.004635	− 0.30761	0.0351	(− 0.5937179 − 0.02149422)
24		GAD 7	0.05328	2.6716	4.72e−13	(1.956814, 3.386337)
25	Changes in morale arising from the COVID-19 pandemic have negatively impacted on safety practices	PHQ 9	0.05032	2.7417	6.37e−12	(1.968932,3.514528)
26		Happiness and life satisfaction	0.0479	− 1.02112	2.08e−11	(− 1.316471, 0.7257614)
27		GAD 7	0.03704	2.3016	4.23e−09	(1.54020, 3.063033)
28	Competence and ability to do the job safety	PHQ 9	0.002153	0.5174	0.16	(− 0.2052992, 1.240173)
29		Happiness and life satisfaction	0.00341	− 0.24855	0.0772	(− 0.5242498, 0.02714508)
30		GAD 7	0.008084	0.9810	0.00644	(0.2759634, 1.686078)
31	Compliance with safety policies and procedures now, as compared to before the COVID-19 pandemic	PHQ 9	0.002534	0.6098	0.128	(− 0.17508, 1.394623)
32		Happiness and life satisfaction	0.004642	− 0.31500	0.0391	(− 0.6142625, − 0.0157318)
33		GAD 7	0.003152	0.6654	0.0893	(− 0.1023014, 1.433106)
34	Motivation towards their job now, as compared to before the COVID-19 pandemic	PHQ 9	0.00735	0.9155	0.00943	(0.2248148, 1.606128)
35		Happiness and life satisfaction	0.005251	− 0.29549	0.0283	(− 0.5595142, − 0.03147102)
36		GAD 7	0.005317	0.7613	0.0273	(0.08536097, 1.437260)
